# In-Person vs Electronic Directly Observed Therapy for Tuberculosis Treatment Adherence: A Randomized Noninferiority Trial

**DOI:** 10.1001/jamanetworkopen.2021.44210

**Published:** 2022-01-20

**Authors:** Joseph Burzynski, Joan M. Mangan, Chee Kin Lam, Michelle Macaraig, Marco M. Salerno, B. Rey deCastro, Neela D. Goswami, Carol Y. Lin, Neil W. Schluger, Andrew Vernon

**Affiliations:** 1 Bureau of Tuberculosis Control, New York City Department of Health and Mental Hygiene, Queens, New York; 2 Division of Tuberculosis Elimination, US Centers for Disease Control and Prevention, Atlanta, Georgia; 3 Division of Pulmonary, Allergy & Critical Care, Columbia University, New York, New York

## Abstract

**Importance:**

Electronic directly observed therapy (DOT) is used increasingly as an alternative to in-person DOT for monitoring tuberculosis treatment. Evidence supporting its efficacy is limited.

**Objective:**

To determine whether electronic DOT can attain a level of treatment observation as favorable as in-person DOT.

**Design, Setting, and Participants:**

This was a 2-period crossover, noninferiority trial with initial randomization to electronic or in-person DOT at the time outpatient tuberculosis treatment began. The trial enrolled 216 participants with physician-suspected or bacteriologically confirmed tuberculosis from July 2017 to October 2019 in 4 clinics operated by the New York City Health Department. Data analysis was conducted between March 2020 and April 2021.

**Interventions:**

Participants were asked to complete 20 medication doses using 1 DOT method, then switched methods for another 20 doses. With in-person therapy, participants chose clinic or community-based DOT; with electronic DOT, participants chose live video-conferencing or recorded videos.

**Main Outcomes and Measures:**

Difference between the percentage of medication doses participants were observed to completely ingest with in-person DOT and with electronic DOT. Noninferiority was demonstrated if the upper 95% confidence limit of the difference was 10% or less. We estimated the percentage of completed doses using a logistic mixed effects model, run in 4 modes: modified intention-to-treat, per-protocol, per-protocol with 85% or more of doses conforming to the randomization assignment, and empirical. Confidence intervals were estimated by bootstrapping (with 1000 replicates).

**Results:**

There were 173 participants in each crossover period (median age, 40 years [range, 16-86 years]; 140 [66%] men; 80 [37%] Asian and Pacific Islander, 43 [20%] Black, and 71 [33%] Hispanic individuals) evaluated with the model in the modified intention-to-treat analytic mode. The percentage of completed doses with in-person DOT was 87.2% (95% CI, 84.6%-89.9%) vs 89.8% (95% CI, 87.5%-92.1%) with electronic DOT. The percentage difference was −2.6% (95% CI, −4.8% to −0.3%), consistent with a conclusion of noninferiority. The 3 other analytic modes yielded equivalent conclusions, with percentage differences ranging from −4.9% to −1.9%.

**Conclusions and Relevance:**

In this trial, the percentage of completed doses under electronic DOT was noninferior to that under in-person DOT. This trial provides evidence supporting the efficacy of this digital adherence technology, and for the inclusion of electronic DOT in the standard of care.

**Trial Registration:**

ClinicalTrials.gov Identifier: NCT03266003

## Key Points

**Question:**

Is electronic directly observed therapy (DOT) noninferior to in-person DOT in supporting medication adherence for tuberculosis treatment?

**Findings:**

In this randomized, 2-period crossover noninferiority trial of 216 patients with tuberculosis, the modified intention-to-treat analysis estimate of the percentage of medication doses staff observed patients ingest with in-person DOT was 87.2% vs 89.8% with electronic DOT. The percentage difference between DOT methods was −2.6%, which was less than the noninferiority margin of 10% at a statistically significant level.

**Meaning:**

These findings suggest that electronic DOT was noninferior to in-person DOT when employed by a tuberculosis program that has historically implemented in-person DOT successfully.

## Teaser

This randomized noninferiority trial compares the effectiveness of electronic directly observed therapy for tuberculosis treatment regimen with in-person observation.

## Introduction

In the US, directly observed therapy (DOT) is a key component of tuberculosis (TB) control. During DOT, TB program staff observe patients ingest medication in locations convenient to patients.^1,2,3,4,5,6,7,8,9,10,11^ This approach is costly and poses logistical challenges for TB programs and patients.^12,13^ In response, programs have sought to capitalize on advances in communication technology to develop alternatives to in-person DOT. Use of one such approach, electronic DOT, has steadily increased in recent years.^14^ Electronic DOT employs personal electronic devices, particularly smart mobile telephones with video capabilities, to view patients remotely ingest their medications.

The US Centers for Disease Control and Prevention (CDC) and World Health Organization’s (WHO) Global Task Force on Digital Health for TB promote the use of digital technologies to address challenges in TB prevention, care, and control in a patient-centered manner.^15,16^ Robust evidence across different population groups to support these recommendations is limited. A recent review of digital technology to enhance TB control identified 19 studies that reported electronic DOT was feasible, acceptable, and associated with good treatment adherence; most of these studies were observational.^17^

Three randomized trials have reported higher levels of treatment observation,^18,19^ comparable treatment completion rates,^20^ lower program-incurred costs,^18,20^ lower patient-incurred costs,^19^ and greater satisfaction^19,20^ among patients who used electronic compared with patients randomized to in-person DOT. These results are encouraging. However, it is important to note that 2 of the 3 trials used only clinic-based in-person DOT as a comparator.^19,20^ Multiple studies have demonstrated that when patients are directed to undergo DOT in clinical facilities, the studied cohorts often have lower treatment completion rates,^21,22,23,24^ less treatment success,^24^ increased mortality,^24^ less satisfaction with their care,^21^ and higher out-of-pocket costs^21^ compared with patients who undergo DOT in a more patient-centered manner, such as within their homes or in community-based settings.^24,25^ The Story et al^18^ trial randomized participants to either clinic-, home-, and community-based in-person or electronic DOT using patient-recorded videos asynchronously viewed by treatment observers. This trial included a large percentage of patients with social features often associated with poor adherence. While this trial demonstrated that electronic DOT was advantageous for a difficult-to-reach population, overall adherence was low for participants in either group.^18^

We sought to determine whether electronic DOT could achieve as high a level of treatment observation in a large multiclinic TB control program as could be achieved with in-person DOT conducted at patient-preferred locations. Implemented under pragmatic conditions with a diverse patient population in a large urban TB program, the purpose of our study was to expand the knowledge base on digital adherence technologies.

## Methods

### Objective and Study Design

Our primary objective was to assess the difference in the percentage of medication doses that staff observed participants completely ingest with electronic vs in-person DOT. We used a randomized, 2-arm, 2-period crossover, noninferiority design. This trial was conducted in 4 clinics operated by the New York City (NYC) Department of Health and Mental Hygiene, Bureau of Tuberculosis Control (BTBC). The trial protocol (Supplement 1) was approved by institutional review boards at the NYC Department of Health and Mental Hygeine and Columbia University. All participants provided written informed consent. The study followed the Consolidated Standards of Reporting Trials (CONSORT) reporting guideline.

### Study Participants

Enrollment began July 2017 and ended October 2019. We included persons aged 12 years and older, with a suspected, laboratory-confirmed, or clinical diagnosis of TB disease, who were prescribed oral anti-TB medication,^26^ had a residence location accessible to staff, and had no plans to move for 9 months. We recruited English and non-English speaking participants using bilingual staff, contracted interpreters, and translated data collection forms. To assess whether participants were similar across analytic groups and representative of the NYC BTBC patient population, participants’ self-reported race and ethnicity data were retrieved from their Department of Health and Mental Hygeine electronic medical record. Persons were excluded if prescribed injectable TB medications or the supervising physician advised use of in-person DOT. We also excluded those with a cognitive or physical disability that prevented their use of electronic DOT who lacked a caretaker to assist them.

### Randomization

Participants were randomized 1:1 to start outpatient treatment with either in-person or electronic DOT using a computer-generated random list for each clinic. This list was used to create numbered and sealed opaque randomization packets for each clinic. Following each participant enrollment, staff opened packets sequentially to make group assignments. Those randomized to group 1 underwent in-person DOT during crossover period 1, followed by electronic DOT during period 2. For group 2 participants, the order of DOT methods was reversed.

### Study Measurements and Procedures

For this study, all nonholiday, weekday doses scheduled in advance for DOT were designated scheduled and observable, and an outcome was documented for each dose. The NYC BTBC routinely provided DOT on weekdays only. Each crossover period comprised 20 scheduled and observable doses. If a treating clinician held all medications, the scheduled and observable criteria could not be met, and these doses were excluded from analysis. Similarly, doses were excluded if a participant was admitted to a medical or correctional facility.

If a problem arose during a DOT session, the type of problem encountered (eg, technical-, patient-, or program-related) and reasons for nonobservation were documented. At the conclusion of the 2 crossover periods, participants chose their preferred DOT method for their remaining treatment.

Participants undergoing in-person DOT could choose to meet with health department staff at the TB clinic (clinic-based DOT) or at a mutually agreed-upon location in the community (community-based DOT). While undergoing electronic DOT, participants could choose live videoconferencing (Skype for Business), which allowed TB program staff to interact with participants in real-time, or recorded (asynchronous) videos using a software application that automatically uploaded time-stamped videos to a secure cloud-based server (SureAdhere Mobile Technology, Inc), and which TB program staff reviewed the following workday. To ensure participants’ competency with electronic DOT software applications, a standardized teach-back training method was used.

Analogous with BTBC practice, participants used personal smartphones or other video-capable devices (eg, tablet) to engage in electronic DOT. Participants who did not possess a device were loaned a smartphone by the BTBC at no charge. Those using personal devices were provided a $10 gift card each month to reimburse data usage costs. Additionally, all participants were provided $50 for completing the study’s enrollment visit, and another $50 if they completed an opinion questionnaire following the 2 crossover periods.

Participant care was coordinated according to BTBC case management policies.^26^ Treatment was prescribed and provided at no cost to the patient according to New York State law.

### Sample Size Calculation

We computed a sample size under a parallel design, then modified the computation to account for pooled variances^27^ and reduced the sample size to account for the effect of the crossover design.^28^ We estimated that 256 participants were required to determine, with 90% power and a two-sided significance level of 2.5%, whether electronic DOT is noninferior to in-person DOT, using a prespecified noninferiority margin of 10%. This margin was based on the presumption that electronic DOT would be of interest to programs because of logistical and cost advantages, even if adherence was slightly worse than when DOT is conducted in-person.

### Statistical Analysis

Each scheduled and observable dose of medication was classified with a binary outcome: staff observed the participant completely ingest the dose of medication (hereafter called a “completed dose”), or they did not. The binary dose outcomes were analyzed using a logistic generalized linear mixed effects regression model (GLMM),^29^ which included fixed-effect explanatory variables representing DOT method at each dose, participant randomization group, crossover period, the dose outcome during each of the 2 preceding scheduled and observable doses (representing carryover effects), season (represented as calendar quarter), and the interaction between DOT method and season. To minimize bias from expected correlations among doses observed within the same participant and among participants treated at the same clinic, the GLMM included random effects representing each tuberculosis treatment clinic and each participant nested within their respective clinic. Participants were included in the primary statistical analysis if they completed both crossover periods with sufficient data to represent carryover effects in the GLMM (eAppendix 2 in Supplement 2).

The percentages of completed doses with electronic and in-person DOT were estimated as least-square means from the GLMM. The percentage difference was calculated by subtracting the percentage of completed doses observed with electronic DOT from the percentage with in-person DOT. Robust estimates of percentages, percentage differences, and confidence limits were obtained with the bootstrap method by repeating the described calculations for 1000 replicate data sets.^30^ To test for noninferiority, the bootstrap 95% upper confidence limit of the percentage difference was compared with the designated 10% noninferiority margin; a 95% upper confidence limit less than the noninferiority margin is consistent with a conclusion of noninferiority at a 5% confidence level. The GLMM was run in 4 analytic modes: modified intention-to-treat (ITT), empirical (ie, as-observed; EMP), per-protocol (PP), and PP 85%. In the modified ITT analysis, the DOT method of each dose was represented according to participants’ randomization assignment. This ITT mode was described as modified because it excluded 38 participants postrandomization who lacked sufficient data to represent carryover effects in the GLMM. In the EMP mode, the DOT method of each dose was represented according to the DOT method actually used. The PP analysis was restricted to participants whose DOT method at each dose matched their randomization assignment. The PP 85% analysis was restricted to patients with 85% or more doses that matched their randomization assignment; doses that did not match randomization were represented according to the DOT method the participant used. Additional details about the statistical analysis and sensitivity analyses are provided in Appendix 1 in Supplement 2.

## Results

### Demographic Characteristics of Enrolled Patients and Analytical Samples

**Figure 1.**
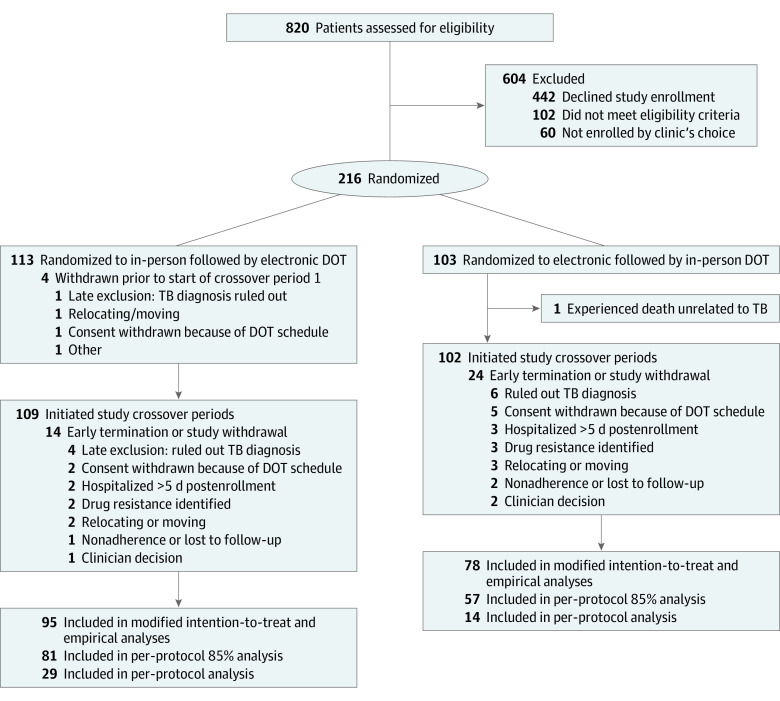
Study Flowchart

A total of 216 persons were randomized (median age, 42 years [range, 16-86 years]; 140 [65%] men) among 820 persons screened (Figure 1; eTable 2 in Supplement 2). Five participants (2%) withdrew before crossover period 1 commenced and were unavailable for analysis, and another 38 (18%) withdrew during crossover period 1. The remaining 173 participants completed both crossover periods and were included in the modified ITT and EMP analyses.

 The demographics of participants included in the modified ITT (173 participants), PP (43 participants), and PP 85% (138 participants) analyses were similar to nonenrolled patients who underwent TB treatment through the NYC BTBC during the study period (Table 1). Overall, the distribution of racial and ethnic groups was similar (total enrolled: 43 [20%] non-Hispanic African American or Black individuals; 80 [37%] Asian, Pacific Islander, and Hawaiian individuals; 71 [33%] Hispanic individuals; and 9 [4%] non-Hispanic White individuals), although proportionally more persons of Asian descent were enrolled, and fewer Hispanic persons were included in the PP analysis (11 [26%] individuals). Characteristics of the randomized groups were generally similar. Proportionally more persons 61 years and older were randomized to group 2 (eTable 3 in Supplement 2).

**Table 1.** Demographics and Clinical Characteristics of Enrolled Participants and Participants Within Each Analytic Group

Characteristic	Participants, No. (%)				
	Eligible persons not enrolled^a^ (n = 886)	All enrolled (n = 216)	Modified ITT and EMP analysis (n = 173)	Per-protocol analysis (n = 43)	Per protocol 85% analysis (n = 138)
Sex					
Men	512 (58)	140 (65)	114 (66)	24 (56)	90 (65)
Women	373 (42)	76 (35)	59 (34)	19 (44)	48 (35)
Unknown sex	1 (<0.1)	0	0	0	0
Age, median (range), y	48 (12-96)	42 (16-86)	40 (16-86)	41 (16-73)	39 (16-86)
Age group, y					
≤15	9 (1)	0	0	0	0
16-20	40 (5)	10 (5)	8 (5)	3 (7)	6 (4)
21-30	144 (16)	57 (26)	49 (28)	10 (23)	39 (28)
31-40	150 (17)	32 (15)	29 (17)	7 (16)	24 (17)
41-50	119 (13)	38 (17)	32 (18)	11 (26)	25 (18)
51-60	152 (17)	41 (19)	30 (17)	8 (19)	23 (17)
61-70	142 (16)	21 (10)	13 (8)	1 (2)	11 (8)
71-80	80 (9)	15 (7)	10 (6)	3 (7)	9 (7)
81-90	47 (5)	2 (1)	2 (1)	0	1 (1)
≥91	3 (0.3)	0	0	0	0
Birthplace					
US born	103 (11)	27 (12)	20 (12)	3 (7)	18 (13)
Non-US born	777 (88)	187 (87)	151 (87)	40 (93)	120 (87)
Unknown/missing	6 (1)	2 (1)	2 (1)	0	0
Region of birth					
Africa	82 (9)	18 (8)	17 (10)	3 (7)	16 (12)
Asia	402 (45)	84 (39)	68 (39)	21 (49)	51 (37)
Caribbean	132 (15)	31 (14)	22 (13)	2 (5)	15 (11)
Central America	30 (3)	6 (3)	5 (3)	0	4 (3)
Europe	20 (2)	7 (3)	6 (3)	2 (5)	5 (4)
North America	127 (14)	39 (18)	32 (18)	6 (14)	30 (22)
South America	86 (10)	29 (13)	21 (12)	9 (21)	17 (12)
Unknown/missing	7 (1)	2 (1)	2 (1)	0	0
Race and ethnicity^b^^,^^c^					
African American and Black, non-Hispanic	50 (49)^c^	43 (20)	35 (20)	5 (12)	32 (23)
Asian, Pacific Islander, and Hawaiian	5 (5)	80 (37)	64 (37)	21 (49)	48 (35)
Hispanic	36 (35)	71 (33)	55 (32)	11 (26)	44 (32)
Other/multiple^d^	3 (3)	13 (6)	11 (6)	4 (9)	8 (6)
White, non-Hispanic	9 (9)	9 (4)	8 (5)	2 (5)	6 (4)
Employed^e^					
Yes	331 (37)	124 (57)	101 (58)	23 (53)	82 (59)
No	NR	62 (29)	46 (27)	12 (28)	37 (27)
Unknown/missing	NR	30 (14)	26 (15)	8 (19)	19 (14)
Access to video device prior to enrollment					
Yes	NR	149 (69)	143 (83)	33 (77)	112 (81)
No	NR	67 (31)	30 (17)	10 (23)	26 (19)
Primary language spoken					
English	202 (23)	55 (25)	44 (25)	12 (28)	42 (30)
Spanish	212 (24)	56 (26)	41 (24)	8 (19)	33 (24)
Chinese (Cantonese, Fujianese, Mandarin)	163 (18)	24 (11)	21 (12)	7 (16)	19 (14)
French, Creole, pidgins, French-based other	50 (6)	16 (7)	15 (9)	2 (5)	13 (9)
Other	230 (26)	60 (28)	47 (27)	13 (30)	29 (21)
Unknown	29 (3)	5 (2)	5 (3)	1 (2)	2 (1)
Educational attainment					
No formal schooling	NR	12 (6)	9 (5)	1 (2)	7 (5)
Primary school (grades 1-5)	NR	9 (4)	6 (3)		4 (3)
Middle school (grades 6-8)	NR	27 (13)	22 (13)	4 (9)	17 (12)
Secondary school (grades 9-12)	NR	84 (39)	68 (39)	13 (30)	56 (41)
College or more	NR	62 (29)	49 (28)	21 (49)	41 (30)
Unknown/refused to answer	NR	22 (10)	19 (11)	4 (9)	13 (9)
Diagnosis setting					
Hospital	NR	80 (37)	67 (39)	13 (30)	53 (38)
Private practice	NR	4 (2)	3 (2)	1 (2)	3 (2)
Local/state health department	NR	101 (47)	81 (47)	23 (53)	63 (46)
Other (ie, correctional facility) or unknown	NR	31 (14)	22 (13)	6 (14)	19 (14)
TB disease, pulmonary	754 (85)	190 (88)	154 (89)	38 (88)	123 (89)
Known positive HIV status	43 (5)	8 (4)	5 (3)	1 (2)	5 (4)
Homeless within 12 mo of diagnosis	30 (3)	4 (2)	2 (1)	0	1 (1)
History of incarceration, ever	29 (3)	8 (4)	5 (3)	1 (2)	4 (3)
Excess alcohol use in past year	21 (2)	4 (2)	3 (2)	0	3 (2)
History of substance use	55 (6)	20 (9)	16 (9)	3 (7)	14 (10)
Reason medication stopped^f^					
Completion of treatment	659 (74)	150 (69)	133 (77)	31 (72)	108 (78)
TB diagnosis ruled out	94 (11)	33 (15)	21 (12)	9 (21)	15 (11)
Medication stopped due to adverse treatment event	5 (0.6)	1 (0.5)	0	0	0
Lost to follow-up	8 (1)	5 (2)	4 (2)	0	4 (3)
Death	3 (0.3)	2 (0.9)	1 (0.6)	0	0
Refused or uncooperative	13 (2)	NA	NA	NA	NA
Withdrew study consent	NA	7 (3)	2 (1)	0	2 (1)
Other (ie, treatment extended, medications held)	28 (3)	14 (7)	8 (5)	2 (5)	6 (4)
Not documented (eg, patient moved, other physician decision)	76 (9)	4 (2)	4 (2)	1 (2)	3 (2)

Abbreviations: NA, not applicable; NR, not reported; NYC, New York City; TB, tuberculosis.

^a^ Demographic information is provided for individuals who obtained TB care through the NYC Bureau of Tuberculosis Control clinics. In NYC, TB care is also delivered through NYC public health care hospitals, private hospitals and clinics, Veteran’s Administration hospitals and clinics, and the NYC Department of Corrections. Individuals receiving care through these facilities and clinics were not recruited for this study.

^b^ To assess whether participants were similar across analytic groups, participants’ race and ethnicity were obtained from clinic records.

^c^ In NYC race and ethnicity data are routinely collected for US-born patients only. Data for race and ethnicity for eligible persons not enrolled was limited to 103 participants.

^d^ Other/multiple denotes persons who identified as a combination of 2 or more fixed racial or ethnicity categories.

^e^ In last 24 months at time of study enrollment.

^f^ Reason documented as of August 2020.

### DOT Usage Patterns by Dose

**Figure 2.**
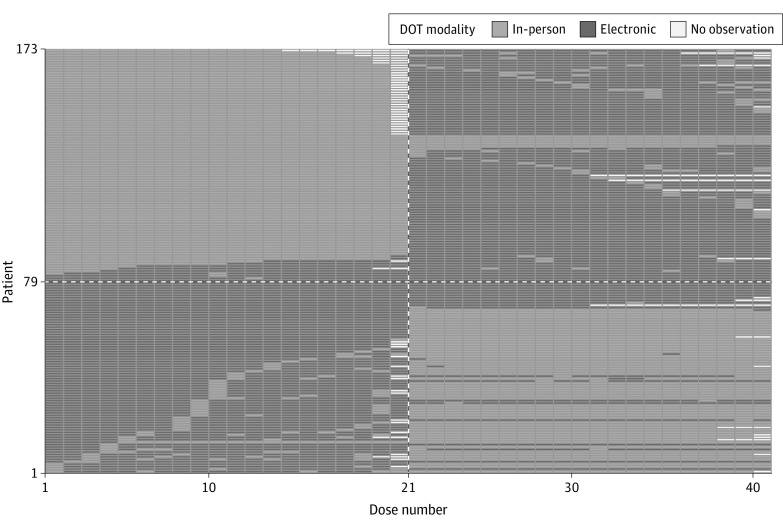
Patient Crossover Between In-person and Electronic DOT DOT indicates directly observed therapy.

In total, 138 (80%) participants switched DOT methods for crossover period 2 in accordance with study protocol (Figure 2). During crossover period 1, 2 (1%) group 1 participants elected to switch to electronic DOT prior to the start of crossover period 2. Further, 27 (16%) remained on electronic DOT and 6 (4%) remained on in-person DOT by their own choice for both crossover periods. Patients undergoing electronic DOT used in-person DOT intermittently during clinic appointments when they had not yet taken their medication.

### Effect of DOT Method on Dose Completion

**Figure 3.**
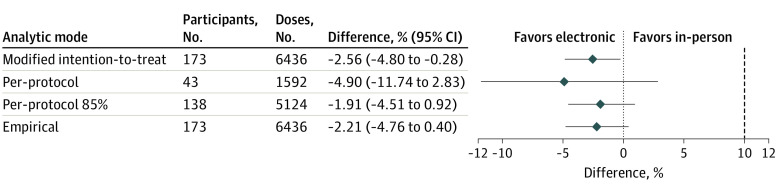
Percentage Difference of Electronic vs In-person Directly Observed Therapy Dashed vertical line indicates noninferiority margin.

Electronic DOT was noninferior compared with in-person DOT (Table 2 and Figure 3). In the modified ITT analytic mode (173 participants), the bootstrap percentage of completed doses with in-person DOT was 87.2% (95% CI, 84.6% to 89.9%) vs 89.8% (95% CI, 87.5% to 92.1%) with electronic DOT. The bootstrap percentage difference was −2.6% (95% CI, −4.8% to −0.3%). The upper 95% confidence limit of −0.3% was far less than the 10% noninferiority limit, which was consistent with electronic DOT being noninferior to in-person DOT in attaining dose completion. Results of the EMP (−2.2%; 95% CI, −4.8% to 0.4%), PP (−4.9%; 95% CI, −11.7% to 2.8%), and PP 85% (−1.9%; 95% CI, −4.5% to 0.9%) analyses were consistent with those of the modified ITT analysis and supported the conclusion that electronic DOT was noninferior to in-person DOT. Furthermore, the magnitude of the bootstrap percentage differences suggested that electronic DOT outperformed in-person DOT by 1.9% to 4.9%.

**Table 2.** Completed Doses and Percentage Differences Between Electronic vs In-person DOT by Analysis Mode

Variable	Completed doses, % (95% CI)^a^			
	Modified ITT (n = 173)	Empirical (n = 173)	Per-protocol	
			100% (n = 43)	85% (n = 138)
Scheduled observable doses, No.	6436	6436	1592	5124
In-person DOT				
Doses staff observed patients completely ingest/total doses, No.	2800/3192	2594/2979	668/790	2363/2699
Completed doses	87.2 (84.6 to 89.9)	87.3 (84.7 to 90.0)	84.6 (78.2 to 90.9)	87.3 (84.6 to 90.0)
Electronic DOT				
Doses staff observed patients completely ingest/total doses, No.	2914/3244	3120/3457	706/802	2166/2425
Completed doses	89.8 (87.5 to 92.1)	89.4 (86.8 to 91.9)	89.5 (82.5 to 95.2)	89.2 (86.5 to 92.0)
Percentage difference				
In-person to electronic difference	−2.6 (−4.8 to −0.3)	−2.2 (−4.8 to 0.4)	−4.9 (−11.7 to 2.8)	−1.9 (−4.5 to 0.9)
Electronic noninferior?^b^	Yes	Yes	Yes	Yes

Abbreviations: DOT, directly observed therapy.

^a^ Estimated with bootstrap logistic generalized linear mixed effects regression model.

^b^ Noninferiority limit is within 10% of the upper confidence interval.

The association of season with dose completion observed under electronic vs in-person DOT was evaluated with an interaction term in the GLMM. In each season, the upper 95% bootstrap confidence limit for the percentage difference was less than the 10% noninferiority limit for all 4 analytic modes, with 1 exception. In the PP analytic mode, the upper confidence limit for spring (April through June: 8.8%; 95% CI, −9.4% to 42.0%) exceeded the noninferiority limit (not tabulated). This was likely a result of the small numbers in this restricted mode (43 participants). Overall, for an urban area located in a temperate climate, season was not significantly associated with the percentage difference in dose completion (eTable 4 in Supplement 2).

To assess whether the conclusion of noninferiority depended on restricting analysis to 173 participants, GLMMs were rerun with 33 of 38 participants who withdrew during crossover period 1 and had data sufficient to represent carryover effects in the logistic GLMMs. This expanded sample comprised 206 participants (none of the excluded participants met the criteria for PP and PP 85% analytic modes), for which ITT and EMP analytic modes were run. Results from the expanded patient sample also supported the conclusion of noninferiority. In addition, the noninferiority conclusion was upheld in (unadjusted) univariate analyses (eTable 1, eFigure in Supplement 2).

### Technical, Patient, and Program Issues Affecting DOT Sessions

Issues affecting medication observations occurred with both electronic and in-person DOT. Among 29 900 prescribed medication doses taken during and after the crossover periods for all 216 participants, 20 344 were nonholiday, weekday doses scheduled for DOT. For 2034 (10%) DOT doses, 2239 unique problems were documented. Of these, 1083 (48%) were patient-related (eg, difficulties operating software or work schedules interfered with DOT), 688 (31%) were technical (eg, nonfunctioning internet connections), and 468 (21%) were staff- or program-related (eg, unscheduled absence). Overall, 1301 of the 2239 unique problems led to 1161 (57%) of the 2034 affected DOT observations not being observed. Community-based in-person DOT observations had the greatest percentage of issues with observations (541 observations [19%]) compared with live-video electronic DOT observations (714 [10%]), recorded-video electronic DOT (659 [8%]), and clinic-based in-person DOT (120 [6%]).

### Participants’ DOT Preferences for the Remainder of Treatment

Seventy-three (42%) of the 173 participants who completed the crossover period reported they preferred to continue treatment with live-video electronic DOT, 73 (42%) preferred recorded-video electronic DOT, 9 (5%) preferred community-based in-person DOT, 1 (0.6%) preferred clinic-based in-person DOT, and 6 (4%) elected to self-administer their medications. No preference was recorded for 11 (6%) participants, as a TB diagnosis was ruled out for 3, 4 completed treatment, 3 stopped medication for unspecified reasons, and 1 was lost to follow-up.

## Discussion

This trial enabled rigorous evaluation of electronic DOT efficacy under pragmatic conditions with a diverse patient population receiving treatment through an urban TB program. Our results demonstrate that, in this context, electronic DOT was noninferior to in-person DOT across multiple modes of statistical analysis. Moreover, the results rest on the strength of analysis at the level of individual doses, while controlling for biases arising from the study design.

As novel technologies are integrated into the delivery of medical care, potential exists for increasing the number of weekly doses observed using recorded electronic DOT; improving clinical outcomes; delivering patient-centered care; empowering patients; and promoting equity in care.^16,31^ These possibilities are significant. Poor treatment adherence has thwarted efforts to eliminate TB. Recent data demonstrate an elevated risk of unfavorable outcomes when patients miss as few as 1 dose in 10.^32^

We also demonstrated that a combination of DOT methods enabled the NYC BTBC to achieve high rates of direct observation. Although electronic DOT was preferred by most patients for the remainder of treatment, 6 of 173 patients (4%) declined electronic DOT in favor of in-person DOT for both crossover periods, and 10 (6%) chose to continue treatment with in-person DOT. Furthermore, a goal of this trial was to assess electronic DOT performance when offered to TB patients from the start of outpatient treatment. Eligibility was not predicated on prior treatment adherence. The trial population was largely similar to the population of TB patients and persons being evaluated for TB. Finally, all 4 DOT methods experienced challenges that interfered with observations. These data command consideration in relation to TB program operations.

### Limitations

This study had several limitations. It was not feasible to mask participants and clinicians to the intervention. The direct effect was that 35 of 173 patients (20%) switched from their assigned DOT method or continued with their previously assigned DOT method into the subsequent crossover period. We controlled the analytic impact of these protocol deviations by assessing the individual dose as the unit of analysis, and represented its characteristics (including the DOT method) in the GLMM analytic approach.

Additionally, some patients were not enrolled because of clinician concerns regarding treatment adherence (eTable 2 in Supplement 2). This exclusion may affect the generalizability of the results. Conceivably, clinicians in settings that offer both in-person and electronic DOT will make similar decisions.

During crossover period 1, more participants in group 2 (24 individuals) withdrew from the study than in group 1 (14), leading to a slightly greater proportion of group 1 participants among the 173 patients included in statistical analysis. However, the effect of this imbalance appeared to have been negligible since bootstrap percentage differences estimated from ITT and EMP analyses of the 206-patient sample (which included 33 of the 38 participants who withdrew), were quantitatively similar to those estimated in modified ITT and EMP analyses with the 173-patient sample. Finally, some patients become less adherent once symptoms abate. This evaluation focused on adherence following the start of outpatient treatment.

Additional study of electronic DOT and logistical challenges surrounding its use across populations with historically sub-optimal treatment outcomes and within different cultural and economic settings is warranted. Insights into how programs may further enhance the effectiveness of electronic DOT are needed. For example, given the social support patients derive from in-person DOT, there may be benefits from supplementing recorded electronic DOT with optimally timed live-video interactions.

## Conclusions

In a randomized, crossover noninferiority trial implemented in an urban TB program with a history of successful in-person DOT practice, we found electronic was as effective as in-person DOT for assuring high levels of TB treatment adherence. The findings from this trial support adoption of electronic DOT as a standard care option for programs successfully using in-person DOT, a practice adopted by the NYC BTBC at the start of the COVID-19 pandemic.

## Notes

Supplement 1.
Study Protocol

Supplement 2. **eAppendix 1.** Statistical Details and Auxiliary Analyses **eAppendix 2.** Noninferiority of eDOT Between Seasons **eTable 1.** Dose Outcome Analysis With Percentage Differences **eTable 2.** Reasons for Study Exclusion or Declined Enrollment Among Persons Who Met Initial Screening Criteria **eTable 3.** Characteristics of Enrolled Participants by Randomization Assignment **eTable 4.** Model-adjusted Percentage Differences Between eDOT vs. ipDOT Estimated With Bootstrap Logistic GLMM, by Season (Quarter) **eFigure.** Dose Outcome Completed

Supplement 3. **Nonauthor Collaborators.** The eDOT Study Team nonauthor collaborators

Supplement 4.
Data Sharing Statement
