# Supplementary material for: In-Person vs Electronic Directly Observed Therapy for Tuberculosis Treatment Adherence: A Randomized Noninferiority Trial
Source: JAMA Netw Open. 2022 Jan 20;5(1):e2144210. doi: 10.1001/jamanetworkopen.2021.44210 (PMC8777548; doi:10.1001/jamanetworkopen.2021.44210)
Supplement: Supplement 1. — Study Protocol [file jamanetwopen-e2144210-s001.pdf]

Burzynski J, Mangan JM, Lam CK, et al; eDOT Study Team. In-person vs electronic directly observed therapy for tuberculosis treatment adherence: a randomized noninferiority trial. *JAMA Netw Open.* 2022;5(1):e2144210. doi:10.1001/jamanetworkopen.2021.44210

TITLE

An evaluation of traditional directly observed therapy (DOT) and electronic forms of DOT for Tuberculosis (TB) treatment

Funding Agencies:

U.S. Centers for Disease Control and Prevention (CDC)

Study Leads:

Joseph Burzynski, MD, MPH

Michelle Macaraig, DrPH, MPH

Joan M. Mangan, PhD, MST

Neela D. Goswami, MD, MPH

Version Number: 9
December 1, 2018

This protocol outlines a U.S.-based, 1 site (with 4 clinical settings), randomized controlled trial (with funding from CDC's Antibiotic Resistance Solutions Initiative) that will be implemented to evaluate traditional directly observed therapy (DOT) and electronic forms of DOT (eDOT) for tuberculosis (TB) treatment. The trial will assess whether eDOT that employs electronic communication methods, such as video via computer or cellphone, is a non-inferior approach to monitor TB treatment adherence, compared to traditional in-person DOT (ipDOT), in which a trained person is in the physical presence of patients as anti-TB drugs are ingested. ipDOT is the single best intervention proven to be successful when it comes to TB patients' adherence to therapy (which reduces risk of acquired drug resistance). However, ipDOT is resource intensive and many times challenging to facilitate in-person. If eDOT is found to be non-inferior to ipDOT, health departments and other clinicians might be able to provide eDOT to certain populations of TB patients in a more flexible and potentially cost-saving manner.

Table of Contents

Protocol Summary 6

Study Schematic 8

1 Key personnel 8

2 Background Information and Scientific Rationale 11

2.1 Background Information 11
2.2 Study Rationale 13
2.3 Potential Harms and Benefits 13
2.3.1 Potential harms 13
2.3.2 Potential benefits 14

3 Description of Study 15

3.1 Phase 1: Assessment of eDOT in relation to treatment adherence, TB case-management, and treatment outcome 15
3.1.1 TB Treatment 16
3.1.2 Assessment of Participant Adherence with TB Treatment 16
3.1.3 Equipment 16
3.2 Phase 2: Patient Perceptions of DOT Methods 17

4 Objectives 19

4.1 Primary: 19
4.2 Secondary: 19

5 Study Design 20

6 Study Population 21

6.1 Inclusion Criteria 21
6.2 Criteria for Exclusion from Enrollment 22
6.3 Criteria for Exclusion after Enrollment ('Late Exclusion') 22

7 Enrollment and Randomization 24

7.1 Enrollment Procedures 24
7.2 Randomization 24

8 Study Procedures 27

8.1 Clinical Evaluations 27
8.1.1 Sputum Smear and Culture Conversion 27
8.2 TB Treatment Regimens 27
8.3 Identification of Potential Participants 27
8.4 Study Intervention 28
8.5 Measuring Medication Doses 28
8.5.1 Proportion of Medication Doses Directly Observed 28
8.5.2 Patient Adherence to Scheduled DOT Doses 30

8.5.3 Documentation of Medications in the NYC DOHMH BTBC EMR and Study Database 33
8.6 Assessments of Medication Side Effects 37
8.7 Management of Participants who are Discontinued from Treatment Due to Medication Side Effects 37
8.8 Clinician or Bureau of TB Control Staff at Study Site Judges that eDOT or study discontinuation is in the Participant's Best Interest 37
8.9 Management of a Participant who chooses to Withdraw from the Study 37
8.10 Management of a Participant who is incarcerated after Enrollment 37
8.11 Loss to Follow-up 38
8.12 Premature Termination of the Study or Closure of a Study Phase or a Study Site 38
8.13 Incentives 38
9 Study Schedule 39
9.1 Screening 39
9.2 Enrollment and Baseline Visit 40
9.2.1 Demographic and Contact Information 41
9.2.2 Medical and Social History 41
9.2.3 Self-efficacy to Adhere to Treatment 42
9.2.4 eDOT video type 42
9.2.5 Randomization 42
9.2.6 eDOT Device 42
9.3 Treatment Observation: Cross-over Period 43
9.4 Cross-over Part 1 Completion Visit 43
9.5 Cross-over Part 2 Completion Visit 44
9.6 Treatment Observation – TB Treatment Continuation Period 44
9.7 Documentation of Sputum Culture Conversion 45
9.8 Documentation of Treatment Outcomes 45
9.9 Documentation of Medication Side Effects 45
9.10 Focus Groups with Study Participants 46
9.11 Early Termination / Study Withdrawal 47
9.12 Study Termination / Treatment Completion 47
10 Assessment of Safety 48
10.1 Overview 48
10.2 Specification of Measures 49
10.2.1 Primary Outcome Measure 49
10.2.2 Secondary Outcome Measure 49
10.3 Methods and Timing for Assessing Medication Side Effects 49
10.4 Recording and Reporting Procedures 49
10.5 Follow-up of Participants Following Medication Side Effects 50
11 Statistical Considerations 51
11.1 Study Hypotheses 51
11.2 Study Outcome Measures 51
11.3 Analysis Groups 52

11.4 Analysis Plan 53
11.4.1 Primary analyses 53
11.4.2 Secondary analysis 54
11.4.3 Analysis of Patients Perceptions of eDOT 54
11.4.4 Data Agreements and Confidentiality Forms 54
11.5 Sample Size Considerations 55
12 Quality Control and Quality Assurance 58
12.1 Data Quality Management 58
12.2 External monitoring 59
12.3 Study Data Sources 59
13 Ethics/Protection of Human Subjects 61
13.1 Institutional Review Board 61
13.2 Informed Consent Process – Non-inferiority Study 61
13.3 Informed Consent – Focus Groups 62
13.4 Subject Confidentiality 63
13.5 Study Discontinuation 64
14 Data Handling and Record Keeping 65
15 Roles and responsibilities of study team 66
15.1 Study Sponsor: Division of Tuberculosis Elimination, National Center for HIV/AIDS, Viral Hepatitis, STD, and TB Prevention, U.S. Centers for Disease Control and Prevention 66
15.2 NYC DOHMH BTBC Research Team 66
15.2.1 Principal Investigators 66
15.2.2 Study Coordinator 67
15.2.3 Data Analyst 68
15.2.4 Study Facilitators 69
16 Timeline 71
17 Publications and Dissemination of Study Results 74
18 Literature References 75

APPENDICES

A: Schedule of Procedures/Evaluations

B: Figure 1: Illustration of Screening, Enrollment, Study-related Activities

C: Abbreviations

D: List of Forms and Materials

E: Consort Flow Diagram (blank)

F: Participant Consent and Assent Forms and Authorization for Use/Disclosure of Protected Health Information for Research

Title: An evaluation of traditional directly observed therapy (DOT) and electronic forms of DOT for tuberculosis (TB) treatment

Hypothesis: Directly observed therapy (DOT) that employs electronic communication methods (eDOT) is a non-inferior approach to monitor treatment adherence, compared to traditional forms of DOT, in which a trained person is in the physical presence of patients as anti-TB drugs are ingested (ipDOT).

Design: This will be a U.S.-based, 1 site (with 4 clinic settings), randomized, cross-over, 2-arm, non-inferiority trial with randomization to either traditional in-person DOT (ipDOT) or electronic DOT (eDOT)*, at the time outpatient treatment begins within participating health department clinics.

*****Secondary analyses will evaluate DOT conducted in “real time” or “live” (eDOT-live) compared to DOT that uses a recorded video (eDOT-recorded).

Population: Patients newly diagnosed with drug-sensitive or non-rifamycin resistant TB.

Site: Four clinics of the New York City Department of Health and Mental Hygiene, Bureau of Tuberculosis Control.

Study Duration: Duration per participant is approximately 6 months.

Description of Intervention:

After providing written informed consent, participants will be randomly assigned to one of the following DOT study group assignments: (1) traditional in-person DOT (ipDOT) or (2) electronic DOT (eDOT).

NOTE: Patients and their providers will discuss and choose the type of eDOT they will use. The two options are: (2a) eDOT conducted “live” in which TB program staff interact with patients in real-time via a computer or phone application as they ingest their medication (eDOT-live), and (2b) eDOT in which patients record themselves ingesting their TB medication using “time-stamped, recorded” videos for TB program staff to review within 1 business day (24 hours), and verify that patients ingested their medication doses as scheduled (eDOT-recorded).

Following 20 observable medication doses under an initial DOT study group assignment participants will be assigned (crossed-over) to the opposite DOT method to collect data on another 20 observable medication doses. Specifically, participants who initially received ipDOT will switch to eDOT. Participants initially assigned to eDOT will switch to ipDOT.

At the conclusion of this Cross-Over Period with 40 observable medication doses, participants will continue treatment using their preferred DOT method.

Objectives:

Primary:

- To compare the proportion of medication doses that are directly observed by ipDOT versus eDOT at the conclusion of the Cross-Over Period.

Secondary:

- To compare the proportion of medication doses not directly observed by ipDOT versus eDOT at the conclusion of the Cross-Over Period due to: equipment malfunction or loss, staff unavailability, patient travel/ work/ school, inclement weather, or other reasons.
- To compare the proportion of medication doses directly observed and not directly observed by ipDOT versus eDOT from the conclusion of the Cross-Over Period until the completion of treatment (continuation period).
- To compare patient adherence to scheduled DOT sessions for ipDOT, eDOT-live, and eDOT-recorded during both the cross-over and continuation periods.
- To compare patient characteristics associated with adherence across ipDOT, eDOT-live, eDOT-recorded.
- To compare the type, frequency, and time between initial symptoms of medication side effects and discussion with a medical provider across DOT methods. The decision to discontinue eDOT will be assessed, as well.
- To compare the proportion of patients with culture confirmed pulmonary TB who achieve sputum conversion within 60 days of treatment initiation, by DOT method.
- To compare the proportion of participants completing treatment to those lost to follow-up or refused further treatment, transfer or move, experience treatment failure, or expire (with death attributable to tuberculosis) across eDOT and ipDOT.
- To examine participants' preferred DOT method following the Cross-Over Period in the context of patient demographics, self-efficacy to adhere to treatment, and treatment completion.
- To assess patient perceptions of quality of care, overall satisfaction with patient-staff relationships/rapport, and self-efficacy to adhere across eDOT and ipDOT.

Figure 1. Schematic of Study Design:

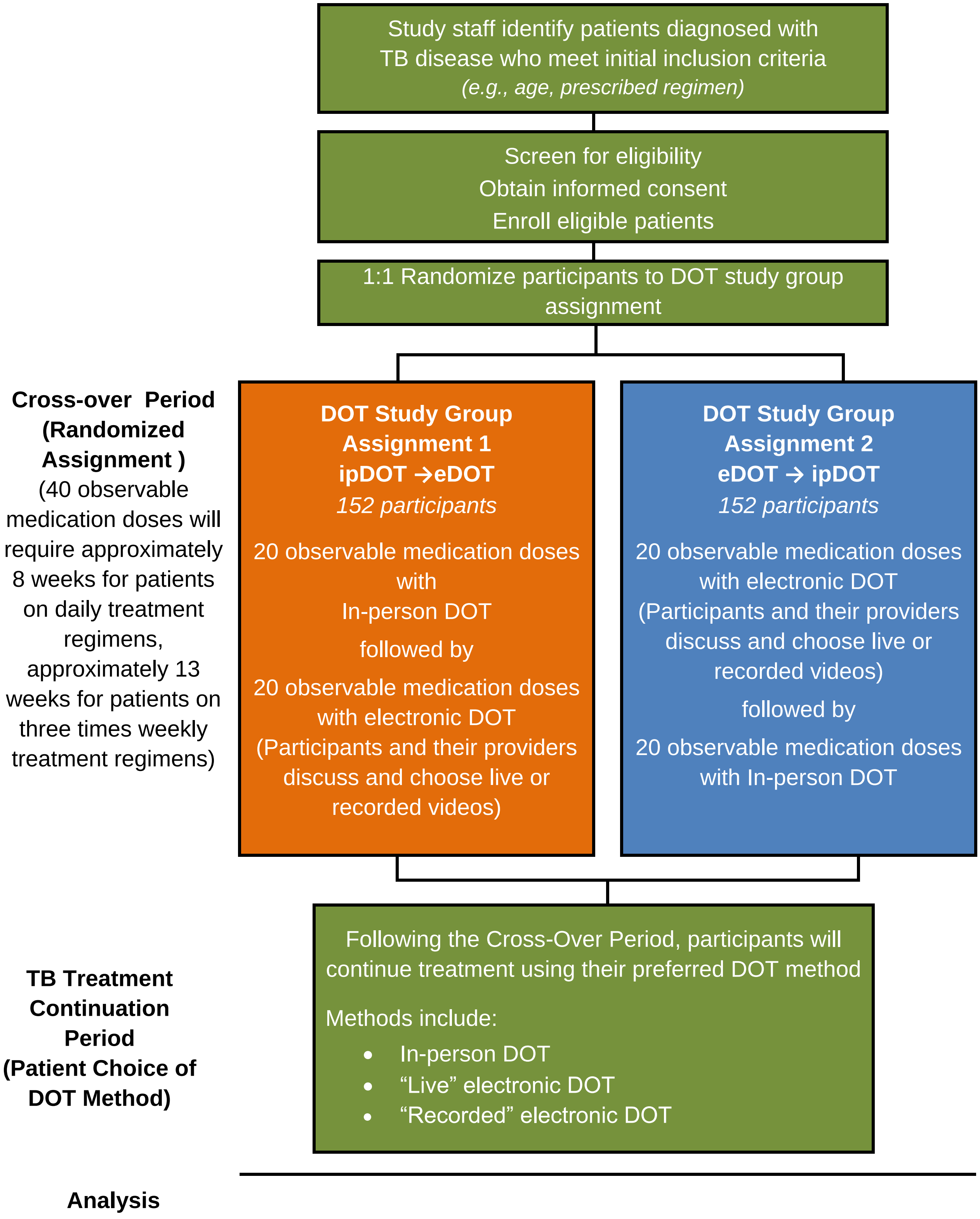

1 KEY PERSONNEL

Funding Agencies:
U.S. Centers for Disease Control and Prevention

Study Leads:
Joseph Burzynski, MD, MPH
Michelle Macaraig, DrPH, MPH
Joan M. Mangan, PhD, MST
Neela D. Goswami, MD, MPH

Key Personnel:

Name	Institution	
Sapna Bamrah Morris, MD	Medical Officer<br>CDC Division of Tuberculosis Elimination<br>Tel: 404.639.8289<br>Email: SBMorris@cdc.gov	
Joseph Burzynski, MD, MPH<br>**DOHMH Principal Investigator**	Assistant Commissioner<br>New York City Department of Health & Mental Hygiene<br>Bureau of Tuberculosis Control<br>Tel: 347.396.7511<br>Email: jburzyns@health.nyc.gov<br>http://www.nyc.gov/html/doh/html/diseases/tb.shtml	
Christine Chuck, MPA	New York City Department of Health & Mental Hygiene<br>Bureau of Tuberculosis Control<br>Tel: 347.396.7480<br>Email: Cchuck@health.nyc.gov	
B. Rey de Castro, ScD	Statistician<br>CDC Division of Tuberculosis Elimination	Tel: 770.488.0162<br>Email: jsq7@cdc.gov
Richard S. Garfein, PhD, MPH<br>**CONSULTANT**	Professor<br>Division of Global Public Health<br>Department of Medicine<br>Univ. of California San Diego<br>Tel: 858.822.3018<br>Email: rgarfein@ucsd.edu	Sure Adhere Mobile<br>Tel: 858.220.3917<br>Email:<br>Richard.Garfein@sureadhere.com
Andrew Hill, PhD<br>**CONSULTANT**	Statistician<br>CDC Division of Tuberculosis Elimination<br>Tel: 404.639.4165<br>Email: fyu7@cdc.gov	

Neela D. Goswami, MD, MPH	Medical Officer<br>CDC Division of Tuberculosis Elimination<br>Tel: 404.718.5614<br>Email: nef7@cdc.gov	
Chee Kin Lam<br>**DOHMH Study Coordinator**	New York City Department of Health & Mental Hygiene<br>Bureau of Tuberculosis Control<br>Tel: 347-396-7496<br>Email: clam4@health.nyc.gov	CDC Division of Tuberculosis Elimination<br>Email: Xko9@cdc.gov
Carol Yen-Chin Lin, PhD	Statistician<br>CDC Division of Tuberculosis Elimination	Tel: 404.639.6427<br>Email: cvl6@cdc.gov
Michelle Macaraig, DrPH<br>**DOHMH Co-Principal Investigator**	New York City Department of Health & Mental Hygiene<br>Bureau of Tuberculosis Control<br>Tel: 347-396-7536<br>Email: mmacarai@health.nyc.gov	
Joan M. Mangan, PhD, MST	Senior Behavioral Scientist<br>CDC Division of Tuberculosis Elimination<br>Tel: 404.639.8987<br>Email: bpy4@cdc.gov	
Diana M. Nilsen, MD, RN	Director of Medical Affairs<br>New York City Department of Health & Mental Hygiene<br>Bureau of TB Control<br>Tel: 347.396.7486<br>Email: dnilsen@health.nyc.gov	
Margaret Oxtoby, MD<br>**CONSULTANT**	Bureau of Tuberculosis Control<br>New York State Department of Health<br>Corning Tower, Room 565<br>Albany, NY<br>Tel: 518.474.7000	CDC Division of Tuberculosis Elimination<br>Field Services Branch<br>Email: MOxtoby@cdc.gov
Errol Robinson, MPA, BSEE	Clinical Operations Director<br>New York City Department of Health & Mental Hygiene<br>Bureau of TB Control<br>Tel: 347.396.7493<br>Email: erobinso@health.nyc.gov	
Neil Schluger, MD	Chief, Division of Pulmonary, Allergy and Critical Care Medicine<br>Professor of Medicine, Epidemiology and Environmental Health Sciences<br>Columbia University<br>Tel: 212.305.4904 / 212.305.9817<br>Email:ns311@columbia.edu	
Brock Stewart, PhD	Statistician	

	CDC Global Immunization Division<br>Tel: 404.639.0215<br>Email: jnn6@cdc.gov
Andrew Vernon, MD<br>**CONSULTANT**	Chief, Clinical Research Branch<br>CDC Division of Tuberculosis Elimination<br>Tel: 404.639.5341<br>Email: AVernon@cdc.gov
Marco Salerno, MPH	Data Analyst
Michael Reeves, MS	Study Facilitator
Sarah Kiskadden-Bechtel, MS	Study Facilitator
Charlene Sathi	Study Facilitator
Sheridan Bowers	Study Facilitator

2 BACKGROUND INFORMATION AND SCIENTIFIC RATIONALE

2.1 Background Information

Tuberculosis is a serious threat level pathogen

Tuberculosis (TB) is among the most common infectious diseases and cause of death worldwide. The bacteria that causes TB, *Mycobacterium tuberculosis* (*Mtb*), is spread when a person with TB disease of the lungs or throat coughs, speaks, or sings. These bacteria can float in the air for several hours, depending on the environment. Persons who breathe in the air containing these TB bacteria can become infected.

The bacteria can deftly evolve and become resistant to anti-TB drugs when these drugs are misused or mismanaged. Examples include when patients do not complete their full course of treatment; when health-care providers prescribe the wrong treatment, the wrong dose, or length of time for taking the drugs; when the supply of drugs is not always available; when patients experience poor drug absorption or drug interactions; or when the drugs are of poor quality.

The World Health Organization (WHO) estimates that 9.6 million became ill with TB in 2014. Among this group, approximately 480,000 persons became ill with multidrug-resistant TB (MDR TB), which is TB caused by bacteria that are resistant to at least isoniazid and rifampin, the two most potent TB drugs used to treat persons with TB disease. Extensively drug resistant (XDR) strains of TB were reported by 105 countries in 2015. As such, the National Strategy for Combatting Antibiotic Resistant Bacteria (CARB) has designated *Mtb* a SERIOUS threat level pathogen.

Multidrug-Resistant Tuberculosis in the United States

In the U.S. in 2014, of 9,421 reported TB cases, it was estimated that 1-2% of these cases were MDR TB with direct costs for treatment averaging $134,000 per case (in 2010 dollars). [1] CDC funds health departments in all 50 states, 10 large cities, DC, Puerto Rico, Guam, the Virgin Islands, and the U.S.-associated Pacific Islands to conduct surveillance, provide laboratory testing, perform contact investigations, diagnose cases, provide directly-observed therapy and medical management for TB cases, and therapy for latent TB infection. Five TB Regional Training and Medical Consultation Centers (RTMCCs) provide training and medical consultation for these programs. These programs all confront common challenges that include the need to prevent the creation and augmentation of resistance to available anti-TB medications.

Preventing acquired drug resistance through directly observed therapy (DOT)

Completion of treatment by persons with TB disease represents the optimal path to the program goals of prevention of morbidity and mortality, cure of the patient, interruption of transmission,

and prevention of acquired drug resistance. The single best intervention in this regard has proven to be DOT.

Moreover, DOT provides more frequent interactions between the patient and the patient's healthcare team. This enables better monitoring and efficient response to medication side effects. This is especially important as medication side effects are among the top reasons patients are lost to follow-up during treatment therapy. [2-5]

Experience in the U.S. in the 1990s demonstrated the efficacy of this intervention in the prevention and control of drug-resistant tuberculosis.[6-11] Studies in the past 15 years in international settings have challenged the utility of DOT, but have been criticized for imperfect to poor design or implementation.[12-13]

Approaches to DOT

DOT entails a trained "observer" acceptable to both the patient and the health system being present to monitor treatment adherence as patients swallow anti-TB drugs. Traditionally, TB programs have engaged a range of people, including nurses, community health workers, family members, and former TB patients to serve as treatment monitors; and have provided DOT in a variety of settings, including health facilities, pharmacies, patient's homes, and the homes of community volunteers.

In the United States, where DOT remains a cornerstone of TB control, TB programs aim to deliver medications daily or intermittently to patients in their homes or a location convenient to the patient. However, this approach is not without some inconvenience to patients. Meetings with healthcare providers are not always convenient as they take time each day from a patient's routine, requiring patients to be in one location at a specific time each day and to wait at this location in the event the healthcare staff is behind schedule. DOT can also be somewhat inflexible, as it may better suit the staff members' schedule, interfere with the patient's employment, and be a source of stigma for a patient as community members observe staff members' daily arrival and departure. [14-15]

While DOT represents the treatment standard, the implementation of DOT has been modified by some programs (e.g., DOT is reserved for high-risk patients only, or patients self-administer a portion of doses each week) in an effort to reduce costs and conserve program resources. In the U.S., efforts recently have sought to utilize advances in communication technology to facilitate the implementation of DOT. Cell phone or computer-based video DOT has been preliminarily investigated in several program settings, including San Diego and New York City. [16-17]

Currently, the New York City Department of Health and Mental Hygiene (NYC DOHMH) offers "live video" eDOT to patients who are eligible for DOT, 12 years of age or older, speak English Spanish, and a selection of other languages, have a private space where they can conduct eDOT, are proficient in using a smart-phone with video conferencing capability (See Section 9.2.4 eDOT Device), and are able to identify and administer to oneself the prescribed

medications. Prior to beginning eDOT, most patients are observed for 2 weeks of treatment in the clinic. Once the patient is determined to be eligible for eDOT, the eDOT worker and patient pre-arrange a schedule for the eDOT calls. The eDOT worker receives calls using a webcam-equipped computer. Patients are asked about side effects, and if none are reported, the patient shows and names each pill in front of the camera before swallowing it. To demonstrate that the pills have been swallowed, the patients open their mouths in front of the camera and engage in conversation with the eDOT worker for several minutes. If any side effects are reported, a NYC DOHMH physician is connected by video or audio to provide medical advice. If a physician is unavailable, the patient is referred to the TB clinic to be evaluated in-person. Each eDOT session is documented in the NYC DOHMH electronic medical record (EMR) system. Technical or operational issues are directed to supervisors for resolution and tracked in a separate database. Missed eDOT appointments are followed up by phone calls and, if these are unsuccessful, home visits. [17]

2.2 Study Rationale

This study will evaluate traditional approaches to DOT compared to DOT by electronic methods. The study will be based within, and primarily conducted by the NYC DOHMH, Bureau of Tuberculosis Control (BTBC) clinics. This will enable the study to be to be conducted in a programmatic setting and reflect "real-life" situations.

2.3 Potential Harms and Benefits

2.3.1 Potential harms

Risks associated with study participation include the possibilities that the efficacy of electronic approaches to DOT is inferior to the traditional approach in which an observer is present while a patient ingests his/her medication. There is also the possibility for the loss of confidentiality.

Efficacy of electronic approaches to DOT

DOT provided by electronic methods may prove to have inferior efficacy compared to traditional methods of DOT with respect to: (1) treatment adherence; (2) the intensity of the supervision provided (i.e., eDOT may be conducted in real time or in a "recorded" manner); (3) timely delivery of care for medication side effects or lessen the severity of medication side effects; and (4) patients' perceptions of the care and support received.

Studies conducted within the United States and Mexico have demonstrated that eDOT is acceptable and improves patient commitment to treatment, even in highly mobile populations.[18] This early success has led programs within the United States, England, India, Moldova, and Belarus to begin implementing eDOT programs. However, the technology remains relatively new and knowledge of its effectiveness and drawbacks is limited. [19]

Confidentiality

DOT provided by electronic methods presents risks to patient confidentiality while data are transferred; however, these issues will be addressed through encryption, Health Insurance Portability and Accountability (HIPAA) compliant software, and secure data management, reviewed and approved by the NYC DOHMH Information Technology Department. Additionally, the risk of disclosure of a TB diagnosis via electronic methods needs to be balanced against the likelihood for the same to happen when patients visit TB clinics regularly or an ipDOT observer visits them each day. [19] Confidentiality risks will be minimized through measures described in Sections 7, 13, 14, and 15.

2.3.2 Potential benefits

Study participants may benefit by experiencing both methods of DOT, and then having the opportunity to choose their preferred method of DOT at the end of the Cross-Over Period, for the duration of their treatment.

Study participants will benefit indirectly, as the study will provide supplemental resources for closer monitoring of clinical outcomes and more intensive patient follow-up than might otherwise be available for patients receiving routine care.

This study will benefit TB programs across the U.S. and society by contributing to the understanding of optimal strategies for treating TB.

3 DESCRIPTION OF STUDY

This study will be conducted in the phases outlined below, in order to meet our objectives and test our hypotheses.

3.1 Phase 1: Assessment of eDOT in relation to treatment adherence, TB case-management, and treatment outcome

On a daily basis, study coordinators and facilitators will communicate with case managers and clinic staff, and review clinic management systems to identify new TB patients and determine if these individuals meet initial inclusion criteria (i.e., age) and are not (a) serving a prison sentence or residing in an institutional setting, (b) undergoing deportation proceedings, or (c) suspected to have rifampin-resistant TB. Study facilitators will flag these individuals' charts or notify the provider that the individual may qualify for the study. The provider will assess these individuals for any physical or cognitive challenges that would preclude them from being enrolled in the study.

If the provider determines the patient is eligible, he/she will introduce the topic of the study to the patient. If the patient is amenable to further discussions, a study facilitator or the study coordinator will approach the patient to discuss the study. If the patient is amenable to enrolling in the study, staff will proceed with obtaining the individual's written informed consent and authorization for use/disclosure of protected health information for research. Study participants will be randomly assigned to one of the following methods of DOT: (a) traditional in-person DOT (ipDOT), or (b) eDOT. Those assigned to eDOT will discuss and choose an eDOT video type with their provider. The two video options are: DOT conducted "live" in which TB program staff interact in "real time" with patients via a computer or phone application as they ingest their medication (eDOT-live); and electronic DOT in which patients record and submit videos of themselves taking their medicine (eDOT-recorded). TB program staff log into an electronic system to review recorded videos within 1 business day, in order to verify that patients ingested their medication doses as scheduled. Study staff will educate and make scheduling arrangements as appropriate, based upon the participant's initial DOT study group assignment. For those on eDOT, staff will ensure the patient either agrees to use his/her personal phone for the eDOT sessions or loan him/her a DOHMH phone. Patients will be trained on how to download the video application (if they are using their own phone), and how to use the application.

After participants have completed 20 observable medication doses of prescribed treatment under their initial DOT study group assignment , participants will 'cross-over' and be assigned to the alternate method for the next 20 doses. Specifically, participants who initially received traditional ipDOT will switch to eDOT and discuss and choose a video type with their provider. Participants initially assigned to eDOT will switch to traditional ipDOT.

At the conclusion of the Cross-Over Period, participants will undergo the remaining treatment with DOT delivered according to each participant's preferred method.

As part of Phase 1, data will be collected related to: DOT doses taken as intended or missed, total doses delivered via DOT and Self-Administered Therapy (SAT), sputum culture conversion, medication side effects, time to treatment completion, and treatment outcomes.

Data will also be collected specific to each participant's treatment regimen, demographic characteristics (e.g., age), physical challenges (e.g., vision, hearing, dexterity difficulties, literacy), primary language spoken, clinical factors (e.g., results of sputum culture for mycobacteria), social history, location of in-person DOT, timing of ipDOT and eDOT (study group assignment), eDOT video type (live or recorded videos), and the program's use of enablers or incentives for adherence.

3.1.1 TB Treatment

Participants will be treated with regimens active against drug-sensitive or non-rifamycin resistant TB. Treatment will be prescribed and supplied to patients according to the policies of the local TB program.

Details regarding assessment of treatment adherence is provided in Section 9.3 Treatment Observation: Crossover Period and Section 9.6 Treatment Observation: TB Treatment Continuation Period.

3.1.2 Assessment of Participant Adherence with TB Treatment

As described in sections 3.1 and 8.5, each dose of anti-TB medication and method of DOT will be documented by study staff using program records and the study specific database.

Non-adherence will prompt an investigation into the reason for non-adherence, and measures will be taken to address the non-adherence in accordance with program policies and procedures. Study staff will document reasons for patients' non-adherence to their prescribed treatment.

3.1.3 Equipment

Equipment includes any video enabled device that is compatible with eDOT software; this includes smart-phones, tablets, mini-tablet, and computers – also known as "personal devices."

As per current practice within the NYC DOHMH BTBC, participants will use personal smart phones, tablets, or computers with a SIM card or phone app (small portable computers that accept input directly onto the screen rather than via a keyboard or mouse, and may be used as a phone with the addition of a SIM card or via a phone app)

to participate in eDOT visits. Participants using personal phones (or other video enabled personal devices) will be provided an incentive equal to $10 a month for each month they use their devices, during the Cross-Over Period and Treatment Continuation Period, to reimburse for data usage while participating in this study. Patients will download the free video application in their device.

Participants who do not own or have a personal device compatible with the DOT video applications will be provided a smart phone through the study. Participants will be asked to sign a Phone Use Agreement (PUA) indicating that they will return the smart phone to study staff when their participation in eDOT is completed. The PUA will also be used to document participants' understanding that the phones are to be used primarily for eDOT purposes, communication with NYC DOHMH BTBC, or in the event they require emergency medical services and need to call 911. Patients may use the phones to make personal phone calls to numbers within the United States, but the use of phones for personal use will be limited by terms of the data usage plan. These phones will have the eDOT phone application previously uploaded and the ability to dial the NYC DOHMH BTBC offices and 911. Phones that are returned by participants early in the study will be cleaned of data and will be offered to participants who enroll later in the study.

As noted in section 9.2.4, the use of a personal or loaned device, and type of personal device will be documented in each participant's study record. These data will be reported to provide insight related to logistics and feasibility for TB programs that are considering implementation of an eDOT program.

3.2 Phase 2: Patient Perceptions of DOT Methods

To assess patient perceptions of (1) the quality of care, (2) patient-TB program staff relationships/rapport, (3) the shared decision making process regarding eDOT video type, and (4) overall satisfaction while undergoing treatment via eDOT-live, eDOT-recorded, and ipDOT, participants will be asked to complete a questionnaire (self-administered, or completed with the assistance of a study facilitator) that contains items addressing these factors. The questionnaire will be administered at the conclusion of the Cross-Over Period. The data will be used to provide context to the quantitative data collected through Phase 1.

To gather more in-depth information regarding patient perceptions of the quality of care received, rapport with TB program staff, shared decision making, and satisfaction with the various methods of DOT, a convenience sample comprised of 10-20 participants will be invited to participate in focus group discussions. These focus groups are described in greater detail in Section 9.10 Focus Groups with Study Participants.

Finally, all participants will be asked to complete a questionnaire to assess their self-efficacy to adhere to treatment. Self-efficacy is defined as the "conviction that one can successfully execute the behavior required to produce intended outcomes." [12] The presence of self-efficacy is associated with successful completion of recommended

health actions, while a lack of self-efficacy has been associated with failure to accomplish such actions.

Participants will be asked to complete the self-efficacy questionnaire, during the baseline visit, prior to beginning the Cross-Over Period. The data will be examined in relation to the method of DOT patients use during treatment and in relation to treatment outcomes.

4 OBJECTIVES

4.1 Primary:

- To compare the proportion of medication doses that are directly observed by ipDOT versus eDOT at the conclusion of the Cross-Over Period.

4.2 Secondary:

- To compare the proportion of medication doses not directly observed by ipDOT versus eDOT (both live and recorded) at the conclusion of the Cross-Over Period due to: equipment malfunction or loss, staff unavailability, patient travel/ work/ school, inclement weather, or other reasons.

- To compare the proportion of medication doses directly observed and not directly observed by ipDOT versus eDOT (both live and recorded) from the conclusion of the Cross-Over Period until the completion of treatment (continuation period).

- To compare patient adherence to scheduled DOT sessions for ipDOT, eDOT-live, and eDOT-recorded during both the cross-over and continuation periods.

- To compare patient characteristics associated with adherence across ipDOT, eDOT-live, eDOT-recorded.

- To compare the type, frequency, and time between initial symptoms of medication side effects and discussion with a medical provider across DOT methods. The decision to discontinue eDOT will be assessed, as well.

- To compare the proportion of patients with culture confirmed pulmonary TB who achieve sputum conversion within 60 days of treatment initiation, by DOT method.

- To compare the proportion of participants completing treatment to those lost to follow-up or refused further treatment, transfer or move, experience treatment failure, or expire (with death attributable to TB) across eDOT and ipDOT.

- To examine participants' preferred DOT method following the Cross-Over Period in the context of patient demographics, self-efficacy to adhere to treatment, and treatment completion.

- To assess patient perceptions of quality of care, overall satisfaction with patient-staff relationships/rapport, and self-efficacy to adhere across eDOT and ipDOT.

5 STUDY DESIGN

This will be a U.S.-based, 1 site (with 4 clinical settings), randomized, cross-over, 2-arm, non-inferiority trial with 1:1 randomization at the time treatment begins within one of the 4 participating health department clinics. The two arms are as follows: (1) traditional in-person DOT (ipDOT), (2) electronic DOT (eDOT)

The eDOT group will be segmented into 2 groups: (2a) eDOT conducted "live" in which TB program staff interact with patients via a computer or phone application as they ingest their medication (eDOT-live), and (2b) electronic DOT conducted using "time stamped, recorded" videos in which TB program staff log into a secure cloud server and review videos recorded by patients in order to verify that patients ingested their medication doses as scheduled (eDOT-recorded).

Secondary data analyses will be conducted to compare eDOT-live to eDOT-recorded on all outcomes of interest.

6 STUDY POPULATION

This study will be conducted through 4 clinics of the NYC DOHMH BTBC. Male and female participants who are age 12 or older, with a culture-confirmed or clinical diagnosis of TB will be enrolled in the study.

Target enrollment is: **304** participants.

Pregnant or breast-feeding women will be eligible for inclusion in this study if they initiate TB treatment at a participating site. The sex, ethnicity, and socioeconomic background of study participants are expected to mirror those of the populations served by local TB clinics and the populations most affected by TB worldwide.

Co-enrollment in other clinical trials is permitted if the study does not interfere with a participant's ability to participate in eDOT and fulfill this study's requirements.

6.1 Inclusion Criteria

Individuals must meet all of the following inclusion criteria in order to participate in this study:

1) All TB patients (both those with a confirmed diagnosis and those with a clinical diagnosis), started on treatment for non-rifamycin resistant TB, and eligible to receive DOT.
2) Physician determines the patient may be treated with any treatment regimen for non-rifamycin resistant TB approved by the NYC DOHMH TB program.
3) Individuals found to have INH resistant disease are eligible for inclusion.
4) Age $\geq$18 years or older
5) Age 12 to 17 years, with the consent of a parent or legal guardian
6) An address or residence location that is readily accessible for visiting, and willingness to inform the study team of any change of address during the treatment and follow-up period.
7) No plans to move out of the catchment areas of the participating TB program sites within 9 months of enrollment.
8) Willingness to comply with study procedures and provide written informed consent prior to study enrollment.
9) Individuals for whom a diagnosis of TB has been made clinically are eligible for study inclusion. Data may be collected from these patients related to all objectives with the exception of culture conversion.

6.2 Criteria for Exclusion from Enrollment

An individual meeting any of the following exclusion criteria at the time of enrollment will be excluded from study participation:

1) At the time of enrollment, the patient's *Mtb* isolate is already known to be resistant to rifamycin or prescribed a non-rifamycin treatment regimen.

2) Prescribed any injectable, anti-TB medication as part of an outpatient treatment regimen.

3) Adverse reaction to initial doses of anti-TB medication *(per NYC protocol)* of sufficient severity that in the judgement of the clinician makes study participation not in the individual's best interest.

4) A cognitive or physical disability that prevents full participation in eDOT (e.g., vision, hearing, physically challenged, inability to swallow medications). ***NOTE***: Exceptions will be made for those patients who crush pills in order to swallow the medication, or have a member of their household or a caregiver who can assist them for the duration of the study.

5) Less than 12 years of age.

6) Patients 12-17 years of age, whose parents or legal guardians refuse to provide consent.

7) Incarceration, institutionalization, or other involuntary detention.

8) Plans to move out of the catchment areas of the participating TB program sites in less than 9 months from the day of enrollment.

9) Previously enrolled in this study.

10) Currently enrolled in a clinical trial that prohibits enrollment in another study.

11) Other medical conditions that, in the investigator's or the clinic physician's judgment, make study participation not in the individual's best interest.

6.3 Criteria for Exclusion after Enrollment ('Late Exclusion')

If BTBC clinic staff or study staff determine an enrolled individual no longer meets eligibility criteria, he/she will be withdrawn from the study and the reason for the withdrawal will be documented on the Study Termination / Treatment Completion Form as a late exclusion.

Additionally, microbiological confirmation of drug-susceptible TB is not always expected to be available at the time of enrollment. Enrolled individuals who are subsequently determined to meet either of the following criteria will be classified as 'late exclusions' on the Study Termination / Treatment Completion Form

The patient has not completed 40 observable doses of treatment, AND:

A. *Mtb* cultured or detected through molecular assays (Cepheid Xpert MTB/RIF or Hain MTBDR*plus* assays) from a specimen obtained closest to the time of study entry is determined to be rifamycin-resistant, multidrug-resistant, pre-XDR, or XDR TB.

OR

B. Sputum cultures or cultures from relevant extrapulmonary sites grow nontuberculous mycobacteria and provider discontinues treatment for TB.

OR

C. Clinician rules out TB disease

Note: Data for those participants who have undergone 40 observable doses of treatment prior to laboratory confirmation of rifamycin-resistant, multidrug-resistant, pre-XDR, or XDR TB, a provider rules out TB disease, or a patient’s treatment for TB is discontinued - will be retained, and included in data analysis related to the primary outcome measures and the secondary outcome measures, as appropriate, with the data collected.

7 ENROLLMENT AND RANDOMIZATION

7.1 Enrollment Procedures

Individuals who meet study eligibility criteria will be invited to participate in this study. In accordance with HIPAA and health department confidentiality procedures, NYC DOHMH BTBC program staff will approach prospective participants, explain the purpose of the study, and invite them to speak with study staff regarding study participation (Please See Appendix B – Figure 1: Illustration of screening, enrollment, study-related activities).

If an individual does not wish to speak with study staff this refusal will be documented in the Screening Outcome Section of the Screening Form by study facilitators.

If an individual is interested, study facilitators will then meet with the individual to discuss the study in more detail, including inclusion and exclusion criteria and the risks and potential benefits of all study procedures. If study staff are satisfied that the potential participant understands the information and the potential participant is willing, study staff will then review the informed consent form and ask each individual if he/she would like to participate in the study. If an individual is willing to enroll, they will then be asked to provide written informed consent to participate in the study and authorization for use/disclosure of protected health information for research. Study-specific procedures will be initiated only after an individual has provided written informed consent and authorization for use/disclosure of protected health information for research.

Please see sections 9.1 and 9.2 for more details regarding study-specific procedures for enrollment. See Appendix F for Consent and Assent Forms and the Authorization for Use/Disclosure of Protected Health Information for Research Form.

7.2 Randomization

We will use R statistical computing to generate randomization lists to make DOT study group assignments. This will help to ensure participants are randomized into groups that result in equal sample sizes and minimize the likelihood that assignments will become known or predictable. This randomization approach will be done for each of the 4 participating clinical sites, to further ensure DOT study group assignments are balanced within each site.

Eligible participants (who meet all of the inclusion criteria and none of the exclusion criteria) will be randomly assigned to one of the following two Study group assignments:

DOT Study Group Assignment 1: ipDOT→eDOT: 20 observable medication doses with ipDOT followed by 20 observable medication doses with eDOT

DOT Study Group Assignment 2: eDOT→ipDOT: 20 observable medication doses with eDOT followed by 20 observable medication doses with ipDOT

A Master Randomization List, which will include participant identification numbers and DOT study group assignments for each of the 4 clinical sites will be generated and provided to the NYC DOHMH BTBC Principal Investigator. Thus, all planned DOT study group assignments will be generated a priori.

Information contained in the Master Randomization List will be used to generate a Randomization Envelope Label and a Randomization Assignment Insert, for each potential participant, at each of the 4 clinical sites, which can be opened by the study staff at the appropriate time

- The Randomization Label: The label will include the study name, site name, study PI name, the participant ID number, and additional text fields for hand recording and the date, time and signature of the person opening the envelope.
- Randomization Assignment Insert: The Assignment insert will include the DOT study group assignment as well as all the same information that is on the outside envelope label.

The research coordinator will prepare randomization envelopes *(containing the pre-printed, sequentially numbered label affixed to an envelope and matching, pre-printed DOT study group assignment insert folded into the envelope)* using opaque envelopes, for every potential participant on the Master Randomization List.

Prior to sealing the envelopes, an investigator will complete an audit of the prepared envelopes by comparing 25% of the envelope labels against the corresponding insert and the Randomization Lists. In the event of error is found, every envelope will be audited.

A Master Participant List will be provided for use by study staff at each of the four participating sites. Similar to the Master Randomization List, this list will include participant identification numbers, and space for study staff to record participant names, gender, date of birth, and DOT study group assignments.

When a participant is enrolled in the study, s/he will be assigned a confidential participant identification number by adding his/her name to the very next empty row available on Master Participant List.

The participant will maintain this study ID for the remainder of the study and, thereafter, the Study ID will be used on all research files, case report forms (CRFs), Randomization Lists, Envelopes, and other research documents.

When the participant is ready to be randomized, the study facilitator will pull the Randomization Envelope that matches the Participant Study ID from the sequentially ordered Randomization Envelope file. (Note: Envelopes are not to be drawn out of sequence for any reason other than to ensure members of the same household are randomized to the same DOT study group assignment.)

The study facilitator will then record the requisite information on the Master Participant List and complete the blank fields on the Envelope Label and, finally, open the envelope to review the insert and ascertain the participant's DOT study group assignment. The empty fields on the Envelope Insert must also be completed.

The DOT study group assignment and other relevant randomization data will be recorded on the Master Participant List and the opened envelope and corresponding insert should be filed in the restricted access study file along with the Master Participant List.

An inspection of envelopes and inserts will be completed routinely by the study coordinator to verify correct DOT study group assignment.

In addition to completing the Master Participant List and Envelope's label and insert, the randomization assignment will be noted on the Enrollment and Baseline Visit Form.

8 STUDY PROCEDURES

8.1 Clinical Evaluations

Clinical evaluations will be performed in accordance with local program policies and procedures.

8.1.1 Sputum Smear and Culture Conversion

Collection of sputum for AFB (acid fast bacilli) smear and culture of *Mtb* will also be performed in accordance with local program policies and procedures. Sputum culture results will be documented as part of this study, as an indirect measure of treatment adherence.

8.2 TB Treatment Regimens

TB treatment regimens will be prescribed for participants in accordance with local program policies and procedures.

8.3 Identification of Potential Participants

Persons diagnosed with TB will be recruited through the following 4 clinics within the NYC DOHMH BTBC: Corona TB Chest Center in Queens, Washington Heights TB Chest Center in Manhattan, Fort Greene TB Chest Center in Brooklyn, and Morrisania TB Chest Center in the Bronx. Persons diagnosed with TB, who are under the medical supervision of a licensed private provider in New York City, and agree to undergo treatment using DOT provided by NYC DOHMH BTBC staff will also be recruited. These participants will be offered enrollment in the study during their standard DOT appointment with NYC DOHMH BTBC.

Potential participants will be identified through a preliminary screening process in which the study coordinator and study facilitators will check the electronic medical record (digital clinic) and surveillance (MAVEN) systems used by the TB Chest Centers and communicate with case managers and TB clinic staff daily in order to identify newly diagnosed TB patients and persons with a suspected diagnosis of TB, and conduct a preliminary screening. Preliminary screenings will determine if persons with a new or suspected diagnosis of TB meet the following inclusion criteria: age, not rifamycin resistant TB, not incarcerated or institutionalized. This preliminary screening will enable study facilitators to notify clinic physicians of potential participants and plan for follow-up meetings to discuss study enrollment.

Please see Section 7 through 9 for additional information regarding participant enrollment and randomization procedures and Appendix B – Figure 1: Illustration of screening, enrollment, study-related activities.

8.4 Study Intervention

As described in Section 2.2 Study Rational and Section 5: Study Design, the study intervention is the use of electronic communication methods to observe patients swallow anti-TB drugs and monitor for medication side effects. This study will use two variations of electronic communication methods, referred to as "electronic directly observed therapy" or "eDOT". This includes: (1) eDOT conducted "live" in which TB program staff interact with patients via a computer or phone application as they ingest their medication (eDOT-live), and (2) eDOT conducted using "time stamped, recorded" videos in which TB program staff log into an electronic system and review videos recorded by patients in order to verify that patients ingested their medication doses as scheduled (eDOT-recorded).

8.5 Measuring Medication Doses

As outlined in the study objectives, this study will assess (1) The proportion of medication doses that are directly observed, and (2) patient adherence to scheduled DOT sessions. Our starting point was the following formulas:

<u>Proportion of Doses Directly Observed</u> <u>Patient Adherence to Scheduled DOT Doses</u>

$$\frac{\sum_{i\in\Omega} x_i}{|\Omega|} \leq \frac{\sum_{i\in\Omega_E} x_i}{|\Omega_E|}$$

For any given patient in either DOT study group assignment, let x_i equal 1 when the ith of 20 doses is directly observed, and 0 when the ith dose is not directly observed.
Also, let $\Omega = \{1, 2, \ldots, 20\}$ be an indexing set that indexes the 20 doses.
$|\Omega|$ means the size of Ω, which is 20.
Let $\Omega_E \subseteq \Omega$ be a subset of Ω that only indexes the non-excused doses, i.e. Ω_E excludes the excused doses.
(The notation $i \in \Omega$ means "*i* in Ω").

The following sub-sections provide additional detail related to both measures.

8.5.1 Proportion of Medication Doses Directly Observed

The proportion of medication doses directly observed will be calculated by dividing the number of doses directly observed via ipDOT or eDOT by observable medication doses. Section 8.5.3, Table 2 specifies doses considered observable medication doses.

The Cross-Over Period of this study is comprised of 2 parts, each containing 20 contiguous observable medication doses.

The number of observable medication doses during the TB Treatment Continuation Period will depend on how many doses remain in the treatment regimen following the Cross-over Period for each patient.

The calculation for the proportion of medication doses directly observed during Part 1 of the Cross-over Period is put in plain words below.

$$\textbf{Proportion of Doses Directly Observed} = \frac{\textbf{Number of doses directly observed via ipDOT or eDOT}}{\textbf{20 observable medication doses}}$$

20 Contiguous Doses:

When a patient is prescribed medication 7 days a week, and is expected to self-administer 2 doses during the weekend and take 5 doses in the presence of a TB program or study staff member via ipDOT or eDOT-live 20 contiguous observable medication doses would be completed in 4 weeks.

The time to attain 20 contiguous observable medication doses would extend past 4 weeks if: (1) a holiday occurs and the health department is closed, (2) the patient is on an intermittent treatment schedule, (3) the patient and program has pre-arranged plans for DOT to not be scheduled due to planned vacations, work or school obligations, or (4) other reasons the patient is not scheduled for DOT.

Patients using eDOT-recorded have the capacity to submit 7 videos in a week. All videos submitted will be documented. The doses prescribed Monday through Friday will be used to calculate the proportion of medication doses directly observed, and make comparisons to eDOT-live and ipDOT.

For the purposes of this study, in order to consider a dose of medication to be "observed" a trained health worker must be able to clearly see a patient ingest all pills that comprise the prescribed dose.

Note, if a physician (a) temporarily discontinues a drug in the treatment regimen and/or (b) changes any drug in the treatment regimen, and the patient ingests all medications as prescribed at that point in time, then the dose will be considered "observed."

Also, if a physician stops all prescribed drugs at one time for any clinical reason, then there is no opportunity to observe a dose. In this case, the number of potentially observable medication doses is adjusted based on what a physician has prescribed.

To ensure an accurate calculation of the proportion of medication doses that are directly observed, data from the digital clinic as well as data entered into the study database will identify: (1) when and reasons why patients self-administer doses, (2) when and reasons why DOT is not scheduled, and (3) holidays.

*For additional information please see Section 8.5.3 Documentation of Medications in the NYC DOHMH BTBC EMR

8.5.2 Patient Adherence to Scheduled DOT Doses

Patient adherence to scheduled DOT sessions for ipDOT, eDOT-live, and eDOT-recorded will be assessed per NYC DOHMH BTBC Policy (see "Notes" below).

Patient adherence is calculated by dividing the number of doses directly observed via ipDOT or eDOT by observable medication doses minus any excused absences. Section 8.5.3, Table 2 specified doses considered as observable medication doses.

As noted, the Cross-Over Period of this study is comprised of 2 parts, each containing 20 contiguous observable medication doses. The number of observable medication doses during the TB Treatment Continuation Period will depend on how many doses remain in the treatment regimen following the Cross-over Period for each patient.

The calculation for patient adherence to scheduled DOT doses during Part 1 of the Cross-over Period is put in plain words below.

$$\textbf{Patient Adherence to Scheduled DOT Doses} = \frac{\textbf{Number of doses directly observed via ipDOT or eDOT}}{\textbf{(20 observable medication doses} - \textbf{Number of excused absences)}}$$

Excused Absences:

Program staff may direct a patient to self-administer their medication, giving an "excused absence" from DOT in the event of: (1) inclement weather, (2) public transportation closures/shutdowns, (3) staff unavailability, or (4) other extenuating circumstances.

NOTES:

As outlined in Section 8.5.1, although a provider may prescribe medication to be taken 7 days a week, the NYC DOHMH BTBC's policy is to not count doses taken on the weekends. This includes doses that are taken and documented through eDOT-recorded videos. Thus, only doses taken Monday through Friday are able to be included as an "observable" dose.

Patients who undergo DOT via eDOT-recorded may not be given the same "excused absences" as patients who undergo DOT via ipDOT or eDOT-live. This is feasible because patients have their prescribed medication in their possession and may still record and submit videos during inclement weather or clinic closures.

To ensure an accurate calculation of patient adherence to scheduled DOT doses, data from the digital clinic as well as data entered into the study database will identify: (1) when and reasons why patients self-administer doses, (2) when and reasons why DOT is not scheduled, (3) when and reasons why DOT is not rescheduled, and (4) holidays.

8.5.2.1 Rescheduled Appointments

For the purpose of this study, we will collect data regarding rescheduled appointments and circumstances surrounding rescheduled appointments in order to assess whether or not study participants were adherent to the DOT schedule.

<u>Adherent to DOT Schedule</u>

Patients who are present in person or via the phone app on the appointed day and time will be recorded as adherent to the DOT Schedule.

Per NYC DOHMH BTBC policy, if a patient is late by 15 minutes for a scheduled DOT session, he/she is called to remind them of his/her appointment. If eDOT is conducted successfully using video during this reminder call, the dose will not be rescheduled. Instead the participant is recorded as adherent to the schedule.

<u>Not Adherent to DOT Schedule</u>

Patients who (1) fail to meet with DOT staff for ipDOT, (2) make no contact with the TB program and are not present for an eDOT-live call, or (3) make no contact with the TB program and fail to submit a video for eDOT-recorded will be recorded as non-adherent to the schedule.

If a patient or TB program staff reschedules a DOT visit prior to, or at the time of, a DOT appointment (e.g. due to travel delays, technical difficulties with DOT applications, etc.) adherence to the DOT schedule will be documented as follows:

1. If the DOT appointment cannot be rescheduled because (1) rescheduling would be too close to the next scheduled dose, (2) rescheduling would fall on a "non-observable" day such as a weekend or holiday, or (3) because DOT appointments were missed or rescheduled DOT too many days in the same week - the patient will be instructed to self-administer the medication dose. In this situation the patient will be recorded as not adherent to the schedule and the reasons why the patient was not adherent and why the appointment could not be rescheduled will be documented.

2. If the DOT appointment can be rescheduled, the reason for rescheduling will be documented. Adherence to the schedule will be recorded based on what occurs during the rescheduled appointment.

Figure 2 in Section 8.5.2 illustrates the documentation of medication doses, including rescheduled doses. For additional information please see Section 8.5.3.3 Documentation of Medication – Codes for Schedule Adherence and Issues that May arise during DOT.

8.5.2.2 Figure 2. Documenting Medication Doses

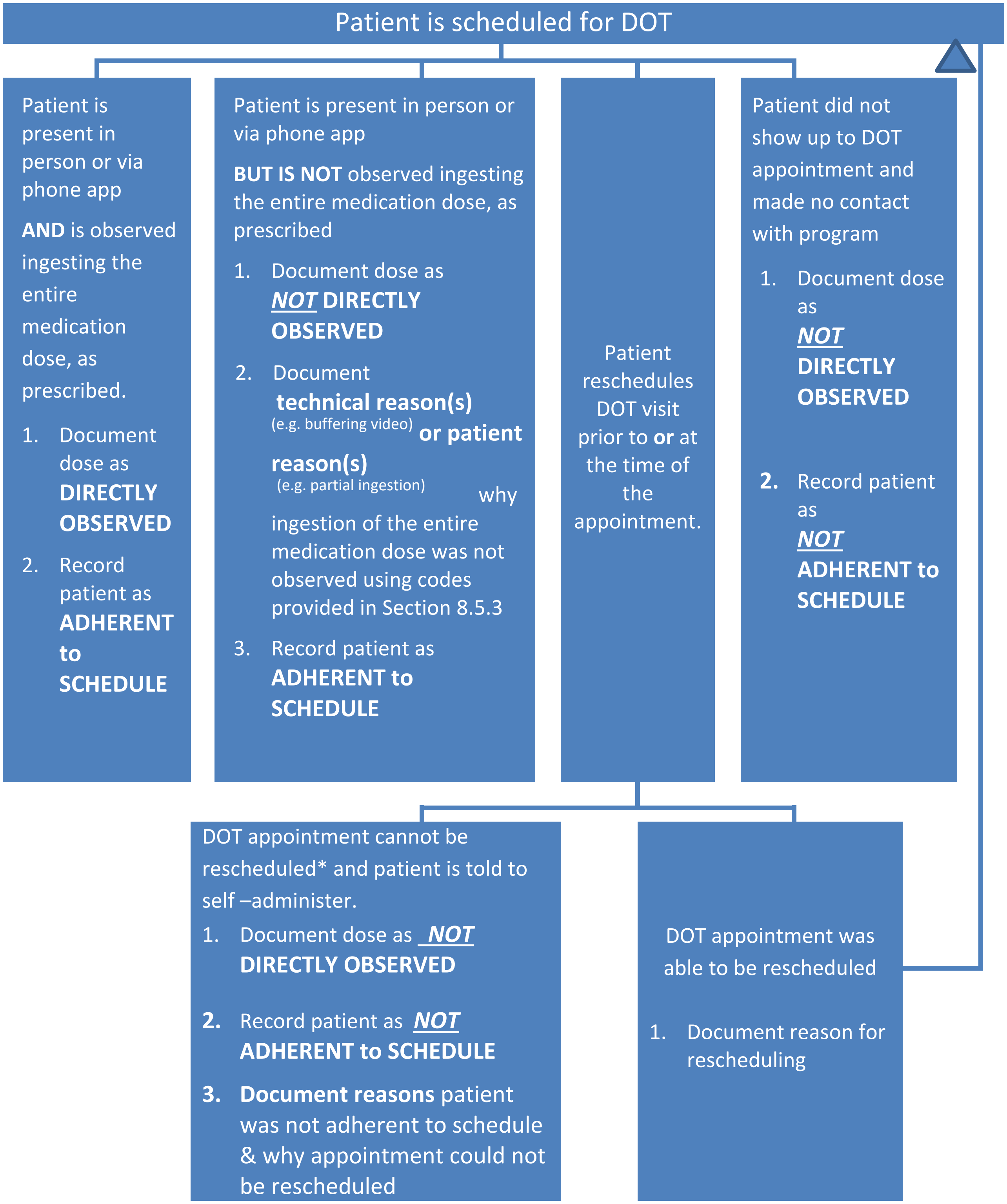

*Reasons why DOT cannot be rescheduled: 1) rescheduling would be too close to the next scheduled dose, 2) rescheduling would fall on a "non-observable" day, e.g. weekend or holiday, 3) patient missed or rescheduled DOT too many days in the same week

8.5.3 Documentation of Medications in the NYC DOHMH BTBC EMR and Study Database

Per NYC DOHMH BTBC policy, each prescribed drug is documented using the codes below in the NYC DOHMH BTBC digital clinic system. These codes will allow investigators to properly calculate the proportion of medication doses directly observed and patient adherence to scheduled DOT sessions.

8.5.3.1 Table 1. NYC DOHMH BTBC Digital Clinic System DOT Codes

Code	Legend	Code	Legend
C	Complete Ingestion	I	Injection
IC	Incarcerated	PD	Physician Decision
NS	Not scheduled	HO	Holiday
D	Died	H	Hospitalized
P	Patient Refused	AD	Admitted to drug rehabilitation program
X	Not a DOT Medication	AR	Adverse Reaction
O	Other Reason	ND	Not at DOT location
M	Medically Approved Absence	NI	No Ingestion Observed
T	Transferred to Long-Term Care Facility	PS	Patient self-administered medication
PV	Partial Ingestion - Vomited	PN	Patient did not show at clinic
PR	Partial Ingestion – Patient refused to take all prescribed medication	PO	Partial Ingestion
S	Stop or Hold*	DC	Discontinued*

*Legend code from paper form – not displayed in legend of digital clinic

Data from the eVERO / digital clinic system will be used to document outcomes of DOT, missed doses, doses that are self-administered and staff notes. Data from the eVERO / digital clinic system will be exported from this system and imported into the Study Specific MS Access database.

Information regarding DOT outcomes (i.e. observed, not observed, and unobservable), adherence to DOT schedule, and issues that arise during eDOT and ipDOT will be documented in the study database. Table 2. DOT Outcomes of Prescribed Medications, outlines when a medication dose is documented as observed, not observed, and unobservable. Table 3 provides codes for issues that may arise during DOT.

8.5.3.2 Table 2. DOT Outcomes of Prescribed Medications

<table>
<tr><th colspan="3">Dose DOT Outcome</th></tr>
<tr><td colspan="2">"Observable Medication Doses"
Medication doses scheduled to be directly observed by health department staff Monday through Friday</td><td>"Unobservable Medication Doses"
Medication doses not scheduled for direct observation by health department staff</td></tr>
<tr><td>Record as "Observed"</td><td>Record as "Not observed"</td><td>Record as "Unobservable"</td></tr>
<tr><td>C = Complete Ingestion
All prescribed medications are marked with a "C"</td><td rowspan="2">PS = Patient self-administered medication
PS is used to document when a patient is scheduled for DOT, but instead self-administers the dose of medication. These doses do count against the patient's adherence rate</td><td>HO = Holiday</td></tr>
<tr><td rowspan="2">PO = Partial Ingestion
If a medication is marked with a "PO" AND the patient's chart or electronic records indicate that the physician has temporarily decreased the prescribed amount of that drug, OR discontinued or stopped the drug</td><td rowspan="2">NS = Not Scheduled
NS is used to document when a patient's regimen requires the patient to take a dose of medication on a day when observations cannot be made (e.g. weekends).

Patients self-administer the medication. These doses do not count against the patient's adherence rates</td></tr>
<tr><td>NI or N = No ingestion observed
If any medication is marked with a "N" the entire dose is documented as not observed (NI or N applies to situations in which staff are unable to see pills or tablets in a patients mouth during eDOT)</td></tr>
<tr><td rowspan="2">DC = Discontinued
If any medication is marked with a "DC" AND all other medications are marked with a "C" for complete ingestion</td><td rowspan="2">PR = Partial Ingestion - Patient Refused to Take All Medication

R = Refused
If any medication is marked with a "PR" or "R" the entire dose is documented as not observed</td><td>M = Medically Approved Absence</td></tr>
<tr><td>PD = Physician Decision</td></tr>
</table>

<table>
<tr>
<td>S = Stop or Hold
If any medication is marked with a "S" AND other medications are marked with a "C" for complete ingestion</td>
<td>PO = Partial Ingestion
P = Partial Ingestion
If a medication is marked with a "P" or "PO" AND the patient's chart or electronic records DO NOT indicate that the physician has temporarily decreased the prescribed amount, discontinued, or stopped the drug</td>
<td rowspan="3">O = Other Reason
H = Hospitalized
IC = Incarcerated
AD = Admitted to Drug Rehab Program

T = Transferred to Long-Term Care
D=Died

If, after study enrollment the patient is noted to be unavailable for any of the reasons above - the doses will be documented as unobservable. The record will note these reasons.</td>
</tr>
<tr>
<td rowspan="2">PV or V = Vomited
If the patient is initially noted to demonstrate complete ingestion ("C") for all drugs, and is subsequently noted to have vomited - the dose will be considered observed</td>
<td>PV or V = Vomited
If the patient begins to ingest medications but stops due to vomiting, the entire dose is documented as not observed</td>
</tr>
<tr>
<td>PN = Patient did not show at clinic

ND = Not at DOT location</td>
</tr>
</table>

8.5.3.3 Table 3. Codes for schedule adherence and issues that may arise during DOT

CODES FOR SCHEDULE ADHERENCE			
**Code**	**Legend**	**Code**	**Legend**
PAS	Patient adherent to schedule	NAD	Not Adherent to Schedule
RSC	DOT visit rescheduled	NRS	DOT visit could not be rescheduled
**CODES FOR ISSUES THAT MAY ARISE DURING eDOT and ipDOT**			
**Code**	**Legend**	**Code**	**Legend**
**Technical Issues**			
SIC	Slow internet connection – causing image freezing or buffering	SMB	Smartphone malfunction – battery not charged
LIT	Low light, poor light – Difficulty seeing patient and/or medications	CBP	Phone camera broken
SMV	Smartphone malfunction – video not working	CSM	Computer or software malfunction
SMA	Smartphone malfunction – audio not working	OTC	Other technical issue
**Patient-Related Issues**			
POV	Patient was out of camera view	PPL	Patient unable to find a private location
PFM	Patient forgot medication at home or other location	PSC	Patient had conflict with work or school schedule
POD	Patient ran out of drug(s) / Patient needs to refill drug(s)	DPU	Exceeded allotted data plan usage
PFA	Patient forgot appointment	PNO	Patient not able to operate smartphone or software application
PRS	Patient DOT appointment rescheduled	OPT	Patient - other problem
MDH	Medical decision medications held	PLF	Patient late more than fifteen minutes
		BRA	Broke Randomization Assignment
**Staff / Environmental Issues**			
SUA	Staff unscheduled absence / illness	INJ	Staff experienced an accident or injury during workday
SEM	Staff needed to respond to an emergency with another patient	TRV	Transportation/ commuting interruptions or delays
WEA	Inclement weather caused safety concerns for travel	OTS	Staff / Program – other problem
LOGip	Logistical delays starting ipDOT	LOGe	Logistical delays starting eDOT
NOI	NO ISSUE		

8.6 Assessments of Medication Side Effects

In accordance with local program policies and procedures, participants will be asked to describe any signs or symptoms of possible medication side effects during DOT sessions and clinic appointments. If a participant does report medication side effects, this information will be documented as part of this study. Please see sections 9.9 and 10.3-4 regarding assessment and documentation of medication side effects.

8.7 Management of Participants who are Discontinued from Treatment Due to Medication Side Effects

Clinicians at a study site may discontinue a participant from TB treatment in the event of severe or serious medication side effects. For study purposes, this should be documented on the Monitoring Medication Side Effects Form and the participant should continue to be followed in the study, unless the participant withdraws consent or treatment was permanently discontinued. Please see Sections 9 and 10 for additional information regarding assessments of medication side effects.

8.8 Clinician or Bureau of TB Control Staff at Study Site Judges that eDOT or study discontinuation is in the Participant's Best Interest

At any time, if a clinician or Bureau of TB Control staff at a study site thinks study or eDOT discontinuation is in the participant's best interest, the study facilitator will document this and notify the study coordinator. For study purposes, a Study Termination / Treatment Completion Form will be completed and reason for eDOT discontinuation will be documented.

8.9 Management of a Participant who chooses to Withdraw from the Study

A participant may choose to withdraw from the study at any time. A Study Termination / Treatment Completion Form should be completed at the time the participant makes his/her choice known. The participant should be able to continue to access all appropriate local sources of care for the management of his/her TB. Participants will be asked to give permission to use the data already collected.

8.10 Management of a Participant who is incarcerated after Enrollment

This study will not enroll prisoners. However, it is possible that a participant will be incarcerated after enrollment. If an enrolled individual is incarcerated, the participant will be treated for active TB according to the standards of the institution in which s/he is incarcerated. While incarcerated, individuals will not be followed in the study. When the individual is no longer incarcerated, study inclusion may continue, at the discretion of the site and clinical coordinator. In the event continued study inclusion is not feasible a Study Termination / Treatment Completion Form should be completed.

8.11 Loss to Follow-up

All efforts should be made to contact participants that miss DOT visits or study visits (unless the participant has withdrawn consent).

For participants who fail to attend a scheduled study visit and attempts to reach the participant by phone are not successful or not feasible, study staff will follow the NYC DOHMH BTBC's return to service procedures. The decision to continue the patient in the study will be at the discretion of the physician.

If a participant misses a cross-over part 1 or part 2 completion visit then repeated efforts must be made to contact the participant, with at least 3 home visits at different times and days of the week. If these attempts are unsuccessful then the participant should be considered lost to follow-up and reported as such on the Study Termination / Treatment Completion Form.

8.12 Premature Termination of the Study or Closure of a Study Phase or a Study Site

The sponsor has the right to close the study and the sponsor has a right to close a site or phase of the study. Study termination, site closure, or phase closure should occur only after consultation between involved parties. In the event of study termination or site termination, the central and local ethics committees/institutional review boards must be informed. If the study or a study site is closed before the planned end of the study, study materials (except documents required to be retained and stored on site) must be returned to the sponsor. The site will retain all other documents until notification is given by the sponsor and/or as required by the local regulatory authorities. If the study or a study site is closed prematurely, a Study Termination / Treatment Completion Form should be completed for all enrolled participants unless otherwise directed by the sponsor.

8.13 Incentives

Patients who enroll in the study will receive a $50 gift card in recognition of the additional time required for a patient who goes through the study process. In addition, participants who complete the evaluation at the end of the Cross-Over Period will receive an additional $50 gift card.

As noted in Section 3.1.3 Equipment - participants using personal phones will be provided an incentive equal to $10 a month for each month they use their phones, during the Cross-Over Period and Treatment Continuation Period, to reimburse for data usage while participating in this study. Participants will download the free video application in their phones.

Participants who do not own or have smart phone compatible with the DOT video applications will be provided a smart phone through the study. Participants who are loaned a phone through the study will not receive the incentive equal to $10 a month to reimburse for data usage. The reason the $10 a month incentive for data usage will not be provided is because the loaned phones, with accompanying data plans, are provided at no cost to the participant.

9 STUDY SCHEDULE

Screening, enrollment, study visits, and follow-up activities to be conducted are also shown in Appendix A.

A diagram that illustrates screening, enrollment, study-related activities is provided in Appendix B.

9.1 Screening

As noted in Section 8.3 Study Coordinators and Study Facilitators will work in tandem to identify potential participants.

As appropriate, case managers will identify patients transferred to the Corona, Washington Heights, Fort Greene and Morrisania TB Chest Center and inform the Study Coordinator. The Study Coordinator will alert Study Facilitators working in the appropriate clinic. Study Facilitators will check appointments in Digital Clinic to determine when these patients are scheduled for an appointment, and will search the same system for those patients who enter the clinic as a Class III or Class V patients.

Study facilitators will conduct a preliminary screening to determine if persons with a new or suspected diagnosis of TB meet the following inclusion criteria: age, Rif resistant TB is not suspected, individual is not incarcerated or institutionalized. The Screening Form may be used to facilitate tracking this information and to avoid duplicate screening efforts.

Demographic data (without identifying information) for those patients determined to be ineligible during preliminary screening and patients who refuse to discuss the study will also be entered into the Screening Form. Data from the Screening Form will be entered into the study database to ascertain reasons for non-enrollment.

Study Facilitators will inform the clinic physician that the patient is potentially eligible for inclusion, or leave a note with this information in the patient chart.

Clinic physicians will evaluate the patient, and discuss the study with the patient. Clinic nurses will then provide medication as needed to the patient and discuss the study further with the patient. If the patient is not interested in the study and declines further discussions, the clinic will follow normal procedures and the study facilitator will document the patient's refusal on the Screening Form.

If the patient is interested in the study, study facilitators will then meet with the individual to discuss the study in more detail, including inclusion and exclusion criteria and the risks and potential benefits of all study procedures. If study facilitators are satisfied that the potential participant understands the information and the potential participant is willing, study facilitators

will then review the Informed Consent Form (Appendix F) and ask each individual if he/she agrees to participate in the study.

If an individual declines study enrollment after speaking with study facilitators, he/she will be asked to share his/her reason for decline, and this will be documented in the Section C of the Screening Form. The clinic will then follow normal procedures to provide care and treatment to this individual.

If the study coordinator finds the potential participant is unable to understand the information presented, meets exclusion criteria, or does not meet study inclusion criteria, the individual should not be consented and enrolled in the study. Study facilitators will document non-enrollment on the Screening Form. The clinic will then follow normal procedures to provide care and treatment to this individual.

If a potential participant meets all inclusion criteria and agrees to enroll in the study following a review of the informed consent form, study facilitators will document this on the Screening Form and then complete the Enrollment and Baseline Visit Form.

Potential participants 18 years and older will be asked to provide written informed consent to participate in the study. Potential participants 12 to 17 years of age will be asked to provide assent, once assent is obtained a parent or legal guardian will be asked to provide written informed consent. After written informed consent, enrollment procedures will be initiated.

Note: Ideally, screening and the enrollment/baseline visit will occur concurrently. However, if a patient wishes to discuss the study and consent form with family or friends before making a final decision, enrollment may be delayed. Optimally, the time between screening and the enrollment/baseline visit will not exceed 10 business days. A patient may be enrolled 10 business days after screening at the discretion of the study coordinator and NYC DOHMH BTBC principal investigators.

If the screening visit occurred on a different date as the enrollment visit, sites should confirm that patients continue to meet eligibility criteria at the enrollment visit. Patients that do not meet the eligibility criteria at the enrollment/baseline visit are not to be enrolled.

Study facilitators will document the outcomes of this screening on the Screening Form.

Data obtained from preliminary screening activities, screening forms, enrollment and randomization activities will be used to complete the consort flow diagram provided in Appendix E.

9.2 Enrollment and Baseline Visit

After written informed consent has been obtained, the enrollment process will be completed as outlined below.

The two different eDOT video types (live video and recorded video) will be explained to patients in a standardized manner. The benefits and drawbacks of each video type will be addressed. Providers and patients will discuss the eDOT options and decide together the eDOT video type the patient will use. The decision and factors that influenced the decision will be documented on the Enrollment and Baseline Visit Form. Next, study facilitators will follow the procedures to randomize participants to a DOT study group assignment as outlined in Section 7.2 Randomization. Once the DOT study group assignment is known and documented, study facilitators will make arrangements as appropriate for participants to undergo eDOT-live, eDOT-recorded or ipDOT for the first part of the Cross-Over Period. Study facilitators will create and provide all participants with a schedule for DOT, as well as test DOT software and provide education in the use of the software to those participants initially assigned to eDOT.

The following information will also be collected and documented as part of the Enrollment and Baseline Visit Form.

9.2.1 Demographic and Contact Information

The following demographic information will be obtained from program records: gender at birth, age in years, ethnic origin, race, country of birth, month-year arrived in the U.S. (if foreign-born), preferred language, and primary occupation within the past year.

Participants will be interviewed by study facilitators in order to collect any demographic information not included in program records (e.g. educational attainment).

Additionally, study facilitators will obtain the following information: participant location of residence and phone number(s), and names and phone numbers of family members/friends who can be contacted by study facilitators in the event of emergency or if study facilitators are not able to locate the participant. Identifying and locating information will be maintained only at the site; this information will not be entered into the study data base.

9.2.2 Medical and Social History

Study facilitators will obtain the following medical and social history information from program records: site of current TB disease, evidence of cavity on a chest imaging study, sputum smear and culture for AFB; body tissue smear and culture for AFB in cases of extrapulmonary TB; previous treatment for latent or active TB; site of TB disease; concomitant diagnoses (i.e., diabetes, hepatitis, cancer, leukemia or lymphoma, chronic renal insufficiency, need to renal dialysis, gastrectomy / jejunoileal bypass, immunosuppression – not due to HIV/AIDS, history of mental illness); TB risk factors (i.e., homelessness, injecting drug use, non-injecting drug use within the past year, excess alcohol, prior incarceration, HIV, contact of infectious TB patient, contact to a person with multidrug-resistant TB, incomplete LTBI therapy, organ transplant, TNF-alpha antagonist therapy, tobacco use), setting in which participant was diagnosed with TB, and type of outpatient provider.

Participants will be interviewed by study facilitators in an attempt to collect any medical and social history information not included in program records. Study facilitators will also collect data on factors reported in the literature as being associated with treatment adherence, specifically the presence and type of social support (e.g., partner or family member involved with care, marital status, family size [20]), socio-economic factors (e.g., income, sources of financial support, education, housing, employment [20-22]) and psycho-social factors (e.g., psychological well-being, social functioning, health-related quality of life [21-22]).

9.2.3 Self-efficacy to Adhere to Treatment

Participants will be asked to complete a questionnaire to assess their self-efficacy to adhere to treatment. The questionnaire will be available in English and Spanish, and will be self-administered or completed with the assistance of a study facilitator. Additionally, this questionnaire will be completed before the participant is notified of his/her DOT study group assignment. The rationale for administering this questionnaire prior to the participant being notified of his/her DOT study group assignment is: (1) his/her DOT study group assignment could impact his/her responses to the questions; and (2) in the event a participant makes the decision to withdraw from the study after learning DOT study group assignment, these data will be used to compare those who withdraw with those who do not withdraw. These data will be examined in relation to patients' choice of method of DOT during the Treatment Continuation Period and treatment outcomes.

9.2.4 eDOT video type

Study facilitators will document the decision patients and providers make together regarding the eDOT video type patients will use.

9.2.5 Randomization

After demographic data, social and medical history data, self-efficacy, and eDOT video type data have been collected, staff will follow randomization procedures described in Section 7.2 Randomization. Each patient's randomized DOT study group assignment will be documented on the Master Participant List and the Enrollment-Baseline Visit Data Form.

9.2.6 eDOT Device

Study facilitators will also document participant's use of device for eDOT. This documentation will include whether the participant will use a personal smart phone, tablet, mini-tablet, notebook (see Section 3.1.3 Equipment), or a phone provided by the NYC DOHMH Bureau of TB Control, results of software tests, instruction provided to the participant. Additionally, patient proficiency using a smart phone and the DOT software application will be documented using a checklist that will indicate whether the patient was able to open the eDOT application, perform each step in the DOT process, and

close the application – with success, with additional education, or was unable to master the process.

These data will be reported to provide insight related to logistics and feasibility for TB programs that are considering implementation of an eDOT program.

9.3 Treatment Observation: Cross-over Period

Study staff, working in conjunction with program staff, will document scheduled DOT medication doses as either directly observed or not directly observed in the Digital Clinic system, which will be exported to the Scheduled DOT - Cross-over Period section of the study database.

For those doses that are directly observed, the method of DOT used (ipDOT, eDOT-live, eDOT-recorded); location of ipDOT (field or clinic); whether the patient was adherent to each dose (i.e., the patient was observed ingesting all medications as prescribed); and whether the DOT visit was a rescheduled visit will be documented.

Documentation of doses that are not directly observed will include whether the dose was expected to be directly observed or self-administered.

If a dose is expected to be directly observed but is not, and if the DOT visit was rescheduled, staff will record the reason as a technical issue (e.g., loss of internet connection, staff cannot clearly see the patient swallowing pills in a recorded video), a patient-related issue (e.g., missed DOT visit, patient forgets to video while taking medication), a staff issue (e.g., sick leave), or an environmental issue (e.g., storms that prevent travel).

Data regarding the day, timing, and adherence of each dose will be entered into the Digital Clinic systems within 24 business hours. Reasons why a dose is not directly observed or was rescheduled will also be entered within 24 business hours. This will be feasible as the NYC DOHMH BTBC staff use computers in the clinics, and tablets in the field, to document the medication taken, and the date and time medication is ingested during DOT visits. This information is documented in real time as the patient takes the medication using the Digital Clinic software system. This information is uploaded one time per day.

9.4 Cross-over Part 1 Completion Visit

Study facilitators and NYC DOHMH BTBC DOT staff will observe participants' treatment according to their Part 1 DOT study group assignment for 20 observable medication doses. Upon completion participants will undergo the Cross-over Part 1 Completion Visit.

During this visit, study facilitators will verify the participant's cross-over part 2 DOT study group assignment, make arrangements as needed for the participant's Part 2 DOT study group assignment (e.g., schedule ipDOT, add software app to smartphone), address any questions or concerns the participant may have, discuss medication side effects as needed, and document

the eDOT device to be used, as appropriate. The time to achieve 20 observable medication doses and dosing schedule will also be documented.

Data will be documented on the Cross-over Part 1 Completion Visit Form

Optimally, this visit will coincide with the participant's routine monthly clinic appointment. If the timing of a participant's routine monthly visit is incongruent with the end of Part 1 of the Cross-Over Period, an extra clinic appointment should be scheduled to conduct the Cross-over Part 1 Completion Visit.

9.5 Cross-over Part 2 Completion Visit

Study facilitators and NYC DOHMH BTBC DOT staff will again observe participant treatment according to their Part 2 DOT study group assignment for 20 observable medication doses. Upon completion, participants will undergo the Cross-over Part 2 Completion Visit.

During this visit, study facilitators will document the time to achieve 20 observable medication doses and dosing schedule, verify the participant's choice of DOT method for the duration of their TB treatment, record participants' rationale for their choice of DOT method, make arrangements as needed for the participant's choice of DOT method, address any questions or concerns the participant may have, and discuss medication side effects as needed. Data will be documented on the Cross-over Part 2 Completion Visit Form

Optimally, this visit will coincide with the participant's routine monthly clinic appointment. If the timing of a participant's routine monthly visit is incongruent with the end of Part 2 of the Cross-Over Period, an extra clinic appointment should be scheduled to conduct the Cross-over Part 2 Completion Visit.

Additionally, participants will be asked to complete a questionnaire that contains items addressing perceptions of the quality of care and degree to which their health care team has been supportive, patient-provider relationships/rapport, and overall satisfaction with eDOT-live, eDOT-recorded, and ipDOT during the Cross-Over Period. The questionnaire will be available in English and Spanish. To minimize the provision of socially-acceptable responses, this questionnaire will be self-administered. The Patient Opinion Questionnaire will be interviewer administered by the study facilitators for participants with lower literacy skills or request assistance completing the questionnaire.

9.6 Treatment Observation – TB Treatment Continuation Period

Study staff, working in conjunction with program staff, will document scheduled DOT medication doses as either directly observed or not directly observed in the Digital Clinic system, which will be exported to the Scheduled DOT - Continuation Period section of the study database.

The approach used will be the same as the approach described for weeks 1 through 8 in Section 9.3.

If at any point the patient wants to transfer to a different clinic for care, the patient may continue in the study. Treatment observation and study follow-up activities will be transferred to the study facilitator at the receiving clinic.

9.7 Documentation of Sputum Culture Conversion

Sputum culture conversion at 60 days, (defined as: one or more negative sputum cultures prior to 60 days after initiating therapy, not followed by a subsequent positive culture) will be assessed for those participants who had at least one sputum specimen culture positive for *Mtb* at enrollment.

Study sites will follow local procedures for sputum collection and testing. Study sites will also follow local policies and procedures in the event sputum culture is positive for *Mtb* 60 days after treatment initiation.

Sputum culture results for specimens collected 8-12 weeks after treatment initiation will be obtained from the NYC DOHMH BTBC Maven System and entered into the study database.

To enable assessments of sputum culture conversion in the context of this study, the information collected from the Maven System will include the date sputum specimens were collected and reported, when and where the patient initiated treatment, and the number of doses of anti-TB treatment a patient completed prior to study enrollment.

For those participants with a clinical diagnosis of TB (i.e., initial AFB smear and culture results not available) or a diagnosis of extrapulmonary TB, staff will document that the patient was enrolled based upon a clinical diagnosis in the appropriate section of the study database.

9.8 Documentation of Treatment Outcomes

Following treatment completion, participant's treatment outcomes will be obtained from the BTBC Maven EMR or TB registry and documented in the study database. Outcomes of interest include: treatment completion, cure, treatment failure, acquired drug resistance, lost to follow-up or refused further treatment, transfer, move, or expire (with death attributable to TB).

9.9 Documentation of Medication Side Effects

Symptom assessments during DOT live visits and clinic appointments will include asking participants whether they have experienced any of the following: anorexia / loss of appetite, nausea, vomiting, fatigue, weakness, muscle pain, jaundice, rash/itching, hives, red eyes, dark/orange urine, light colored stool, dry mouth, angioedema, peripheral neuropathy, petechiae, bruising, chills, malaise, headaches, dizziness, bone/joint pain, cough, sneezing, fever, night sweats, chest pain, shortness of breath, insomnia, abdominal pain, diarrhea, syncope, unexpected weight loss, and hemoptysis. For recorded eDOT, patients will be instructed to mention the presence or absence of side effects prior to taking the medication at every video recording.

In addition, participants will be asked whether they have had other symptoms (not listed above).

If a participant reports any symptoms, these symptoms will be recorded and graded according to severity using the National Cancer Institute Common terminology criteria for adverse events Version v4.03 [24] on the Monitoring Medication Side Effects Form.

This form will also be used to document time between initial symptoms of medication side effects and discussion with a medical provider (via conference calls with a provider through the eDOT-live system, non-routine TB clinic visits, urgent care center or emergency room visits, or hospital admission) . Additionally the form will document if treatment is discontinued for clinical/safety reasons by a clinician or program.

9.10 Focus Groups with Study Participants

As noted in Section 3.2 Patient Perceptions of DOT Methods, focus groups will be conducted with a convenience sample of study participants, to gather more in-depth information regarding patient perceptions of the quality of care received, rapport with TB program staff, satisfaction with the various methods of DOT, and suggestions for improvements. Ten to twenty participants will be invited to participate in focus group discussions and asked to provide written consent to participate in the focus group, separate from the randomized controlled trial's consent form (See Section 13.3 Informed Consent – Focus Groups and Appendix F). A total of 2-4 focus groups will be conducted, with 5 to 8 participants in each focus group.

Focus groups will be conducted in a location and time convenient to study participants willing to participate. An effort will be made to recruit an equal number of males and females, and an equal number of persons who received "live" and "recorded" eDOT during the Cross-Over Period.

Focus groups will be conducted by members of the study team. A semi-structured interview guide will be developed by the investigators to facilitate the group discussion. Focus group discussions will be audio recorded, and comprehensive field notes will be taken. Audio recordings and field notes will be transcribed and thematically analyzed. To complete the analysis, themes will not be imposed a priori. Instead, an initial list of codes will be developed by investigators based upon a preliminary review of participants' responses. Each participant's response will then be read independently by investigators, coded, and code frequencies will be generated, and results compared. Discrepancies will be discussed among the investigators and resolved.

Focus group participants will be provided $50 gift cards to compensate them for their time and $50 gift cards to compensate them for their transportation costs. Focus groups will be conducted at a time most convenient for the study team, in the last quarter of Year 1 or first quarter of Year 2.

9.11 Early Termination / Study Withdrawal

Study facilitators will complete a Study Termination / Treatment Completion Form for a participant in the following instances:

- A participant meets late exclusion criteria.
- A participant chooses to withdraw consent.
- The participant's medical care team feels study participation or eDOT is not in the best interest of the participant. I
- The patient expires
- The patient's treatment is permanently discontinued due to a medication side effect
- A patient is lost to follow-up or is incarcerated after enrollment, and continued study inclusion is not feasible.
- The study is discontinued or a study site is closed.

9.12 Study Termination / Treatment Completion

Study facilitators will complete a Study Termination / Treatment Completion Form when a participant either completes his/her TB treatment or discontinues study follow-up. This form will document the date of the last dose of treatment, treatment outcome, and reason the participant did not complete the study – as appropriate.

Optimally, this form will be completed during the participant's routine final appointment. If this form is completed after this visit and additional information is needed, an additional appointment at the clinic does not need to be scheduled. Instead, the participant may be called and asked to provide any needed information.

10 ASSESSMENT OF SAFETY

10.1 Overview

The primary focus of this study is to assess whether DOT that employs electronic communication methods is a non-inferior approach to monitor treatment adherence compared to traditional forms of DOT. It is also important to examine whether DOT that employs electronic communication methods is a non-inferior approach to monitor patients for medication side effects while undergoing treatment.

The antibiotics used to treat non-rifamycin resistant TB are generally well tolerated by patients. [23] However, as with any treatment, patients may experience medication side effects temporally associated with the use of the treatment. [24] Medication side effects can range from mild to severe, with urgent intervention required. As such, TB programs routinely monitor all patients for medication side effects throughout the course of treatment.

Thus this study will document type, severity, frequency, and time between initial symptoms of medication side effects and discussion with a medical provider (via conference calls with a provider through the eDOT-live system, non-routine TB clinic visits, urgent care center or emergency room visits, or hospital admission), for the most common medication side effects associated with the antibiotics used to treat non-rifamycin resistant TB. [25]

The side effects/symptoms include: anorexia, nausea, vomiting, fatigue, weakness, muscle pain, jaundice, rash/itching, hives, red eyes, dark/orange urine, light colored stool, dry mouth, petechiae, angioedema, peripheral neuropathy, bruising, chills, malaise, headaches, dizziness, bone/joint pain, cough, sneezing, fever, night sweats, chest pain, shortness of breath, insomnia, abdominal pain, diarrhea, loss of appetite, syncope, unexpected weight loss, and hemoptysis.

The severity of each medication side effect will be described using the common terminology criteria for adverse events (CTCAE). [24] According to the CTCAE, there are generally five levels of severity. While not all grades are appropriate for all medication side effects, the general description is provided in Table 4. The grade of each medication side effect will be assigned by the NYC principal investigators, following a review of data collected by study facilitators and data entered into patients' electronic medical records.

Medication side effects considered to be Grade 3 and higher will be reported to the IRB.

Table 4. Common terminology criteria for adverse events (CTCAE)

Grade 1	Mild; asymptomatic or mild symptoms; clinical or diagnostic observations only; intervention not indicated.
Grade 2	Moderate; minimal, local or noninvasive intervention indicated; limiting

	instrumental activities of daily living (i.e. preparing meals, shopping for groceries or clothes, using the telephone, managing money, etc.)
Grade 3	Severe or medically significant but not immediately life-threatening; hospitalization or prolongation of hospitalization indicated; disabling; limiting self-care activities of daily living (i.e. bathing, dressing and undressing, feeding self, using the toilet, taking medications, and not bedridden.)
Grade 4	Life-threatening consequences; urgent intervention indicated.
Grade 5	Death related to AE.

10.2 Specification of Measures

10.2.1 Primary Outcome Measure

Time (in days) between initial symptoms of medication side effects and receipt of medical attention either through consultation with a DOHMH BTBC physician (via the eDOT system, phone call, or clinic visit), urgent care or emergency room visits, or hospital admission.

10.2.2 Secondary Outcome Measure

Type of medication side effects while undergoing treatment with electronic methods of DOT, compared to participants on traditional forms of DOT.

Frequency of medication side effects while undergoing treatment with electronic methods of DOT, compared to participants on traditional forms of DOT.

Proportion of participants with medication side effects characterized as grade 3 or higher while undergoing treatment with electronic methods of directly observed therapy, compared to participants on traditional forms of directly observed therapy.

Frequency eDOT is discontinued for clinical/safety reasons by a clinician or program.

10.3 Methods and Timing for Assessing Medication Side Effects

Please see Section 9.9 Documentation of Medication Side Effects

10.4 Recording and Reporting Procedures

Please see Section 8 Study Procedures and Section 9 Study Schedule

10.5 Follow-up of Participants Following Medication Side Effects

Clinical care for the treatment of medication side effects will be performed in accordance with local program policies and procedures.

Participants who experience medication side effects that necessitate temporary discontinuation of their prescribed treatment will be retained in the study unless a clinician at a study site feels discontinuation is in the participant's best interest. Temporary discontinuation of the prescribed treatment will be documented on the Monitoring Medication Side Effects Form and in the NYC DOHMH BTBC digital clinic and will be imported to the study database for analysis.

Participants who experience medication side effects that necessitate permanent discontinuation of their prescribed treatment will be withdrawn from the study. Permanent discontinuation of treatment will be documented on the Study Termination / Treatment Completion Form.

11 STATISTICAL CONSIDERATIONS

11.1 Study Hypotheses

The main hypothesis of this study is below.

DOT that employs electronic communication methods (eDOT) is a non-inferior approach to monitor treatment adherence, compared to traditional forms of DOT, in which a trained person is in the physical presence of patients as anti-TB drugs are ingested (ipDOT).

The planned measures will result in the following comparisons:

11.2 Study Outcome Measures

Primary:

1. Among all prescribed doses of anti-TB medication, the proportion of doses that are directly observed by a study facilitator or staff member of the NYC DOHMH BTBC during the Cross-Over Period under the supervision of the NYC DOHMH BTBC by DOT method.

2. The reasons why DOT does not occur including:
    a. Equipment malfunction
    b. Equipment loss
    c. Staff unavailability
    d. Patient travel/ work/ school
    e. Patient uncooperative behavior
    f. Inclement weather
    g. Other reasons

Secondary:

1. The time in days between initial symptoms of medication side effects and receipt of medical attention by DOT Method.
    - Medical attention is defined as consultation with a DOHMH BTBC physician in the clinic, over the phone, or via video conferencing, as well as urgent care or emergency room visits, or hospital admission.

2. Proportion of sputum culture conversion within 60 days of treatment initiation by DOT method.

- Sputum culture conversion will be defined as: one or more negative sputum cultures prior to 60 days after initiating therapy, not followed by a subsequent positive culture.

3. Patient treatment outcome by DOT method during TB treatment continuation.

4. Patient perceptions of patient-provider relationships/rapport, and patients' self-efficacy to adhere by DOT method.

5. Patient and TB program staff perceptions of the quality of care by DOT method and overall satisfaction with eDOT and ipDOT

6. The rate at which clinicians decide to discontinue eDOT and reasons for their decision.

7. Patient choice of DOT method after the Cross-Over Period and patient characteristics associated with this choice.

8. Adherence to scheduled DOT sessions for ipDOT, eDOT-live, and eDOT-recorded during both the cross-over and continuation periods.

    - Per NYC DOHMH BTBC Policy, a scheduled DOT session will be considered successful if a patient is observed ingesting the full dose of prescribed medication on the scheduled day and time. When adherence to scheduled DOT sessions for patients undergoing treatment via ipDOT and eDOT-live is calculated, doses that are self-administered during weekends, national or state holidays, inclement weather and when program staff are unavailable, are excluded from the calculation.

9. DOT outcomes of patients who begin with eDOT first compared to DOT outcomes of patients who begin with ipDOT first.

11.3 Analysis Groups

There will be two major analysis groups and subgroups, as follows:

DOT Study Group Assignment 1: ipDOT: Doses of anti-TB drugs are ingested by patients in the presence of TB program staff who are trained to monitor treatment adherence.

This group will include both patients who ingest doses of anti-TB drugs in the presence of TB program staff within a TB clinic (ipDOT(Clinic-based)) as well as patients who ingest doses of anti-TB drugs in the presence of TB program staff at a patient's place of residence, workplace, or mutually-agreed upon meeting location (ipDOT(Community-based)).

Secondary data analyses will be conducted in which ipDOT (Community-based) and ipDOT (Clinic-based) are compared to each other and with eDOT. As appropriate, ipDOT (Clinic-based) will be considered the reference group.

DOT Study Group Assignment 2: eDOT: Doses of anti-TB drugs are ingested by patients, treatment adherence is monitored by TB program staff using electronic communication methods (this includes eDOT conducted in real time or with the use of recorded videos).

This group will include patients who undergo eDOT-live, in which doses of anti-TB drugs are ingested by patients, while TB program staff interact with patients via a computer or phone application in “real time” or “live” as they ingest their medication; as well as patients who undergo eDOT-recorded, in which doses of anti-TB drugs are ingested by patients, TB program staff log into an electronic system and review recorded videos to verify that patients ingested their medication doses as scheduled.

Secondary data analyses will be conducted in which eDOT-live and eDOT-recorded are compared to each other and to ipDOT. As appropriate, eDOT-live will be considered the reference group.

11.4 Analysis Plan

11.4.1 Primary analyses

The statistical analyses used will be those appropriate to reflect the study design. We anticipate the use of mixed-effects logistic regression, with the outcome variable being a binary variable reflecting whether intended DOT doses are directly observed by TB program staff, and including random subject-by-treatment effects to reflect between-subject variability within each treatment. Proportion of intended DOT doses directly observed by staff can then be tested between DOT treatments by statistical tests on fixed effects representing these proportions.

Some study participants may withdraw from the study prior to completion of all 40 doses. Data will be available for many or all of these participants, including those participants who withdraw from the study and allow collected data to be used, who are lost to follow up, who are incarcerated after enrollment, and those whose clinician felt it was in the best interest of the patient to withdraw from the study. We will use the data collected on these dropout participants in modified intention to treat analyses. That is, we will include their pre-dropout data in the analyses and consider their doses after dropout to be missing.

11.4.2 Secondary analysis

To evaluate the secondary objectives we will employ descriptive statistics in tabular form. We will perform t-tests, Chi-squared tests, and similar where needed to obtain p-values for testing between groups. Endpoints compiled will include proportion of patients achieving sputum conversion within 60 days of treatment initiation, medication side effects by severity rating, number of medication side effects, time between initial symptoms of medication side effects and receipt of medical attention, treatment completion rates by treatment, and a summary of patient treatment outcomes by important demographic and other available factors.

Additionally, per protocol analysis will be performed by using data for participants who completed the protocol as planned. Participants who completed 40 observable doses of treatment prior to: laboratory confirmation of rifamycin-resistant, multidrug-resistant, pre-XDR, or XDR TB; a provider determination ruling out TB disease; or discontinuation of TB treatment will be included in the per protocol analysis related to the cross-over period.

11.4.3 Analysis of Patients Perceptions of eDOT

Data will be entered into the study's Microsoft (MS) Access electronic database.

Responses to close-ended questions will be exported to SAS or SPSS and descriptive statistics will be used to summarize responses. Measures of central tendency (mean, median) and measures of spread (variance, interquartile range) will be calculated as appropriate.

Responses to open-ended questions will be exported to MS Excel, and thematic analysis will be performed. An initial list of codes will be developed by two investigators based upon a preliminary review of participants' responses. Each participant's response will then be read independently by two of three staff, coded, and results will be compared. Inter-rater reliability will be calculated through use of a kappa coefficient or intra-class correlation coefficient (ICC). Kappas/ICC of 0.70 or above for each code will be deemed acceptable. Discrepancies between the coders will be resolved through discussion. Frequencies for each theme that emerges will be calculated. A narrative will be written to describe the findings.

11.4.4 Data Agreements and Confidentiality Forms

If CDC staff provide instruction or guidance to study staff related to data collection or data analysis, and the potential exists for the CDC staff to view information containing patient identifiers; those staff will be required to sign and abide by the rules set forth in NYC DOHMH BTBC confidentiality forms.

CDC staff that assist with analysis of study data that may contain patient identifiers, will be required to complete and document appropriate training in the protection of human subjects. These staff will also be required to sign a data agreement form, acknowledging these data are NYC DOHMH BTBC. These staff will also be required to sign and abide by the rules set forth in NYC DOHMH BTBC confidentiality forms.

11.5 Sample Size Considerations

The primary objective of the trial is to evaluate whether DOT conducted by electronic means (eDOT) can attain a level of treatment adherence at least as favorable as DOT conducted traditionally, in person (ipDOT). Therefore, the trial is structured as a 2-arm, non-inferiority study.

First, we computed sample size under a parallel design. Our starting point was the following formulas for sample size and power, respectively [26-27]:

$$n_A = \kappa n_B \text{ and } n_B = \left(\frac{p_A(1-p_A)}{\kappa} + p_B(1-p_B)\right)\left(\frac{z_{1-\alpha} + z_{1-\beta}}{p_A - p_B - \delta}\right)^2$$

$$1-\beta = \Phi\left(z - z_{1-\alpha/2}\right) + \Phi\left(-z - z_{1-\alpha/2}\right) \quad , \quad z = \frac{p_A - p_B - \delta}{\sqrt{\frac{p_A(1-p_A)}{n_A} + \frac{p_B(1-p_B)}{n_B}}}$$

<u>Where</u>

$\kappa = n_A/n_B$ is the matching ratio

ϕ is the standard normal distribution function

ϕ^{-1} is the standard normal quantile function

α is Type I error

β is Type II error, meaning $1-\beta$ is power

δ is the testing margin

We then modified the above formula to account for pooled variance. [28] Finally we reduced the sample size due to the effect of a cross-over design. [29]

The required input parameters for these calculations include the following variance parameters: between-subject variance for both treatment groups, the correlation between a person's treatment and reference repetitions under the cross-over design, and the within-person variance for both treatment groups. We found that the reduction in sample size due to the cross-over design is influenced mostly by the correlation parameter; we assumed it to take a moderate value of 0.40.

			**Sample Size**			
Treatment Completion Rate	Power		eDOT	DOT	Total Under Parallel Design	Total Under Cross-over Design
Non-Inferiority Margin = -0.08						
0.85	0.8		313	313	626	421
0.85	0.85		358	358	716	481
0.85	0.9		419	419	838	563
0.9	0.8		221	221	442	297
0.9	0.85		253	253	506	340
0.9	0.9		296	296	592	398
Non-Inferiority Margin = -0.10						
0.85	0.8		201	201	402	270
0.85	0.85		229	229	458	308
0.85	0.9		268	268	536	360
0.9	0.8		142	142	284	191
0.9	0.85		162	162	324	218
0.9	0.9		190	190	380	256*

Based on the following 2 considerations, we will increase our target enrollment by 15%, per arm.

<u>Consideration 1</u>: The proportion of enrolled patients who would be found to be late exclusions due to microbiological ineligibility: 2%

We anticipate 0.98% of cultures will identify multidrug-resistant or extremely drug resistant TB [6] and an estimated 1.1% of cases whose cultures will contain nontuberculous mycobacteria (NTM). Note this is an estimate only as NTM are not reportable and the incidence of NTM in the United States is unknown.

<u>Consideration 2:</u> Proportion of enrolled patients who would be found to be 'not assessable' due to non-completion of treatment: 13% [29]

We anticipate that the 4 participating clinical sites can successfully enroll 304 participants for this study, given that the NYC DOHMH, Bureau of TB Control reported an average of 655 cases of TB per year over the past 5 years. Moreover, we base the following expectations on 2014 data in which the NYC BTBC prescribed TB treatment to 639 persons diagnosed with TB and 576 (90.1%) completed treatment:

With a targeted enrollment of 304 participants (152 participants per arm), and expected attrition of 15% (based on the 2 considerations outlined above), we expect to have assessable data for 256 to 258* participants (128 participants per arm).

We expect to have 90% power to test the primary hypotheses among the assessable subgroup. The 10% margin to define inferiority does not imply that the experimental regimen may result in as much as 10% more unfavorable outcomes, but rather, for a fixed design, the maximum difference consistent with a non-inferior conclusion decreases as the proportion of unfavorable outcomes in the control arm increases.

12 QUALITY CONTROL AND QUALITY ASSURANCE

Study staff will be trained in Good Clinical Practice and in performance of study procedures. The sponsor or the sponsor's delegate will conduct a meeting with the site prior to the study opening to ensure that everything is in place for the study to start, the study file contains all the essential documents, and study staff understand procedures and their roles and responsibilities. The study will be conducted in accordance with the protocol and a study-specific manual.

12.1 Data Quality Management

To ensure the data acquired, created, and maintained as part of this study are both accurate and complete, (1) study staff will receive training in data collection and data clarification, (2) the study coordinator will routinely compare study data with data sources external to and independent of the study data, and (3) the study database will be programmed to perform electronic data checks.

Specifically, study staff will undergo training to orient them to the study protocol, data collection forms, data entry, source documentation, good research documentation, data validation, reporting medication side effects, and data clarification processes. To further ensure data is submitted correctly and promptly, the study coordinator and data analyst will be responsible for promptly responding to any emails or phone calls from study facilitators. Additionally, study facilitators will have access to a manual received during their training and data coding guidelines.

Training will be ongoing as needed to address any amendments to the protocol, design of forms, and in the event an investigator or the study coordinator notes that a staff member provides incomplete, excessive, or ambiguous data. Staff training will be documented and maintained in the study file by the study investigators and research coordinator.

To validate and detect errors within the data, study facilitators will edit check all data collection forms and electronic data entry made to the clinic site's study database. These edit checks will include checking that the data is accurate and entered in the correct location or data field, inspecting for missing values, reviewing for consistency within and across forms (e.g., making sure the birth date matches the participants age), checking that only authorized abbreviations are used, ensuring alterations have been properly made, and confirming all information is spelled correctly and is legible.

The study coordinator will perform routine data audits comparing the data entered onto study data collection forms with source documents external to and independent of the research forms (e.g., medical records, laboratory reports, and pharmacy dispensing records). These data audits will check for logical errors (e.g., the date of a second visit is earlier than the date recorded for a first visit), accuracy of information, omissions, transcription errors, inappropriate abbreviations, spelling errors, illegible entries, and proper alterations.

The data analyst will import data from the 4 clinic site study databases into a centralized database and review the data for errors using programmed electronic data checks and reviews of the data. If any inconsistencies are found or clarification is needed, the data analyst will generate a data query and send the query to the study facilitators responsible for that clinical site. The study facilitators will investigate these queries and make corrections as needed to the research form and/or the clinic site's study database. As needed, facilitators will provide information to the data analyst. The information provided will be documented using the data query form

12.2 External monitoring

Visits will be conducted periodically by sponsor staff. Direct access to data at each site will be required for the purposes of monitoring and audit, and this will be made explicit in the consent and authorization for use/disclosure of protected health information for research forms. Local investigators and their institutions will provide direct access to source documents and data for study-related monitoring, audit, and regulatory inspections, in the clinic, the pharmacy, and the mycobacteriology laboratory.

Monitoring will focus on ensuring that the following study activities are conducted per the protocol and associated documents: consent procedures, enrollment, accurate recording and reporting of supervised treatment and medication side effects, and timely and accurate data entry. Additional activities or study elements may be monitored as needed.

12.3 Study Data Sources

The NYC DOHMH BTBC enters patient data into 2 record systems.

The first system, MAVEN is the TB registry used for case management activities and TB surveillance. Data is entered in "real time" into this system, which can collect up to 2,400 variables for each patient. This system includes all laboratory data that downloads directly into this system. Effectively, the data in the MAVEN system constitutes the patient's public health records.

The second system, Digital Clinic is the electronic medical record (EMR) used primarily to monitor and document treatment. Adherence to each dose is entered in real time, using tablets when ipDOT is conducted outside of clinical settings, and on computers when eDOT is conducted. Data entered into the Digital Clinic system is backed up daily.

In the event the Digital Clinic is not available, BTBC staff use the NYC DOHMH Directly Observed Therapy Medication Log. Data entered by hand into this paper system is then entered into the Digital Clinic system as soon as possible.

Data from these two systems will be exported into MS EXCEL using SAS code and imported into the planned study-specific MS Access Database.

NOTE: The Digital Clinic and MAVEN systems operate separately and do not interconnect. In the event data entered into Digital Clinic is discordant with data in MAVEN, the Study Coordinator and Study Facilitators will review both MAVEN and Digital Clinic to reconcile the discordance and update the study database as appropriate. If staff are unable to reconcile the problem, the data in MAVEN will be considered the primary source.

13 ETHICS/PROTECTION OF HUMAN SUBJECTS

This study will be conducted in conformity with the ethical standards set out in the latest version of the Declaration of Helsinki.

13.1 Institutional Review Board

Each participating institution will provide for the review and approval of this protocol and the associated informed consent documents by an appropriate institutional ethics committee (IEC) or Institutional Review Board (IRB). Any amendments to the protocol or consent materials must also be approved before they are placed into use.

13.2 Informed Consent Process – Non-inferiority Study

Adults: Only individuals who provide written informed consent will be enrolled in this study. Written informed consent is required before any study-specific procedures are performed. Potential participants will have the conditions of the study explained to them, including potential harms and benefits, the nature and timing of study procedures, alternatives to study participation, that study participation is voluntary, that a decision to not participate in the study will not affect the quality of their future medical care, and that they may withdraw from participation at any time.

The information in the Informed Consent document will be translated into relevant languages spoken and read locally by certified translators.

Literate individuals will be provided with a language-appropriate document to read; illiterate individuals (i.e., individuals who speak and understand, but do not read and write, the language in which the consent discussion is conducted) will have the contents of the document explained to them by a trained study staff member; such individuals can be enrolled by 'making their mark' on the consent document.

Potential participants will have the opportunity to ask questions of an investigator or delegate, and to discuss participation with their family and/or friends or think about the study prior to deciding whether or not to participate. A copy of the signed informed consent document will be given to the participant for his/her records.

Please see the Consent Form in Appendix F. Reading comprehension for this form was estimated using an online document readability program (https://www.online-utility.org/english/readability_test_and_improve.jsp). The approximate U.S. grade level needed to comprehend the text was between 6.50 per the Automated Readability Index and 8.79 according to the SMOG. The Flesch Kincaid Grade Level was estimated to be 7.95, and the Flesch Reading Ease Score indicates approximately 65.86% of the population could read and easily comprehend the consent form.

Children: For potential participants under 18 years of age, the assent of the child as well as the written informed consent of the child's legal guardian will be required for enrollment in this study. The child will receive, in language appropriate to the age and maturity of the child, an explanation of the research procedures; a description of the risks, discomforts, or inconveniences that the child might experience; and assurance that the child can withdraw from the study at any time. The assent process will be conducted by a study staff member who is experienced in consent and assent procedures, and in accordance with IRB requirements. All study participants age < 18 years will provide written assent and written consent by the participant's legal guardian, in accordance with IRB requirements.

The information in the Informed Consent and Assent documents will be translated into relevant languages spoken and read locally by certified translators.

Please see the Consent / Assent Forms in Appendix F. Reading comprehension for these forms was estimated using an online document readability program (https://www.online-utility.org/english/readability_test_and_improve.jsp).

The approximate U.S. grade level needed to comprehend the text contained in the Parent Consent Form was between 7.07 per the Automated Readability Index and 8.85 according to the SMOG. The Flesch Kincaid Grade Level was estimated to be 7.93, and the Flesch Reading Ease Score indicates approximately 67.65% of the population could read and easily comprehend the consent form.

The approximate U.S. grade level needed to comprehend the text contained in the Child Assent Form was between 4.24 per the Automated Readability Index and 7.72 according to the SMOG. The Flesch Kincaid Grade Level was estimated to be 6.50, and the Flesch Reading Ease Score indicates approximately 73.45% of the population could read and easily comprehend the consent form.

13.3 Informed Consent – Focus Groups

The convenience sample of participants invited to participate in the focus groups (see Section 9.10) will also be asked to provide written informed consent, separate from the randomized controlled trial's consent form. Focus groups will be restricted to participants equal to or greater than 18 years old who are conversant in English. Potential participants will have the conditions of the focus groups explained to them, including potential harms and benefits, the nature of focus group procedures, the ability to use any name they choose during discussions to protect their privacy, actions the study team will take to ensure confidentiality, alternatives to focus group participation, that focus group participation is voluntary, that a decision to not participate in the focus group will not affect the quality of their future medical care, and that they may withdraw from participation at any time.

The information in the Informed Consent document will be available in English as focus groups will be restricted to English participants.

Literate individuals will be provided with a language-appropriate document to read; illiterate individuals (i.e., individuals who speak and understand, but do not read and write English) will have the contents of the document explained to them by a trained study staff member; such individuals can be enrolled by 'making their mark' on the consent document.

Potential participants will have the opportunity to ask questions of an investigator or delegate, and to discuss participation with their family and/or friends or think about the focus groups prior to deciding whether or not to participate. A copy of the signed informed consent document will be given to the participant for his/her records.

Please see the Consent Form in Appendix F. Reading comprehension for this form was estimated using an online document readability program (https://www.online-utility.org/english/readability_test_and_improve.jsp). The approximate U.S. grade level needed to comprehend the text was between 6.55 per the Automated Readability Index and 9.49 according to the SMOG. The Flesch Kincaid Grade Level was estimated to be 7.89, and the Flesch Reading Ease Score indicates approximately 62.50% of the population could read and easily comprehend the consent form.

13.4 Subject Confidentiality

The Research Team will maintain Confidential Information and Participant Information in a secure facility, taking commercially reasonable steps to protect such information from unauthorized use, access and disclosure. This will include the use of locks on office doors and file cabinets, password protected computers and files, encryption, and HIPAA compliant applications.

Further, all records identifying the participant will be kept confidential and, to the extent permitted by the applicable laws and regulations, will not be made publicly available without sufficient de-identification procedures.

Participant names will not be supplied to the sponsor. All study documents and forms will be identified by a code only. All paper study records will be stored in a locked office and electronic study records will be stored on password-protected computers; only designated trained study staff will have access to study records. Transmission of electronic records to the sponsor will occur through applications that conform to the Federal Information Security Management Act, or using a secure CDC File Transfer Protocol (FTP).

Authorized representatives of the sponsor, and regulatory authorities may inspect all documents and records required to be maintained by the Investigator, including but not limited to, medical records, primary laboratory data, and pharmacy records for study participants; this information will be provided to participants during the Informed Consent process. The clinical study site will permit access to such records.

13.5 Study Discontinuation

CDC DTBE has the right to close the study and the right to close a study site. If the study is closed, all involved ethics committees should be notified. If a site is closed, then the local IEC/IRB and the CDC IRB should be notified. In the event that the study is discontinued or a study site is closed, participants will undergo a study termination visit. Participants receiving treatment at the time of study discontinuation will continue treatment according to local procedures for TB care under the direction of the NYC DOHMH BTBC.

14 DATA HANDLING AND RECORD KEEPING

Data handling and record keeping will entail recording information from participants and source documents onto study-specific case report forms, which will be entered into an MS Access database designed for this study. The study electronic information system will be programmed to maintain an audit trail, perform consistency checks, and generate reports of missing/inconsistent data. After study completion, de-identified data that can be legally released to the public may be released through a public-use data set after the data are evaluated for quality and confidentiality and shared with any partners, per CDC's policy on data sharing.

The study file and source documents will be retained at the NYC DOHMH BTBC office according to local IRB policies.

15 ROLES AND RESPONSIBILITIES OF STUDY TEAM

15.1 Study Sponsor: Division of Tuberculosis Elimination, National Center for HIV/AIDS, Viral Hepatitis, STD, and TB Prevention, U.S. Centers for Disease Control and Prevention

The roles and responsibilities of the study sponsor are as follows:

- Collaborate with the NYC DOHMH BTBC research team's Principal Investigators to design the study, write the study protocol, create data collection tools, and analyze data.
- Provide the NYC DOHMH BTBC research team with the information and technical assistance needed to conduct the study and analyze the results.
- Ensure that an Institutional Review Board (IRB) that complies with the requirements of 21 CRF Part 56 will be responsible for the initial and continuing review and approval of the study.
- Ensure the overall quality of the research data is verifiable and acceptable for public reports and publications.
- Maintain and retain adequate records and reports.
- Collaborate with the Research Team to publish and disseminate study findings.

15.2 NYC DOHMH BTBC Research Team

The primary responsibilities of the Research Team are as follows:

- Ensure that all source data is documented in the Medical Record/Research Chart with accuracy, completeness, and consistency;
- Ensure the overall quality of the research data is verifiable and acceptable for public reports and publications, etc.;
- Review data discrepancy/clarification resolutions for accuracy, consistency and timely response.

The roles and responsibilities of team members are outlined below:

15.2.1 Principal Investigators

The Principal Investigators:

- Agree to collaborate with CDC to design the study, write the study protocol, create data collection tools, and analyze data.
- Agree to conduct the study in accordance with the relevant, current protocol and will only make changes in a protocol after notifying the sponsor, except when necessary to protect the safety, rights, or welfare of participants.

- Agree to ensure all associates, colleagues, and employees assisting in the conduct of the study are informed about their obligations.
- Agree to maintain adequate and accurate study records and to make those records available for inspection by the study sponsor.
- Agree to promptly report to the IRB all changes in the research activity and all unanticipated problems involving risks to human subjects.
- Agree to not make any changes in the research protocol without IRB approval, except when necessary to eliminate apparent immediate hazards to study participants.
- Agree to collaborate with CDC to publish and disseminate study findings.
- The responsibilities of the Principal Investigators are to:
- Ensure informed consent and authorization for use/disclosure of protected health information for research is obtained from a patient prior to the performance of any study-related procedure.
- Ensure the Informed Consent Document and Authorization for Use/Disclosure of Protected Health Information for Research Document was approved by an IRB and the correct version of the forms is being used.
- Comply with the IRB approved protocol.
- Maintain study material accountability.
- Supervise conduct of the study.
- Assess the severity of participants' medication side effects using the common terminology criteria for adverse events (CTCAE) following a review of data collected by study facilitators and data entered into patients' electronic medical records.
- Maintain adequate participant records and research forms.
- Enroll the adequate number of study participants required by the sponsor.
- Archive study-related documents for the time period as required by regulations.

15.2.2 Study Coordinator

The Study Coordinator (SC) will:

- Participate in meetings with investigators and sponsors.
- Review and familiarize themselves, and other study staff with the study protocol.
- Prepare and submit paperwork required to obtain and maintain IRB approval.
- Abide by the IRB and Informed consent regulations.

- Verify that the most current IRB-approved study consent/assent and authorization for use/disclosure of protected health information for research documents are available for use.
- Establish and maintain accurate and complete study files.
- Ensure site files are updated as appropriate when changes to licenses, IRB documents or CVs are made during the study. The study coordinator will review the site files every 3 months to verify that all documents (paper and electronic) are maintained.
- Ensure that he/she and all study personnel have completed all required institution-specific and protocol-specific trainings and that these trainings are documented appropriately on the Training Log and Staff training certificates are stored in the Essential Documents Binder.
- Personally oversee study activities.
- Ensure study facilitators correctly randomize study participants so as to avoid biases.
- Assist as needed with participant enrollment and study activities.
- Review consent documentation and confirm adherence to consent processes.
- Review completion and accuracy of the source documents and research forms for subjects at the site on a monthly basis.
- Respond to all data queries as needed.
- Ensure study records are maintained in a manner that protects the privacy of all study participants.
- Compile the required information for the sponsor.
- Close the study with the sponsor and investigators, and store study records as appropriate.

15.2.3 Data Analyst

The Data Analyst will:

- Assist in the design of study specific forms, and annotation of these forms.
- Design and create a study specific database.
- Establish and adhere to procedures and controls to ensure the integrity, authenticity, and confidentiality of the study data as outlined by the Society for Clinical Data Management's (SCDM) Good Clinical Data Management Practices Guidelines.
- Perform data-importation/entry, data validation, discrepancy management, coding, data extraction, and database locking.

- Maintain an audit trail of data management activities.
- Monitor protocol accrual.
- Provide investigators with interim and summary reports.

15.2.4 Study Facilitators

The Study Facilitators will:

- Complete CITI, NIH, or Columbia University Human Subjects training prior to commencement of study activities. In addition, staff are also required to complete HIPAA training.
- Review and familiarize themselves with the study protocol.
- Complete training on protocol-specific informed consent, randomization procedures, data collection procedures, administering questionnaires, and data entry.
- Recruit and enroll study participants according to procedures approved by the IRB.
- Prior to a visit, verify the required elements of the visit. At the completion of a visit, verify all required elements have been fulfilled.
- Conduct study visits with participants. During these visits, collect data, make arrangements for participants as appropriate, provide education to participants as appropriate, and monitor for medication side effects.
- Conduct DOT visits via eDOT-recorded, eDOT-live, and ipDOT in the clinic only. NOTE: Study facilitators will not conduct ipDOT in the field as this would remove them from the clinic setting and could interfere with other study responsibilities (e.g., screening and enrollment).
- Abstract data as needed from participants medical charts.
- Enter data onto protocol-specific forms and/or specifically designed computerized data-entry screens.
- Maintain all study records and materials in a manner that protects participant privacy and patient confidentiality per local regulations.
- Assure the quality and the integrity of the study data.
- Respond to data queries promptly.
- Treat all study participants with respect.

1839

16 TIMELINE

	2016						2017												2018											
Tasks	April	May	June	July	Aug	Sept	Oct	Nov	Dec	Jan	Feb	March	Apr	May	June	July	Aug	Sept	Oct	Nov	Dec	Jan	Feb	March	Apr	May	June	July	Aug	Sept
**Primary Study**																														
Disperse funds from CDC to VA	■	■	■	■	■																									
Disperse funds from VA to NY					■	■	■																							
Start-up / Develop Protocols	■	■	■	■	■	■	■	■	■	■																				
Development of Data collection forms								■	■	■	■																			
Finalize Data Collection Forms								■	■	■	■	■																		
Finalize Protocol									■	■	■	■																		
Submit exemption to OMB review – clinical exemption under the PRA											■	■																		
Prepare IRB application for CDC IRB											■	■																		
Prepare IRB application for Columbia Univ. IRB											■	■																		
Prepare IRB application for NYC DOHMH IRB											■	■																		
IRB Review Approval												■	■																	
Translation of data collection forms												■	■																	

	2016						2017												2018											
Tasks	April	May	June	July	Aug	Sept	Oct	Nov	Dec	Jan	Feb	March	Apr	May	June	July	Aug	Sept	Oct	Nov	Dec	Jan	Feb	March	Apr	May	June	July	Aug	Sept
Development of Study Database						■	■	■	■	■	■	■																		
Study Staff Training									■	■	■	■																		
Initiate Recruitment														■																
Study Recruitment														■	■	■	■	■	■	■	■	■	■	■	■	■	■	■	■	■
Data collection														■	■	■	■	■	■	■	■	■	■	■	■	■	■	■	■	■
Prepare IRB Renewal																						■	■							
Submit IRB Renewal																							■	■						
Prepare & submit IRB final report																														
Data cleaning														■	■	■	■	■	■	■	■	■	■	■	■	■	■	■	■	■
Preliminary Analyses Primary Objectives																		■	■	■	■	■	■	■	■	■	■	■	■	■
Final Analyses Primary Objectives																													■	■ ➔
Preliminary Analyses Secondary Objectives																		■	■	■	■	■	■	■	■	■	■	■		
Final Analyses Secondary Objectives																													■	■ ➔
Dissemination of Study Findings / Manuscript Prep																														➔

	2019												2020											
Tasks	Oct	Nov	Dec	Jan	Feb	March	April	May	June	July	Aug	Sept	Oct	Nov	Dec	Jan	Feb	March	April	May	June	July	Aug	Sept
Study Recruitment	■	■	■	■	■	■	■	■	■	■														
Data collection	■	■	■	■	■	■	■	■	■	■	■	■												
Prepare IRB Renewal					■	■																		
Submit IRB Renewal						■																		
Prepare & submit IRB final report																								
Data cleaning	■	■	■	■	■	■	■	■	■	■	■	■	■	■	■	■	■	■						
Preliminary Analyses Primary Objectives	■	■	■	■	■	■	■	■	■	■	■	■	■	■	■	■	■	■	■					
Final Analyses Primary Objectives	■	■	■	■	■	■	■	■	■	■	■	■	■	■	■	■	■	■	■	■	■	■		
Preliminary Analyses Secondary Objectives	■	■	■	■	■	■	■	■	■	■	■	■	■	■	■	■	■	■	■	■	■	■	■	
Final Analyses Secondary Objectives	■	■	■	■	■	■	■	■	■	■	■	■	■	■	■	■	■	■	■	■	■	■	■	
Dissemination of Study Findings / Manuscript Prep	■	■	■	■	■	■	■	■	■	■	■	■	■	■	■	■	■	■	■	■	■	■	■	■ →

17 PUBLICATIONS AND DISSEMINATION OF STUDY RESULTS

Both the NYC DOHMH BTBC and CDC investigators must approve the use of any study data for the purposes of publication or presentation in advance.

Any proposal for additional analysis of study data must be agreed to in advance by the NYC DOHMH BTBC and CDC investigators.

These criteria will not apply to public-use data that have been made available in accordance with CDC's policy on data sharing. Persons who use publicly available data will be asked to acknowledge both the NYC DOHMH BTBC and CDC.

Updates on the progress of the study will be presented at CDC Division of Tuberculosis Elimination weekly seminars. Additional dissemination of results will be through the press, national professional society meetings and international conferences.

Overall (aggregate) study results will be shared with study participants through mechanisms and materials reviewed and approved by the by the NYC DOHMH BTBC and CDC investigators and protocol team.

18 LITERATURE REFERENCES

1. Reported Tuberculosis in the United States, 2014. Atlanta, GA: U.S. Department of Health and Human Services, CDC, October 2015

2. Tupasi TE, Garfin AMCG, Mangan JM, Naval LC, Pancho JS, Mantala MJ, Kurbatova EV, Golubkov A. (2015) Patients' perceptions of interventions to improve MDR TB treatment completion in the Philippines. Abstract]. The International Journal of Tuberculosis and Lung Disease. Supplement 2. Volume 19, Number 12. Oral Abstract Session (OA-321-04) at the 46th Union World Conference in Cape Town, South Africa (December 2015) http://capetown.worldlunghealth.org/Abstract_Book_2015-Web.pdf

3. Chida N, Ansari Z, Hussain H, Jaswal M, Symes S, Khan AJ, Mohammed S. Determinants of Default from Tuberculosis Treatment among Patients with Drug-Susceptible Tuberculosis in Karachi, Pakistan: A Mixed Methods Study. PLoS One. 2015 Nov 12; 10(11):e0142384. doi: 10.1371/journal.pone.0142384. eCollection 2015

4. Roy N, Basu M, Das S, Mandal A, Dutt D, Dasgupta S. Risk factors associated with default among tuberculosis patients in Darjeeling district of West Bengal, India. J Family Med Prim Care. 2015 Jul-Sep; 4(3):388-94. doi: 10.4103/2249-4863.161330.

5. Muture BN, Keraka MN, Kimuu PK, Kabiru EW, Ombeka VO, Oguya F. Factors associated with default from treatment among tuberculosis patients in Nairobi province, Kenya: a case control study. BMC Public Health. 2011 Sep 9; 11:696. doi: 10.1186/1471-2458-11-696.

6. Frieden TR, Sbarbaro JA. Promoting adherence to treatment for tuberculosis: the importance of direct observation. Bull World Health Organ. 2007; 85:407-9

7. Moonan PK, Quitugua TN, Pogoda JM, Woo G, Drewyer G, Sahbazian B, Dunbar D, Jost KC Jr, Wallace C, Weis SE. Does directly observed therapy (DOT) reduce drug resistant tuberculosis? BMC Public Health. 2011; 11:19. doi: 10.1186/1471-2458-11-19

8. Weis SE, Slocum PC, Blais FX, King B, Nunn M, Matney GB, Gomez E, Foresman BH. The effect of directly observed therapy on the rates of drug resistance and relapse in tuberculosis. N Engl J Med. 1994; 330:1179-84

9. Frieden TR, Fujiwara PI, Washko RM, Hamburg MA. Tuberculosis in New York City--turning the tide. N Engl J Med. 1995; 333:229-33

10. Bayer R, Wilkinson D. Directly observed therapy for tuberculosis: history of an idea. Lancet. 1995; 345:1545-8

11. Chaulk CP, Kazandjian VA. Directly observed therapy for treatment completion of pulmonary tuberculosis: Consensus Statement of the Public Health Tuberculosis Guidelines Panel. JAMA. 1998; 279:943-8

12. Volmink J, Garner P. Directly observed therapy for treating tuberculosis. Cochrane Database Syst Rev. 2007; (4):CD003343

13. Karumbi J, Garner P. Directly observed therapy for treating tuberculosis. Cochrane Database Syst Rev. 2015; 5:CD003343. doi: 10.1002/14651858.CD003343.pub4

14. Ngamvithayapong J, Yanai H, Winkvist A, Saisorn S, Diwan V. Feasibility of home-based and health centre-based DOT: perspectives of TB care providers and clients in an HIV-endemic area of Thailand. Int J Tuberc Lung Dis. 2001 Aug; 5(8):741-5.

15. Khan MA, Walley JD, Witter SN, Shah SK, Javeed S. Tuberculosis patient adherence to direct observation: results of a social study in Pakistan. Health Policy Plan. 2005 Nov; 20(6):354-65. Epub 2005 Sep 23.

16. Garfein RS, Collins K, Muñoz F, Moser K, et.al. Feasibility of tuberculosis treatment monitoring by video directly observed therapy: a binational pilot study. Int J Tuberc Lung Dis. 2015; 19:1057-64

17. Chuck C, Robinson E, Macaraig M, Alexander M, Burzynski J. Enhancing management of tuberculosis treatment with video directly observed therapy in New York City. Int J Tuberc Lung Dis. 2016. 20(5): 588-593. http://dx.doi.org/10.5588/ijtld.15.0738

18. Garfein RS, Collins K, Muñoz F, Moser K, Cerecer-Callu P, Raab F, et al. Feasibility of tuberculosis treatment monitoring by video directly observed therapy: a binational pilot study. Int J Tuberc Lung Dis. 2015; 19:1057–64. http://dx.doi.org/10.5588/ ijtld.14.0923

19. Story A, Garfein RS, Hayward A. et al. Monitoring Therapy Adherence of Tuberculosis Patients by using Video-Enabled Electronic Devices. Emerging Infectious Diseases. Vol. 22, No. 3, March 2016. P 538-540. DOI: http://dx.doi.org/10.3201/eid2203.151620

20. Chen B, Peng Y, Zhou L., et al. Social support received by multidrug-resistant tuberculosis patients and related factors: a cross-sectional study in Zhejiang Province, People's Republic of China. Patient Preference and Adherence. 2016; June 13:1063-1070.

21. Kastien-Hilka T, Abulfathi A, Rosenkranz B, et al. Health-related quality of life and its association with medication adherence in active pulmonary tuberculosis– a systematic review of global literature with focus on South Africa. Health and Quality of Life Outcomes. 2016; 14: 42. DOI 10.1186/s12955-016-0442-6.

22. Birch S, Govender V, Fried J, et al. Does treatment collection and observation each day keep the patient away? An analysis of the determinants of adherence among patients with Tuberculosis in South Africa. Health Policy and Planning. 2016; 31: 454-461. doi: 10.1093/heapol/czv084

23. Blumberg HM, Burman WJ, Chaisson RE et. al. American Thoracic Society/Centers for Disease Control and Prevention/Infectious Diseases Society of America: treatment of tuberculosis. Am J Respir Crit Care Med. 2003 Feb 15; 167(4):603-62.

24. National Cancer Institute. Common terminology criteria for adverse events v4.03. June 2010. http://evs.nci.nih.gov/ftp1/CTCAE/CTCAE_4.03_2010-06-14_QuickReference_5x7.pdf. Accessed April 25, 2016.

25. Micromedex® Medication, Disease and Toxicology Management. Truven Health Analytics information, www.micromedexsolutions.com/ accessed April 26, 2016

26. http://powerandsamplesize.com/Calculators/Compare-2-Proportions/2-Sample-Non-Inferiority-or-Superiority

27. Chow S, Shao J, Wang H. 2008. *Sample Size Calculations in Clinical Research*. 2nd Ed. Chapman & Hall/CRC Biostatistics Series. Page 90.

28. http://ncss.wpengine.netdna-cdn.com/wp-content/themes/ncss/pdf/Procedures/PASS/Non-Inferiority_Tests_for_the_Difference_Between_Two_Proportions.pdf

29. Chow SC, Shao J, Wang H. *Sample Size Calculations in Clinical Research*. 2nd Ed. Chapman & Hall/CRC. 2008. Section 3.6.2

SUPPLEMENTS/APPENDICES

Appendix A – Schedule of Procedures/Evaluations

(Please see next page)

Visit Window		Up to 10 business days after screening[1]	Within 24 business hours ** +/- three (3) business days*						As needed
**Visit**	**Screen**	**Enrollment / Baseline**	**20 observable doses ** **	**Cross-over Part 1 completion Visit ***	**20 observable doses**	**Cross-over Part 2 completion Visit***	**WK 9 to Completion of treatment**	**Study Termination / Treatment Completion**	**Early Termination/ Withdrawal**
Informed Consent / HIPAA		X							
Inclusion/Exclusion	X	X							
Demographics	X	X							
Contact Information		X							
Medical History		X							
Social History		X							
Self-efficacy to Adhere to Treatment		X							
Randomization		X							
Provision/Documentation of eDOT Device		X							
Documentation of DOT method		X		X		X	X		
Adherence to each medication dose			X		X		X		
Reasons for non-adherence to medication doses			X		X		X		
Technical difficulties associated with eDOT			X		X		X		
Rescheduled DOT visits			X		X		X		
Medication side effects			X	X	X	X	X	X	
Patient Perceptions (Includes perceptions specific to: quality of care, support of healthcare team, patient-provider rapport, satisfaction with DOT methods						X			
Document Smear and Culture Conversion Results						X			
Treatment Outcome								X	
Study withdrawal									X

Notes:

1 In the event an enrollment / baseline visit occurs more than 10 business days after initial screening, the screening should be repeated to ensure the patient still meets eligibility requirements

Appendix B – Figure 1: Illustration of screening, enrollment, study-related activities

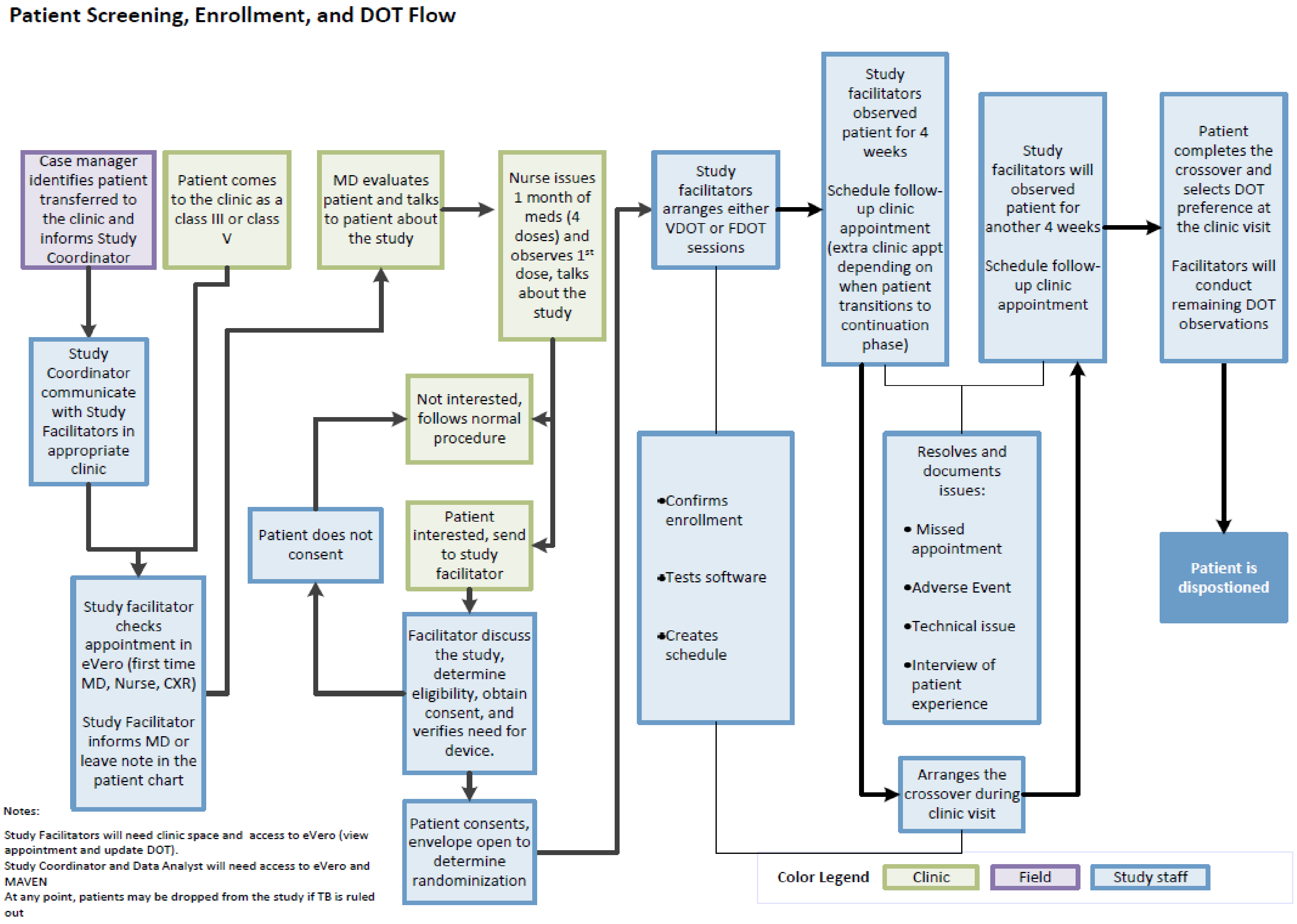

o

Appendix C – Abbreviations

Abbreviation	Full-length identification
AR	Antibiotic Resistance
BTBC	Bureau of Tuberculosis Control
CARB	Combatting Antibiotic Resistant Bacteria
CDC	Centers for Disease Control and Prevention (United States)
CRF	Case Report Form – paper or electronic questionnaires used to collect data
DOHMH	Department of Health and Mental Hygiene
DOT	Directly observed therapy
EMB	Ethambutol
eDOT	Electronic Directly Observed Therapy – in which doses of anti-TB drugs are ingested by patients, treatment adherence is monitored by TB program staff using electronic communication methods (this includes eDOT conducted in real time or with the use of recorded videos).
eDOT-live	Electronic Directly Observed Therapy conducted “Live” or in “real time”
eDOT-recorded	Electronic Directly Observed Therapy that has been recorded so that it may be viewed at a later date/time.
EMB	Ethambutol
EMR	Electronic Medical Record
ETH	Ethionamide
Digital Clinic	The NYC DOHMH BTBC’s electronic medical record system that captures demographic and provider information, clinical encounters and treatment adherence/monitoring.
HIPAA	Health Insurance Portability and Accountability
INH	Isoniazid
ipDOT	In-person Directly Observed Therapy, in which doses of anti-TB drugs are ingested by patients in the presence of TB program staff who are trained to monitor treatment adherence
IRB	Institutional review board
MAVEN	The NYC DOHMH BTBC’s TB registry used for case management activities and TB surveillance. Data is entered in “real time” into this system, which contains up to 2,400 variables. Effectively, the data in the MAVEN system is the patient’s public health record. In the event data entered into Digital Clinic is discordant with data in MAVEN, the data in

	MAVEN is considered the primary source.
MDR	Multidrug-resistant
Mtb	Mycobacterium tuberculosis
NYC	New York City
OFX	Ofloxacin
PAS	Para-aminosalicylic Acid
PZA	Pyrazinamide
PUA	Phone Use Agreement
RIF	Rifampin (alternative term is rifampicin)
TB	Tuberculosis
WHO	World Health Organization
XDR	Extensively drug resistant

APPENDIX D: LIST of FORMS and MATERIALS

Form Number	Form
**1.**	Informed Consent Form
**2.**	Assent Form
**3.**	Authorization for Use/Disclosure of Protected Health Information for Research Form
**4.**	Screening Form
**5.**	Enrollment and Baseline Visit Form
**6.**	Self-Efficacy to Adhere to Treatment – Patient Questionnaire
**7.**	Cross-over Part 1 Completion Visit Form
**8.**	Cross-over Part 2 Completion Visit Form
**9.**	Monitoring Medication Side Effects Form
**10.**	Patient Opinion Questionnaire
**11.**	Study Termination / Treatment Completion Form
	**Additional Materials**
**1.**	Master Randomization List
**2.**	<u>Randomization Envelope Label</u>
**3.**	<u>Randomization Assignment Insert</u>
**4.**	Master Participant List
**5.**	Decline Log
**6.**	Study file checklist
**7.**	Facilitator's Guide for the Focus Group Discussion
**8.**	Consent form to participate in the focus group
**9.**	Dose DOT Outcome Form (paper back-up for Study MS Access Database)
**10.**	Sputum Culture Conversion Documentation Form<br>(paper back-up for Study MS Access Database)

Appendix E – Consort Flow Diagram

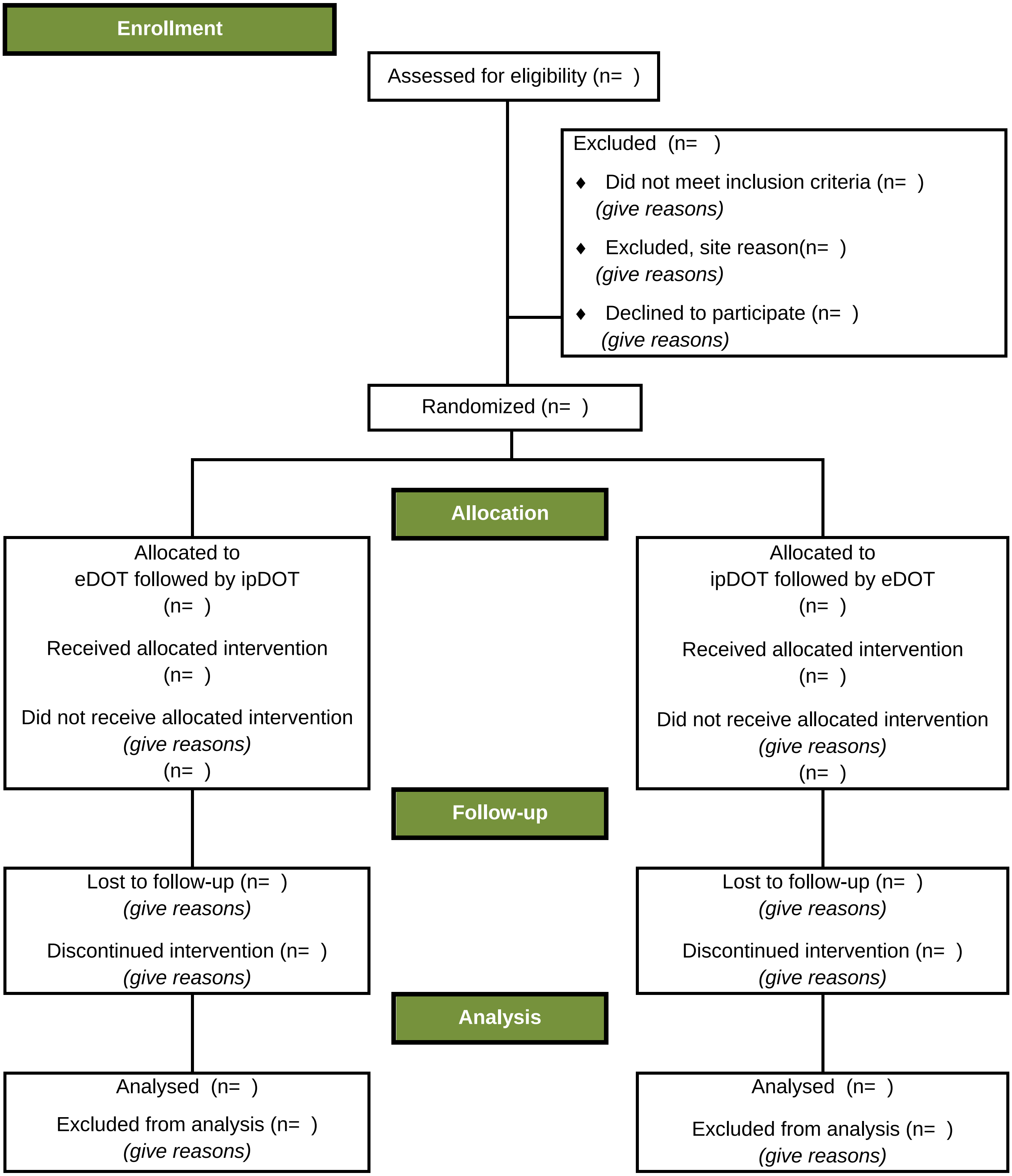

Potential Reasons for Discontinuation:
Medication side effects, withdrew consent, no longer met study criteria, poor or non-adherence, death

Appendix F – Consent and Assent Forms

Patient Consent for Participation in the Research Study - For Persons 18 Years of Age and Older

Assent to Participate in the Research Study - For Persons 17 Years of Age and Younger

Parent / Guardian Consent for Child to Act as a Research Participant

Consent for Focus Group Participation - Persons 18 Years of Age and Older

Authorization for Use/Disclosure of Protected Health Information for Research

An evaluation of traditional directly observed therapy (DOT) and electronic forms of DOT

Patient Consent for Participation in the Research Study - For Persons 18 Years of Age and Older

---

INTRODUCTION.

We are asking you to take part in a clinical trial (a type of research study) called the “eDOT Study.” You are being asked to be in the eDOT study because you are being treated for an illness called tuberculosis (TB).

During standard treatment for TB, healthcare workers meet with patients and watch as they take their TB pills. This is called In-Person Directly Observed Therapy (ipDOT).

This study will test a new way for healthcare workers to watch as patients take their TB pills, called Electronic Directly Observed Therapy (eDOT). During eDOT, patients use videos to show their healthcare worker they have swallowed their TB pills.

The videos can be done two different ways. The first is called “eDOT-recorded.” eDOT-recorded lets patients use a cell phone application to record a short video of themselves taking their TB pills and saying if they are having problems with the pills. Healthcare workers watch the videos at a later time. The second is called “eDOT-live.” eDOT-live lets patients see and talk with a healthcare worker during a video conference call on a cell phone. During the call, the patient swallows their pills while a healthcare worker watches.

All patients will take the medicine prescribed by their doctor no matter what DOT method they use. Also, all patients who are in the study or not in the study will have regular visits with the TB doctor in the clinic.

It is important to know that clinical trials only include patients who want to take part. The next pages have more information about this study. Please ask the study staff any questions you have about the study. You can ask now or at any time during the study. Please take time to make your decision. If you would like to talk with your family and friends, please do.

The eDOT study is being done in a partnership between the New York City Department of Health and Mental Hygiene, Columbia University and the Centers for Disease Control and Prevention (CDC).

WHY IS THIS STUDY BEING DONE?

This study will evaluate if patients who use eDOT complete TB treatment as often as patients who take treatment with ipDOT.

This study will examine how happy patients are with the care they receive during eDOT compared to ipDOT.

Plus, this study will examine how well the healthcare team can respond to trouble patients may have with their TB pills when patients receive care using eDOT compared to ipDOT.

WHAT WILL HAPPEN IF I AGREE TO BE IN THE STUDY?

All TB treatment will be prescribed by your doctor or nurse at the TB Chest Clinic. The study will not provide treatment or change the treatment you receive. The study will only change which method of DOT is used to watch you take your pills.

This study has two parts. During the first part, you will be placed into one of two groups.

Group 1: 20 doses of your pills using eDOT followed by 20 doses using ipDOT.

OR

Group 2: 20 doses of your pills using ipDOT followed by 20 doses using eDOT.

A healthcare worker will watch you take all of your pills.

The group you are assigned will be by chance – like flipping a coin. You and the study staff cannot choose if you will start with ipDOT or start with eDOT.

You and a member of your healthcare team will discuss the two types of eDOT videos and decide together which video type would be best for you. Please note: Only a limited number of people can use the recorded video system at one time. For this reason the staff may ask you to use the live video system.

The second part of the study starts after you take the 40 doses of medicine, and will continue until you complete your TB treatment. During this part of the study, you will be told which types of DOT are available and asked to choose one type for the rest of your treatment. The amount of time it takes you to complete treatment will depend on how many doses of medicine your doctor or nurse prescribes for you. Being in the study will not change how many doses of medicine you will take.

Patients in all groups will be asked to answer questions about themselves. These questions will ask about your ability to take your treatment and about any problems you may have. You will also be asked which method of DOT you like best and why. We will ask you to share your opinions about the care you have gotten, and we will record the outcomes of your treatment.

Some of these questions will be asked during conversations with study staff or on forms the staff will ask you to fill out. If you need help filling out the forms, the staff will be happy to help you.

We will be using your electronic medical records to collect information on the type of medicine you are taking and how often you took this medication. We will also be collecting information on any side effects you have to the medication and any symptoms experience while you are (your child is) being treated for TB.

WHAT ARE THE RISKS OF THE eDOT STUDY?

Joining this study may involve some risks or discomforts listed below.

1. You may feel some of the questions we ask are too private. You do not have to answer any study question that you do not want to.
2. If people outside of the study learn you have TB, this could change the way they treat you. To protect your privacy, we will use a study ID number instead of your name on all of the study paperwork. There will be a paper file and a computer file that will list your name, contact information and study ID number. These files will be kept in a locked cabinet, separate from other study records.
3. Even though we will do our best to keep the videos private, it is possible that someone who should not have this information may see it. If this happens you may feel embarrassed or uncomfortable. To protect your privacy, the videos can only be seen using a secure, encrypted website. Only the study staff and TB program staff will be allowed to use the website to see the videos.
4. There may also be risks that are unknown at this time. You will be given more information if other risks are found.

BENEFITS.

There may or may not be any direct benefit to you from this study. Patients who take part in this study will receive the same medical treatment as patients who are not in the study. Patients who are in this study will experience both ipDOT and eDOT. They will be able to choose the method that works best for them for the rest of their treatment.

By taking part in this study, you will help us learn more about how well eDOT works. This can help other people like you in the future.

HOW MANY PEOPLE WILL TAKE PART IN THIS STUDY?

About 300 people will be in this study over 2 years.

HOW LONG WILL MY PARTICIPATION IN THE STUDY BE?

In the first part of the study, the time to take the 40 doses of medicine will depend on your treatment. If your doctor prescribes pills each day of the week, you will take all 40 doses in about 8 weeks. If your doctor prescribes pills 3 times each week, you will take all 40 doses in about 13 weeks.

The second part will depend on how many more doses of medicine you need to take to finish your treatment. For most people this will be 16 to 18 weeks. If you took some doses of medicine before you entered this study, this second part may be shorter than 16 weeks.

CONFIDENTIALITY.

We will keep your records safe and private by making sure only approved study staff can see your records. Your name will not be used on study records. Instead you will be given a study number and this number will be placed on study records.

A paper record and computer record will link your name to the study number. The file containing your name and study number and all other paper records will be in locked cabinets. All computer records will need a password to see them.

The systems used for videos will also follow laws to keep your videos safe and private. Video files will be encrypted so the videos cannot be viewed on the phone. When the videos are sent to the Department of Health, they will only be seen by study staff and TB program staff using a password protected website.

We will not use your name in any talk or paper about the eDOT study. We will not send your name to the CDC.

We will keep all information from your Study Records private as much as the law allows. Staff from the New York City Department of Health and Mental Hygiene, Columbia University, and CDC who make sure studies follow the rules and laws for research may look at your study records.

COSTS AND PAYMENT FOR BEING IN THE STUDY.

There is no cost to you for being in the study. You will receive a $50 gift card after joining the study and answering study questions. You will receive another $50 gift card after you complete the evaluation at the end of the 40 doses of medicine with assigned DOT therapy.

If you use your cell phone to take videos when you swallow your pill, you will receive $10 a month for each month you use your phone. This will pay for your data usage while in this study.

If you do not have a cell phone that can take videos, we will loan you a phone during this study. If you borrow a phone, you do not have to pay to use this phone as long as it is used for the study. If you use the phone for reasons not related to your TB treatment, you may be removed from the study.

IN CASE OF INJURY.

If you are injured or experience harmful side effects as a result of this study, we will arrange emergency care for you. If you have insurance, we will ask you if we can bill your insurance company for your emergency care. If you have no insurance, the emergency care will be free. If you need long-term medical care for study related injuries, we will refer you to an appropriate medical care provider. The New York City Department of Health and Mental Hygiene, Columbia University, and CDC do not normally provide long-term care or compensation for an injury. Signing this form does not mean that you are giving up any legal rights.

RIGHT TO REFUSE AND REASONS FOR WITHDRAWAL.

Whether or not you take part in this study is your choice. If you decide not to take part, it will not change your regular medical care. You may quit the study at any time. Your doctor or nurse can also remove you from the study if he or she feels that it is best for your health. Your doctor or nurse will discuss this with you. We will tell you if we find information that might change your mind about the study.

ALTERNATIVE TREATMENT.

If you decide you do not want to be in this study, your decision will not change your ability to get care and treatment now or in the future from the New York Department of Health and Mental Hygiene.

If you are not in this study you will still receive DOT to monitor your treatment. This DOT may be done in-person at the clinic, a location where the program staff can meet with you, or by eDOT according to the policies of the New York Department of Health and Mental Hygiene.

PERSONS TO CONTACT.

If you have questions about the eDOT study, contact Dr. Joseph Burzynski at 347-396-7557 or Dr. Neil Schluger at 212-368-4500. If you have questions about your rights as a research participant, contact the Institutional Review Board by calling 347-396-6118. The Institutional Review Board is a group that oversees the rights and welfare of research participants.

You will be given a copy of this form to keep for your records.

CONSENT STATEMENT.

My signature below indicates that I agree to be in the eDOT study. I was given a chance to ask questions. I feel that my questions have been answered. I know that being in this study is my choice. I know that after choosing to be in this study, I may quit at any time.

Signature of participant: ________________________________ Date: ___________

Signature of person obtaining consent: ____________________ Date: ___________

An evaluation of traditional directly observed therapy (DOT) and electronic forms of DOT

Assent to Participate in the Research Study - For Persons 17 Years of Age and Younger

---

These are some things we want you to know about research studies.

We are asking you to be in a research study. Research is a way to test new ideas. Research helps us learn new things. The things we learn can help other people like you.

To be part of this research study is your choice. You can say Yes or No. Whatever you decide is OK.

Why am I being asked to be in this research study?

The name of the research study is the "eDOT study".

You are being asked to be in the eDOT study because you are being treated for an illness called tuberculosis (TB).

What is the study about?

During treatment for TB, staff from this clinic meet with you and watch as you swallow your medicine. This is called In-Person Directly Observed Therapy (ipDOT).

The eDOT study will test a new way for the staff to watch as people take their medicine. This new way is called Electronic Directly Observed Therapy (eDOT). During eDOT, people use cell phones to make videos to show the staff they have swallowed their medicine. During the videos people can also tell the staff if they are having problems with their medicine.

This study will help the doctors learn if people who use eDOT finish TB treatment as often as people who take treatment with ipDOT. The doctors also want to know how happy people are with the care they receive during the study. Plus doctors what to know how well care is provided with eDOT.

What will happen during this study?

This study will be done in two parts. During this part of the study you take 20 doses of your medicine with ipDOT. Then you will take another 20 doses of your medicine using eDOT. The time to take all 40 doses will depend on how often your doctor wants you to take medicine each week.

You cannot choose if you will start with ipDOT or eDOT. Which type of DOT you start with will be chosen by chance – like flipping a coin.

When you use a cell phone to take a video, you might talk to the clinic staff during a video phone call. Or, you might make a video as you swallow your medicine, and then send the video for the clinic staff to look at later.

You and a member of your healthcare team will discuss the two types of eDOT videos and decide together which video type would be best for you. It is important to know that a limited number of people can use the recorded video system at one time. For this reason the staff may ask you to use eDOT-live.

After you take the 40 doses of medicine, you will enter the second part of the study. During this part, you will choose the type of DOT you would like to use to finish taking your treatment.

You will also be asked to answer questions about taking your medicine. Some questions will ask what you like or do not like about using videos. Some questions will ask about your visits with clinic staff when you take your medicine. Some of these questions will be asked while you talk with the study staff. Some questions will be on forms the staff will ask you to fill out. If you need help filling out the forms, the staff will help you.

We will be looking at your records see what kind of medicine you are taking and how often you take them. Also, we will see if the medicine ever made you feel bad or if you got sicker when you were taking the medicine.

Will the study hurt?

No, the only thing that this study will do is try different ways to watch you take your medicine.

What else should I know about the study?

If you feel sick or afraid that something is wrong, talk to an adult right away.

During the first part of this study, the time you visit with the staff and the time you use a cell phone to video as you take your medicine is decided by the study. It is only after the 40 doses of medicine, in the first part of the study that you get to decide how you want staff to watch you take your medicine.

You do not have to answer any study questions that you do not want to answer.

What are the good things that might happen?

People may have good things happen to them because they are in a study. These are called "benefits." You may or may not benefit from joining this study. People who are in this study will be able to try both in-person DOT and eDOT. At the end of 40 doses of medicine you can choose the type of DOT you like best and use this until you finish your medicines.

What else might happen?

People may have other things happen to them because they are in a study. These are called "risks." You may or may not go through the following risks.

1) If people outside of the study learn you have TB, this could change the way they treat you.
2) It is possible someone might see a video of you taking your TB medicine. We do have ways to protect the videos from being seen.

3) During the first part of the study you can be put into a group that will use “live” or “recorded” videos. The group that you are put into may not be the one you want to be in. Also, the two types of video may not be equal to each other.

4) There may be risks we do not know about now. If any other risks are found, we will tell you.

Will other people know I am in this study?

We will keep your records safe and private. The records will be kept in locked cabinets and in computer databases that only study staff can open.

What if I don’t want to be in this study?

You do not have to be in the study if you do not want to. The doctors and nurses will still take care of your tuberculosis. Also, you can continue to get your medical care at this Chest Clinic.

Do I have to stay in the study?

No, you do not have to stay in the study. Even if you say yes now, you can change your mind later. It is up to you. No one in the clinic will be mad if you do not want to do this.

Will I get anything for being in the study?

You will be given a $50 gift card after joining the study and answering questions. You will be given another $50 gift card at the end of the 40 doses of medicine with assigned DOT.

If you use your cell phone to take videos when you take your medicine, you will be given $10 each month you use your phone. This will pay for your data usage while in this study.

If you do not have a cell phone that can take videos, you can borrow a phone during this study. If you borrow a phone, you do not have to pay to use this phone as long as it is used for the study. If you use the phone for reasons other than your TB treatment you could be removed from the study.

Who should I ask if I have any questions?

If you have any questions about this study, you or your parents can call Dr. Joseph Burzynski at 347-396-7557, Dr. Neil Schluger at 212-368-4500,or Diana Wong at 347-396-6118.

You will be given a copy of this form to keep.

Signatures

Before deciding if you want to be in the study, ask any questions you have. You can also ask questions during the time you are in the study.

If you sign your name below, it means that you agree to take part in this study.

Your Name: ________________________________ Your Age: ______ Date: ___________

Signature of person obtaining consent: _________________________ Date: ___________

Signature of witness: _______________________________________ Date: ___________

An evaluation of traditional directly observed therapy (DOT) and electronic forms of DOT

Parent Consent for Child to Act as a Research Participant

INTRODUCTION.

We are asking your child to take part in a clinical trial (a type of research study) called the "eDOT Study." Your child is being asked to be in the eDOT study because they are being treated for an illness called tuberculosis (TB).

During standard treatment for TB, healthcare workers meet with patients and watch as they take their TB pills. This is called In-Person Directly Observed Therapy (ipDOT).

This study will test a new way for healthcare workers to watch as patients take their TB pills, called Electronic Directly Observed Therapy (eDOT). During eDOT, patients use videos to show their healthcare worker they have swallowed their TB pills.

The videos can be done two different ways. The first is called "eDOT-recorded." eDOT-recorded lets patients use a cell phone application to record a short video of themselves taking their TB pills and saying if they are having problems with the pills. Healthcare workers watch the videos at a later time. The second is called "eDOT-live." eDOT-live lets patients see and talk with a healthcare worker during a video conference call on a cell phone. During the call, the patient swallows their pills while a healthcare worker watches.

All patients will take the medicine prescribed by their doctor no matter what DOT method they use. Also, all patients who are in the study or not in the study will have regular visits with the TB doctor in the clinic.

It is important to know that clinical trials only include patients who want to take part. The next pages have more information about this study. Please ask the study staff any questions you have about the study. You can ask now or at any time during the study. Please take time to make your decision. If you would like to talk with your family and friends, please do.

The eDOT study is being done in a partnership between the New York City Department of Health and Mental Hygiene, Columbia University and the Centers for Disease Control and Prevention (CDC).

WHY IS THIS STUDY BEING DONE?

This study will evaluate if patients who use eDOT complete TB treatment as often as patients who take treatment with ipDOT.

This study will examine how happy patients are with the care they receive during eDOT compared to ipDOT.

Plus, this study will examine how well the healthcare team can respond to trouble patients may have with their TB pills when patients receive care using eDOT compared to ipDOT.

WHAT WILL HAPPEN IF I ALLOW MY CHILD TO BE IN THE STUDY?

All TB treatment will be prescribed by your child's doctor or nurse at the TB Chest Clinic. The study will not provide treatment or change the treatment your child receives. The study will only change which method of DOT is used to watch your child take their pills.

This study has two parts. During the first part, your child will be placed into one of two groups.

<u>Group 1:</u> 20 doses of pills using eDOT followed by 20 doses using ipDOT.

OR

<u>Group 2:</u> 20 doses of pills using ipDOT followed by 20 doses using eDOT.

A healthcare worker will watch your child take all of their pills.

Which group your child will be assigned will be by chance – like flipping a coin. You, your child, and the study staff CANNOT choose if your child will start with ipDOT or start with eDOT.

Your child and a member of your child's healthcare team will discuss the two types of eDOT videos and decide together which video type would be best for your child. Please note: Only a limited number of people can use the recorded video system at one time. For this reason the staff may ask your child to use the live video system.

The second part of the study starts after your child takes the 40 doses of medicine and will continue until they complete their TB treatment. During this part of the study, your child will choose either in-person DOT, eDOT-live, or eDOT-recorded for the rest of their treatment. The amount of time it takes your child to complete treatment will depend on how many doses of medicine the doctor or nurse prescribes for your child. Being in the study will not change how many doses of medicine your child will take.

Patients in all groups will be asked to answer questions about themselves. These questions will ask about your child's ability to take their treatment and about any problems they may have.

Your child will also be asked which method of DOT they like best and why. We will ask your child to share their opinions about the care they have gotten. We will record the outcomes of your child's treatment. Some of these questions will be asked during conversations with study staff or on forms the staff will ask your child to fill out. If your child needs help filling out the forms, the staff will be happy to help.

We will be using your child's electronic medical records to collect information on the type of medicine they are taking and how often your child took this medication. We will also be collecting information on any side effects your child has to the medication and any symptoms experience while your child is being treated for TB.

WHAT ARE THE RISKS OF THE eDOT STUDY?

Joining this study may involve some risks or discomforts listed below.

5. Your child may feel some of the questions we ask are too private. Your child does not have to answer any study question they do not want to.
6. If people outside of the study learn your child has TB, this could change the way they treat your child. To protect your child's privacy, we will use a study ID number instead of your child's name on all of the study paperwork. There will be a paper file and a computer file that will list your child's name, contact information and study ID number. These files will be kept in a locked cabinet, separate from other study records.
7. Even though we will do our best to keep the videos private, it is possible that someone who should not have this information may see it. If this happens your child may feel embarrassed or uncomfortable. To protect your child's privacy, the videos can only be seen using a secure, encrypted website. Only the study staff and TB program staff will be allowed to use the website to see the videos.
8. There may also be risks that are unknown at this time. You and your child will be given more information if other risks are found.

BENEFITS.

There may or may not be any direct benefit to your child from this study. Patients who take part in this study will receive the same medical treatment as patients who are not in the study. Patients who are in this study will experience both ipDOT and eDOT. They will be able to choose the method that works best for them for the rest of their treatment.

By taking part in this study, your child will help us learn more about how well eDOT works. This can help other people like your child in the future.

HOW MANY PEOPLE WILL TAKE PART IN THIS STUDY?

About 300 people will be in this study over 2 years.

HOW LONG WILL MY CHILD PARTICIPATE IN THE STUDY?

In the first part of the study, the time to take the 40 doses of medicine will depend on your child's treatment. If your child's doctor prescribes pills each day of the week, your child will take all 40 doses in about 8 weeks. If your child's doctor prescribes pills 3 times each week, your child will take all 40 doses in about 13 weeks.

The second part will depend on how many more doses of medicine your child needs to take to finish their treatment. For most people this will be 16 to 18 weeks. If your child took some doses of medicine before he or she entered this study, this second part may be shorter than 16 weeks.

CONFIDENTIALITY.

We will keep your child's records safe and private by making sure only approved study staff can see your records. Your child's name will not be used on study records. Instead your child will be given a study number and this number will be placed on study records.

A paper record and computer record will link your child's name to the study number. The file containing your child's name and study number and all other paper records will be in locked cabinets. All computer records will need a password to see them.

The systems used for videos will also follow laws to keep your child's videos safe and private. Video files will be encrypted so the videos cannot be viewed on the phone. When the videos are sent to the Department of Health, they will only be seen by study staff and TB program staff using a password protected website.

We will not use your child's name in any talk or paper about the eDOT study. We will not send your child's name to the CDC.

We will keep all information from your child's Study Records private as much as the law allows. Staff from the New York City Department of Health and Mental Hygiene, Columbia University, and CDC who make sure studies follow the rules and laws for research may look at your child's study records.

COSTS AND PAYMENT FOR BEING IN THE STUDY.

There is no cost to you if your child is in the study. Your child will receive a $50 gift card after joining the study and answering study questions. Your child will receive another $50 gift card after he or she completes the evaluation at the end of the 40 doses of medicine with assigned DOT therapy.

If your child uses a personal cell phone to take videos when they swallow their medicine, they will receive $10 a month for each month he or she uses a personal cell phone. This will pay for the data usage while in this study.

If your child does not have a cell phone that can take videos, we will loan your child a phone during this study. If your child needs to borrow a phone, there will be no cost to use this phone as long as it is used for the study. Using the phone for reasons other than their TB treatment could cause your child to be removed from the study.

IN CASE OF INJURY.

If your child is injured or experiences harmful side effects as a result of this study, we will arrange emergency care for your child. If your child has insurance, we will ask you if we can bill the insurance company for the emergency care. If your child does not have insurance, the emergency care will be free. If your child needs long-term medical care for study related injuries, we will refer your child to an appropriate medical care provider. The New York City Department of Health and Mental Hygiene, Columbia University, and CDC do not normally

provide long-term care or compensation for an injury. Signing this form does not mean that you are giving up any legal rights for your child.

RIGHT TO REFUSE AND REASONS FOR WITHDRAWAL.

Whether or not your child takes part in this study is a choice for your child. If you decide not to allow your child to take part, it will not change your child's regular medical care. Your child may quit the study at any time. Your child's doctor or nurse can also remove your child from the study if he or she feels that it is best for your child's health. Your child's doctor or nurse will discuss this with you and your child. We will tell you and your child if we find information that might change your mind about the study.

ALTERNATIVE TREATMENT.

If you decide you do not want your child to be in this study, your decision will not change your child's ability to get care and treatment now or in the future from the New York Department of Health and Mental Hygiene.

If your child is not in this study your child will still receive DOT to monitor his or her treatment. This DOT may be done in-person at the clinic, a location where the program staff can meet with your child, or by eDOT according to the policies of the New York Department of Health and Mental Hygiene.

PERSONS TO CONTACT.

If you or your child have questions about the eDOT study, contact Dr. Joseph Burzynski at 347-396-7557 or Dr. Neil Schluger at 212-368-4500. If you have questions about your rights as a research participant, contact the Institutional Review Board by calling 347-396-6118. The Institutional Review Board is a group that oversees the rights and welfare of research participants.

You will be given a copy of this form to keep for your records.

CONSENT STATEMENT.

My signature below indicates that I agree to allow my child to be in the eDOT study. I was given a chance to ask questions. I feel that my questions have been answered. I know that allowing my child to be in this study is my choice. I know that after choosing to be in this study, my child may quit at any time.

Signature of participant: ________________________________ Date: ___________

Signature of person obtaining consent: ____________________ Date: ___________

Patient Opinions about In-Person and Electronic Directly Observed Therapy

Consent for Focus Group Participation - Persons 18 Years of Age and Older

INTRODUCTION.

We are asking you to take part in a focus group about directly observed therapy (DOT). You are being asked to participate because you are undergoing treatment for tuberculosis (TB) and you have experience with DOT done in person (ipDOT) and with videos (eDOT).

A focus group is a form of research. During focus groups people are asked to talk about their thoughts, experiences, and feelings about something. Questions are asked in a group setting. People who participate talk to the organizer and with other group members. The focus group you are being asked to join will talk about ipDOT and eDOT.

The next pages have more information about this research study. Please ask the research team any questions you have about the study. You can ask now or at any time during focus group activities.

This research is being done in a partnership between the New York City Department of Health and Mental Hygiene, Columbia University and the Centers for Disease Control and Prevention (CDC).

WHY IS THE FOCUS GROUP BEING DONE?

This research will help TB program leaders gain a better understanding of patients' experiences with the two types of DOT and help identify ways to make improvements for future patients.

WHAT WILL HAPPEN IF I AGREE TO BE IN THE FOCUS GROUP?

If you decide to participate in the focus group, first you will be asked to fill out a short questionnaire that will allow the research team to describe the people who participated. For example, you will be asked your age, gender, race, and level of education. You will not be asked to write your name on the questionnaire.

Next, the focus group leader will ask the group a series of questions. These questions will ask about your experiences with ipDOT and eDOT, your opinions about the quality of care you received during ipDOT and eDOT, your satisfaction with the different methods of DOT, and suggestions for making improvements. If you do not want group members to know your name, you can use any name you choose during the group discussion.

The group discussion will be tape recorded and a member of the research team will take notes. This will allow the research team to create very detailed notes of the conversation. No one will hear the tape recordings but the research team. The tape recordings will be kept in a locked cabinet. Once the notes have been analyzed the tape recordings will be destroyed. When the final report is written, it may include quotes of what some people said, but your name will not be included in the report.

WHAT ARE THE RISKS OF BEING A PART OF THE FOCUS GROUP?

One risk of participating in a focus group is you may be uncomfortable discussing an opinion in front of others. If you feel uncomfortable, you can share your opinion privately with members of the research team at the end of the focus group. Also, you may feel some of the questions we ask are too private. You do not have to answer any question that you do not want to.

BENEFITS.

We do not expect you will benefit directly from participating in this focus group. By taking part in this research, you will help us learn more about how well eDOT works. This can help other TB patients like you in the future.

HOW MANY PEOPLE WILL TAKE PART IN THE FOCUS GROUP?

A total of 2 to 4 focus groups will be held. Between 5 and 8 people will be asked to join each focus group.

HOW MUCH TIME WILL THE FOCUS GROUP REQUIRE?

Focus group activities should take 1 ½ to 2 hours.

CONFIDENTIALITY.

Your identity will be kept confidential to the extent provided by law. Your name will not be used on any paperwork. Instead you will be given an identification number and this number will be placed on paperwork. The list connecting your name to your identification number will be kept in a locked file. Only approved research staff can see your paperwork or listen to the tape recordings.

You can use any name you choose during the group discussions. As stated above, we will tape record the discussions to help us create detailed notes of the discussion. The tape recordings will also be kept in a locked cabinet. When this research is completed and the data have been analyzed, the list and tape recordings will be destroyed. Your name will not be used in any report. We will not use your name in any talk or paper about this research. The results of this research will summarize the opinions of everyone who participated.

COSTS AND PAYMENT.

There is no cost to participate in the focus group. To thank you for your time, you will receive a gift card worth $50. In addition, you will be provided a $50 gift card to compensate you for your transportation costs.

RIGHT TO REFUSE AND WITHDRAW.

Whether or not you take part in the focus group is your choice. You can join now and quit at any time. Whatever decision you make will not affect the care you receive through the Department of Health. If you decide to participate, you can also refuse to answer questions that make you uncomfortable. There are no right or wrong answers to the questions you will be asked.

You are not waiving any of your legal rights by signing this consent form.

PERSONS TO CONTACT.

If you have questions about this research, contact Dr. Joseph Burzynski at 347-396-7557 or Dr. Neil Schluger at 212-368-4500. If you have questions about your rights as a research participant, contact the Institutional Review Board by calling 347-396-6118. The Institutional Review Board is a group that oversees the rights and welfare of research participants.

You will be given a copy of this form to keep for your records.

CONSENT STATEMENT.

My signature below indicates that I agree to participate in the focus group. I was given a chance to ask questions. I feel that my questions have been answered. I know that being in this research study is my choice. I know that after choosing to be in this research study, I may quit at any time.

Signature of participant: ________________________________ Date: ___________

Signature of person obtaining consent: ____________________ Date: ___________

AUTHORIZATION FOR USE/DISCLOSURE OF PROTECTED HEALTH INFORMATION (PHI) FOR RESEARCH

Participant Name: __________________

IRB Protocol Number: _______________

Research Protocol:
An evaluation of traditional directly observed therapy (DOT) and electronic forms of DOT for Tuberculosis (TB) treatment

Principal Investigator: Joseph Burzynski, MD, MPH and Michelle Macaraig, DrPH, MPH

Sponsor: U.S. Centers for Disease Control and Prevention

What is the purpose of this form?

You are being asked to sign this form so that the New York City Department of Department of Health and Mental Hygiene Bureau of Tuberculosis Control (NYC DOHMH BTBC) may use and release your protected health information for research.

Why do the researchers want my protected health information?

The researchers want to use your protected health information as part of the research protocol listed above and as described to you in the informed consent.

What protected health information do the researchers want to use?

All medical information, including but not limited to information and/or records of any diagnosis or treatment of disease or condition, which may include sexually transmitted diseases (e.g., HIV, etc.) or communicable diseases, history of drug or alcohol dependency, etc.; personal identifiers, including but not limited to your name, medical record number, date of birth, dates of service, etc.; any past, present, and future history, examinations, laboratory results, imaging studies and reports and treatments of whatever kind, and any other information related to or collected for use in the research protocol, regardless of whether the information was collected for research or non-research (e.g., treatment) purposes.

Who will disclose, use and/or receive my protected health information?

All Individuals/entities listed in the informed consent documents, including but not limited to, the physicians, nurses and staff and others performing services related to the research (whether at NYC DOHMH BTBC or elsewhere); the sponsor of the research and its employees and agents; and any outside regulatory agencies providing oversight or performing other legal and/or regulatory functions for which access to participant information is required.

How will my protected health information be protected once it is given to others?

The research information that is shared with people outside of the NYC DOHMH BTBC will not include your name, address, telephone number or any other direct identifiers unless disclosure of the information is required by law or you have authorized the disclosure.

How long will this Authorization last?
Your authorization to use and share the information collected for this research purpose will expire when the research is completed.

Can I cancel this Authorization?
You may cancel this Authorization at any time by notifying the Principal Investigator, in writing, referencing the research protocol and IRB Protocol Number. If you cancel this Authorization, the study doctor and staff will not use any new health information for research. However, researchers may continue to use the protected health information that was provided before you cancelled your authorization.

Can I see my protected health information?
You have a right to request to see your protected health information. However, to ensure the scientific integrity of the research, you will not be able to review the research information until after the research protocol has been completed.

Signature of participant: ____________________________________ Date: _______

or participant's legally authorized representative: __________________________________

Printed Name of participant’s representative: _______________________ Date: ________

Relationship to the participant: ____
