# Supplementary material for: In-Person vs Electronic Directly Observed Therapy for Tuberculosis Treatment Adherence: A Randomized Noninferiority Trial
Source: JAMA Netw Open. 2022 Jan 20;5(1):e2144210. doi: 10.1001/jamanetworkopen.2021.44210 (PMC8777548; doi:10.1001/jamanetworkopen.2021.44210)
Supplement: Supplement 2. — eAppendix 1. Statistical Details and Auxiliary Analyses eAppendix 2. Noninferiority of eDOT Between Seasons eTable 1. Dose Outcome Analysis With Percentage Differences eTable 2. Reasons for Study Exclusion or Declined Enrollment Among Persons Who Met Initial Screening Criteria eTable 3. Characteristics of Enrolled Participants by Randomization Assignment eTable 4. Model-adjusted Percentage Differences Between eDOT vs. ipDOT Estimated With Bootstrap Logistic GLMM, by Season (Quarter) eFigure. Dose Outcome Completed [file jamanetwopen-e2144210-s002.pdf]

Supplemental Online Content

Burzynski J, Mangan JM, Lam CK, et al; eDOT Study Team. In-person vs electronic directly observed therapy for tuberculosis treatment adherence: a randomized noninferiority trial. *JAMA Netw Open.* 2022;5(1):e2144210. doi:10.1001/jamanetworkopen.2021.44210

eAppendix 1. Statistical Details and Auxiliary Analyses

eAppendix 2. Noninferiority of eDOT Between Seasons

eTable 1. Dose Outcome Analysis With Percentage Differences

eTable 2. Reasons for Study Exclusion or Declined Enrollment Among Persons Who Met Initial Screening Criteria

eTable 3. Characteristics of Enrolled Participants by Randomization Assignment

eTable 4. Model-adjusted Percentage Differences Between eDOT vs. ipDOT Estimated With Bootstrap Logistic GLMM, by Season (Quarter)

eFigure. Dose Outcome Completed

This supplemental material has been provided by the authors to give readers additional information about their work.

© 2022 Burzynski J et al. *JAMA Network Open.*

eAppendix 1. Statistical Details and Auxiliary Analyses

Logistic GLMM — Carryover Effects

Expanding on the description in Methods, the logistic generalized linear mixed model (GLMM)[1] included fixed effect predictors representing DOT method, participant randomization group, crossover period, season (represented as calendar quarter), the interaction between DOT method and season, and the dose outcome during each of the two-preceding scheduled-and-observable doses. These preceding dose outcomes represent "carryover" or "lagged" effects that are a consequence of correlations that arise between serial observations. Each lagged dose outcome included in the model, however, eliminates from statistical analysis one observation at the beginning of the series because the lagged outcome must be missing in the eliminated observation. For the present analysis, use of two lagged doses eliminated two observations at the beginning of each crossover period for each participant. A lag of two doses was selected to balance the desire to account for carryover effects against the attendant sacrifice of observations.

All statistical analyses and calculations were conducted with the SAS software application.[2]

Non-Inferiority Testing and Bootstrap Estimation
In order to evaluate this study's primary hypothesis that the proportion of scheduled-and-observable doses completed under eDOT were non-inferior to the proportion of those completed under ipDOT, the percentage-difference in dose outcome between ipDOT vs. eDOT was formulated as the difference in the least-square means of the percentage-dose outcome of each DOT method obtained from the logistic GLMMs:

$$\Delta\% = percentageOutcome_{ipDOT} - percentageOutcome_{eDOT}$$

The 95 percent confidence interval of the percentage-difference was obtained with robust "bootstrap" estimation[3] implemented with the logistic GLMM, which was run for 1,000 replicate datasets created by random sampling with replacement of participant data from the original dataset and stratified by study randomization group. The total number of participants in each replicate dataset was the same as in the original dataset. A percentage-difference was calculated as described above for each replicate logistic GLMM, and the median of the 1,000 percentage-differences represented the bootstrap estimate of the percentage difference, and the 2.5th and 97.5th percentiles represent the upper and lower bounds respectively of the bootstrap 95 percent confidence interval. The primary hypothesis was tested by comparing the upper bootstrap confidence limit of the percentage-difference with the designated +10.00 percent non-inferiority limit, where an upper bootstrap confidence limit less-than-or-equal to +10.00 percent indicated non-inferiority.

Evaluation of "Modified" Intention-to-Treat Analytic Mode
The logistic GLMM was run in four analytic modes to compare different representations of the DOT method used at each dose. One mode —designated "modified" intention-to-treat ("mITT") — represents DOT method at each dose in accordance with the participant's randomization assignment, regardless of the DOT mode the participant actually undertook for that dose. This is the conventional representation for ITT analysis in randomized controlled trials, but the implementation reported in the main text was designated "modified" because ITT analysis is typically run on all participants enrolled in a study, whereas our mITT analysis was restricted to a subset of participants (N = 173) who had completed Crossover Periods 1 and 2. To evaluate the potential effect on the mITT results of using the subset of 173 participants, the logistic GLMM was run in a conventional ITT analytic mode (designated "ITT") with 33 additional enrolled participants (N = 206) who did not complete both Crossover Periods, but otherwise had sufficiently many observations to run the logistic GLMM with lags. Among the total 216 enrolled participants, however, five participants were excluded who had no data because they withdrew before the start of the crossover periods, and another five were excluded because they did not have sufficient data to represent carry-over effects required for the logistic GLMM. The 206 participants in the ITT analysis, therefore, comprise all enrolled participants for which analysis with the logistic GLMM is possible, and is thereby the configuration closest to a typical ITT analysis attainable with these data.

In both the mITT and ITT analytic modes (eTable 1), eDOT was found to be non-inferior to ipDOT at a 95% confidence level. In addition, the mITT bootstrap percentage-difference of −2.56% [95%bsCI: −4.80, −0.28] was nearly equivalent to the ITT percentage-difference of −2.03% [95%bsCI: −4.48, 0.26].

© 2022 Burzynski J et al. *JAMA Network Open.*

Non-Inferiority Testing with Naïve Univariate Methods

The logistic GLMM reported herein aimed to estimate the percentage-difference and variance in doses completed under ipDOT and eDOT in order to test the hypothesis of non-inferiority, while the mixed effects approach the GLMM embodies adjusted for biases expected to arise from correlations inherent in the study design. This approach minimizes bias and maximizes precision in evaluation of the primary hypothesis and is therefore the method of choice in this context [4]. The demonstrable power of the GLMM notwithstanding, its complexity may obscure the essential difference in dose outcome performance of eDOT vs. ipDOT. In order to reduce this difference to its simplest terms, while also acknowledging the increase in bias, non-inferiority was evaluated with two naïve univariate analyses that compare the difference in doses completed only with respect to DOT method, without further adjustment for fixed effect predictors or correlations arising from the study design (eTable 1). The univariate analyses were run in the ITT analytic mode with 211 enrolled participants (five of the 216 enrolled participants were excluded because they withdrew before the start of the crossover periods).

The first naïve univariate analysis ("Naïve Univariate — By Dose") is based on dose frequencies in a 2 × 2 table of dose completion vs. DOT method, and the 95% confidence interval of the percentage-difference between DOT methods was calculated according to Agresti-Caffo [A-C][5]. The upper confidence limit was less than the +10.00 percent non-inferiority limit, consistent with the conclusion that eDOT is non-inferior to ipDOT at a 95% confidence level. The univariate percentage-difference (−1.12%) was less than its respective estimate from the logistic GLMMs (N = 173; ITT: −2.56%).

In the second naïve univariate analysis ("Naïve Univariate — By Participant"), the unit of observation is participant, where the percentage of doses completed was calculated by DOT method for each participant. The percentage-difference between DOT methods and its 95% confidence interval were estimated with the bootstrap, and the upper bootstrap confidence limit was less than +10.00 percent, consistent with the conclusion that eDOT is non-inferior to ipDOT at a 95% confidence level. The magnitude of the univariate percentage-differences for ITT (−1.12%) and EMP (−1.66%) were close to those for the first naïve univariate analysis.

© 2022 Burzynski J et al. *JAMA Network Open.*

eAppendix 2. Noninferiority of eDOT Between Seasons

The greater New York City area is highly urbanized and located in a temperate climate zone of North America. In view of the general demands of directly observed therapy, an analysis was conducted to assess whether seasonal conditions would influence the dose outcome differently between DOT methods, possibly confounding tests of non-inferiority. In particular, the need for patient and staff to meet daily for in-person observation suggested that completed doses under ipDOT might have been hindered when, for example, inclement winter weather interrupted public transportation, or patients elected to take a summer vacation or travel for holidays. The influence of season on dose outcomes observed under eDOT vs. ipDOT was evaluated with an interaction term between DOT method and season included in the logistic GLMM. The interaction term enables quantification of non-inferiority by season for the modified Intention-to-Treat (mITT) and empirical (EMP) analytic modes (eTable 4). For each season, the maximum among the mITT and EMP modes of the upper 95% bootstrap confidence limit of the percentage-difference between eDOT vs. ipDOT is 4.68% (EMP) during Spring (April – June); 3.09% (EMP) during Summer (July – September); 3.78% (mITT) during Autumn (October – December); and 0.62% (EMP) during Winter (January – March). With no upper confidence limit exceeding 10% non-inferiority margin, these results indicate that in each of four seasons eDOT is non-inferior to ipDOT. In an urban area located in a temperate climate, seasonal effects did not alter the conclusion that eDOT is non-inferior to ipDOT.

© 2022 Burzynski J et al. *JAMA Network Open.*

eTable 1. Dose Outcome Analysis With Percentage Differences

Dose Outcome Analysis Mode	N, Participants	N, Doses	Percentage−Difference [95%CI]
Completed Dose: Staff observed the participant ingest the dose of medication during a DOT visit			
mITT Comparison			
Modified ITT	173	6,436	−2.56% [−4.80, −0.28]
ITT	206	7,163	−2.03% [−4.48, 0.26]
Naïve Univariate — By Dose			
ITT	211	7,225	−1.12% [−2.61, 0.37]
Naïve Univariate — By Participant			
ITT	211	7,225	−1.12% [−3.62, 1.59]

Percentage-Difference [CI] = ipDOT − eDOT
Model-Adjusted Estimates, Except Naïve Univariate
ITT: Intention-to-Treat
95%CI: bootstrap confidence interval, except Naïve Univariate — By Dose
95% confidence interval for Naïve Univariate — By Dose: Agresti-Caffo method[4]
Bootstrap Replicates: 1,000

© 2022 Burzynski J et al. *JAMA Network Open.*

eTable 2: Reasons for Study Exclusion or Declined Enrollment Among Persons Who Met Initial Screening Criteria	
**102 (12%) Individuals did not meet protocol inclusion criteria (108 documented reasons)**	
• N=23 Patient has plans to move out of the catchment areas<br>• N=22 Medical condition, that, in the investigator's or the clinic physician's judgment make study participation not in the individual's best interest<br>• N= 17 Prescribed any injectable, anti-TB medication<br>• N=12 *Other Reason*<br>• N=9 Prescribed a non-rifampin treatment regimen	• N=8 Suspected or documented tuberculosis involving the central nervous system and/or bones and/or joints, and/or miliary tuberculosis and/or pericardial tuberculosis<br>• N=8 Cognitive or physical disability that prevents full participation in electronic DOT<br>• N=4 Patient's *M. tuberculosis* isolate is already known to be resistant to rifampin<br>• N=3 Patient has demonstrated poor adherence to initial doses of anti-tuberculosis medication<br>• N=2 Patient is currently enrolled in another clinical trial
**60 (7%) Individuals not enrolled by clinic's choice (70 documented reasons)**	
• N= 17 Investigator considers patient's personal issues or situation may be a problem for treatment adherence<br>• N=11 Patient has a history of non-adherent behaviors<br>• N=8 Program staff have determined the patient's address or residence location is not readily accessible for visiting<br>• N=7 Belligerent/hostile to staff<br>• N=7 Patient does not appear to understand information presented regarding the study<br>• N= 4 Patient is not able to comply with the DOT treatment or drug regimen<br>• N=4 Patient has active, symptomatic co-morbidity requiring close medical supervision and treatment	• N=4 Patient has current, significant psychiatric condition that, in the investigator's or the clinic physician's judgment make study participation not in the individual's best interest.<br>• N=4 *Other Reason*<br>• N=2 Inconclusive lab results and/or clinical presentation of TB (for example, one positive AFB smear result and/or normal chest x-ray; no TB symptoms)<br>• N= 2 Patient has current, significant alcohol or drug abuse that, in the investigator's or the clinic physician's judgment make study participation not in the individual's best interest.
**442 (54%) Individuals declined study enrollment (981 documented reasons)**	
• N= 213 DOT is inconvenient<br>*The type of DOT considered inconvenient:*<br>o *Both clinic and community-based ipDOT (N=179, 84%)*<br>o *Both recorded and live eDOT (N=10, 5%)*<br>o *All forms of DOT (N=8, 4%)**<br>o *Clinic-based ipDOT (N= 6, 3%)*<br>o *Community-based ipDOT (N=5, 2%)*<br>o *Not specified (N=5, 2%)*<br>• N=129 Patient preferred a specific form of DOT	• N=28 Patient is too busy or has too much stress<br>• N=26 Family member against/opposes enrollment<br>• N=19 Concerns or discomfort related to the use of technology (*Patient's age was cited as part of the reason for concern/discomfort for 7 (37%) of the 19 individuals*)<br>• N=18 Concerns regarding being "experimented" upon<br>• N=17 Length and/or complexity of informed consent

Preferred form of DOT<br>○ *eDOT: Recorded-video (N=45, 35%)*<br>○ *eDOT: Live-video (N=37, 29%)*<br>○ *Community-based ipDOT (N=19, 15%)*<br>○ *Clinic-based ipDOT (N=5, 3%)*<br>○ *Self-administered therapy (N=23, 18%)**<br>• N=85 Patient does not want to be randomized<br>• N=74 Patient prefers routine care<br>• N=71 Missing work or school would be a problem<br>• N=67 Number of visits not convenient<br>• N=28 Other/Miscellaneous Reasons<br>• N=37 Worried about losing income<br>• N= 32 Does not understand languages available for translation<br>• N=32 Patient worried about study commitment / family issues<br>• N=30 Worried about enrolling in any research studies	• N=14 Patient feeling too ill or has other health problems<br>• N= 13 Does not understand study<br>• N=13 Worried about supervisor's/teacher's response to missed work/school<br>• N=9 Feels TB diagnosis is inaccurate<br>• N=6 Negative interaction with staff<br>• N=6 Does not trust information from staff<br>• N=6 Worried about impact on other medical problems or medications<br>• N=4 Moving / Traveling<br>• N=2 Has trouble keeping medical appointments in general<br>• N=1 Unable to communicate for other reasons (e.g. neurologic incapacity, etc.)<br>• N=1 Primary care or other physician's concerns about TB in this patient
* The NYC DOHMH BTBC Tuberculosis Clinical Policies and Protocols Manual notes that DOT is the standard of care in TB treatment and encouraged for all patients. Patients do have the authority to decline/refuse DOT and opt for self-administered therapy.<br>https://www1.nyc.gov/site/doh/health/health-topics/tb-hosp-manual.page	

© 2022 Burzynski J et al. *JAMA Network Open.*

eTable 3: Characteristics of Enrolled Participants by Randomization Assignment

Characteristic	All Enrolled (N= 216)	Participants Randomized to Study Group 1 ipDOT followed by eDOT N= 113 (52%)	Participants Randomized to Study Group 2 eDOT followed by ipDOT N= 103 (48%)
**Demographic Data**			
**Sex**			
Male	140 (65 %)	68 (60 %)	72 (70 %)
Female	76 (35 %)	45 (40 %)	31 (30 %)
**Median** [Min, Max] **Age in Years**	42 [16, 86]	38 [16, 86]	44 [18, 76]
**Age Group**			
16-20	10 (5 %)	6 (5 %)	4 (4 %)
21-30	57 (26 %)	35 (31 %)	22 (21 %)
31-40	32 (15 %)	18 (16 %)	14 (14 %)
41-50	38 (17 %)	20 (18 %)	18 (17 %)
51-60	41 (19 %)	20 (18 %)	21 (20 %)
61-70	21 (10 %)	8 (7 %)	13 (13 %)
71-80	15 (7 %)	4 (3 %)	11 (11 %)
81-90	2 (1 %)	2 (2 %)	
**Country of Birth**			
US born	27 (13 %)	11 (10 %)	16 (16 %)
Non-US born	187 (87 %)	102 (90 %)	85 (82 %)
Unknown / Missing	2 (1 %)	0	2 (2 %)
**Global Region of Birth**			
Africa	18 (8 %)	8 (7 %)	10 (10 %)
Asia	84 (39 %)	40 (35 %)	44 (43 %)
Caribbean	31 (14 %)	16 (14 %)	15 (14 %)
Central America	6 (3 %)	4 (3.5 %)	2 (2 %)
Europe	7 (3 %)	4 (3.5 %)	3 (3 %)
North America	39 (18 %)	22 (20 %)	17 (16 %)
South America	29 (13 %)	19 (17 %)	10 (10 %)
Unknown / Missing	2 (1 %)	0	2 (2 %)
**Race / Ethnicity***			
African American / Black, Non-Hispanic	43 (20 %)	20 (18 %)	23 (22 %)
Asian/ Pacific Islander/ Hawaiian	80 (37 %)	37 (33 %)	43 (42 %)
Hispanic	71 (33 %)	43 (38 %)	28 (27 %)
Other / Multiple**	13 (6 %)	9 (8 %)	4 (4 %)
White, Non-Hispanic	9 (4 %)	4 (3 %)	5 (5 %)
**Access to Video Device Prior to Enrollment**			
Yes	149 (69 %)	78 (69 %)	71 (69 %)
No	67 (31 %)	35 (31 %)	32 (31 %)
**Social and Medical History**			
**Primary Language Spoken**			
English	55 (25 %)	29 (26 %)	26 (25 %)
Spanish	56 (26 %)	34 (30 %)	22 (21 %)

© 2022 Burzynski J et al. *JAMA Network Open.*

**Characteristic**	All Enrolled (N= 216)	Participants Randomized to Study Group 1 ipDOT followed by eDOT N= 113 (52%)	Participants Randomized to Study Group 2 eDOT followed by ipDOT N= 103 (48%)
Chinese (Cantonese, Fujianese, Mandarin)	24 (11 %)	9 (8 %)	15 (15 %)
French, Creole, Pidgins, French-based Other	16 (7 %)	9 (8 %)	7 (7 %)
Other	60 (28 %)	30 (26 %)	30 (29 %)
Unknown	5 (2%)	2 (2 %)	3 (3 %)
**Educational Attainment**			
No formal schooling	12 (6 %)	8 (7 %)	4 (4 %)
Primary school (Grades 1-5)	9 (4 %)	4 (4 %)	5 (5 %)
Middle school (Grades 6-8)	27 (13 %)	14 (12 %)	13 (12 %)
Secondary school (Grades 9-12)	84 (39 %)	40 (35 %)	44 (43 %)
College+	62 (29 %)	34 (30 %)	28 (27 %)
Unknown / Refused to Answer	22 (10 %)	13 (12 %)	9 (9 %)
**TB Disease, Pulmonary** (Yes)	190 (88 %)	100 (89 %)	90 (87 %)

*To assess whether participants were similar across analytic groups, participants' race and ethnicity were obtained from clinic records.
** Race: Other/Multiple denotes persons who identified as a combination two or more fixed race and ethnicity categories.

© 2022 Burzynski J et al. *JAMA Network Open.*

eTable 4: Model-adjusted percentage-differences between eDOT vs. ipDOT estimated with bootstrap logistic GLMM, by season (quarter)		
	**Analysis Mode**	
	**Modified ITT**<br>**N = 173**	**Empirical**<br>**N = 173**
Spring: April - June		
ipDOT Percentage [95% bsCI]	89.27% [85.41, 92.57]	88.85% [84.60, 92.25]
eDOT Percentage [95% bsCI]	89.85% [85.30, 93.46]	89.78% [84.34, 93.70]
**Δ** Percentage= ipDOT – eDOT [95% bsCI]	−0.61% [−4.38, 4.04]	−0.80% [−5.53, 4.68]
eDOT Non-Inferior (Upper 95% bsCL **Δ** % ≤ +10%)	Yes	Yes
Summer: July - September		
ipDOT Percentage [95% bsCI]	86.11% [80.80, 90.04]	87.40% [82.65, 91.03]
eDOT Percentage [95% bsCI]	90.23% [86.72, 93.49]	89.36% [84.82, 93.28]
**Δ** Percentage= ipDOT – eDOT [95% bsCI]	−4.32% [−8.93, 0.17]	−2.00% [−7.30, 3.09]
eDOT Non-Inferior (Upper 95% bsCL **Δ** % ≤ +10%)	Yes	Yes
Autumn: October - December		
ipDOT Percentage [95% bsCI]	88.50% [84.33, 92.36]	87.78% [83.60, 91.66]
eDOT Percentage [95% bsCI]	89.30% [85.32, 93.03]	90.01% [85.81, 93.61]
**Δ** Percentage= ipDOT – eDOT [95% bsCI]	−0.80% [−5.62, 3.78]	−2.20% [−7.09, 3.19]
eDOT Non-Inferior (Upper 95% bsCL **Δ** % ≤ +10%)	Yes	Yes
Winter: January - March		
ipDOT Percentage [95% bsCI]	84.70% [79.88, 89.32]	84.72% [79.64, 89.56]
eDOT Percentage [95% bsCI]	89.64% [85.48, 93.41]	88.66% [84.34, 92.50]
**Δ** Percentage= ipDOT – eDOT [95% bsCI]	−4.86% [−9.98, −0.08]	−3.92% [−9.03, 0.62]
eDOT Non-Inferior (Upper 95% bsCL **Δ** % ≤ +10%)	Yes	Yes
Non-inferiority limit = +10%.<br>95% bsCI = 95% bootstrap confidence interval Bootstrap replicates = 1,000.		

© 2022 Burzynski J et al. *JAMA Network Open.*

eFigure. Dose Outcome Completed

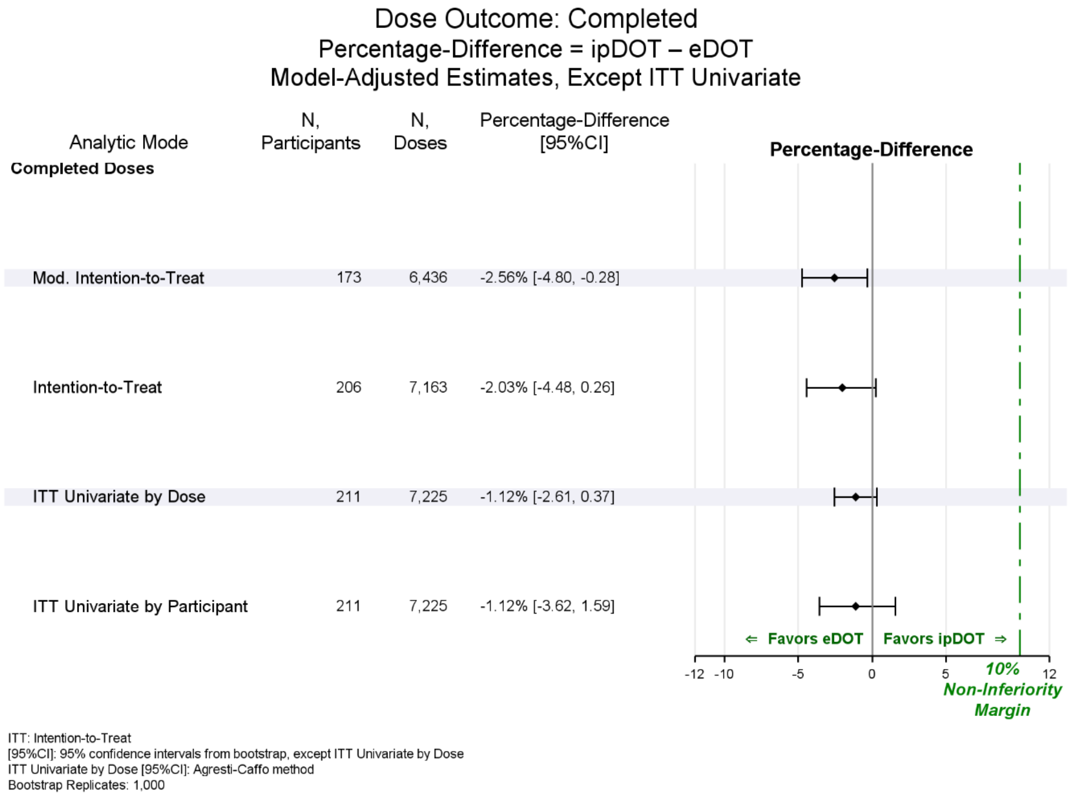

REFERENCES

1. Agresti A. *Categorical Data Analysis* 3rd ed.. Germany: Wiley, 2013.
2. SAS Software. Version 9.4. SAS Institute Inc. Cary, NC United States; 2012. Accessed 1 April 2021. SAS.com
3. Efron B, Tibshirani RJ. *An Introduction to the Bootstrap* 1st ed.. Chapman and Hall/CRC; 1994. https://doi.org/10.1201/9780429246593
4. Stroup, Walter W.. *Generalized Linear Mixed Models: Modern Concepts, Methods and Applications*. United Kingdom, CRC Press, 2016.
5. Agresti A, Caffo B. Simple and effective confidence intervals for proportions and differences of proportions result from adding two successes and two failures. *American Statistician.* 2000; 54:280–288. doi:10.1080/00031305.2000.10474560

© 2022 Burzynski J et al. *JAMA Network Open.*
