# Supplementary material for: In-Person vs Electronic Directly Observed Therapy for Tuberculosis Treatment Adherence: A Randomized Noninferiority Trial
Source: JAMA Netw Open. 2022 Jan 20;5(1):e2144210. doi: 10.1001/jamanetworkopen.2021.44210 (PMC8777548; doi:10.1001/jamanetworkopen.2021.44210)
Supplement: Supplement 3. — Nonauthor Collaborators. The eDOT Study Team nonauthor collaborators [file jamanetwopen-e2144210-s003.pdf]

Supplemental Online Content: Nonauthor Collaborators

*Indicates required information. Only first name, last name, and suffix will appear in PubMed.

*Group Name(s): The eDOT Study Team							
***First Name and Middle Initial(s)**	***Last Name**	***Suffix (eg, Jr, III)**	Academic Degrees	Institution	Location (city, state/province, country)	Role or Contribution, eg, chair, principal investigator	Group (if more than 1 Group listed in the byline) and/or Subgroup (eg, Steering Committee)
Sapna	Bamrah-Morris		MD MBA	U.S. Centers for Disease Control and Prevention	Atlanta, Georgia, USA	Investigator	
Sheridan	Bowers		BA	Columbia University	New York City, New York, USA	Clinical Research Coordinator	
Shannon	Carberry		MPH	U.S. Centers for Disease Control and Prevention	Atlanta, Georgia, USA	Research Associate	
Christine	Chuck		MPA	New York City Department of Health and Mental Hygiene, Bureau of TB Control	New York City, New York, USA	Investigator	
Matthew	Dias		MS	Columbia University	New York City, New York, USA	Clinical Research Coordinator	
Grace	Gao		MPH	New York City Department of Health and Mental Hygiene, Bureau of TB Control	New York City, New York, USA	Research Associate	
Richard	Garfein		PhD MPH	University of California - San Diego	La Jolla, California, USA	Consultant	
Vernard	Green		PhD	U.S. Centers for Disease Control and Prevention	Atlanta, Georgia, USA	Consultant	
Lon	Gross		BS	U.S. Centers for Disease Control and Prevention	Atlanta, Georgia, USA	Database Manager	
Gary	Henry		BS	New York City Department of Health and Mental Hygiene, Bureau of TB Control	New York City, New York, USA	Research Associate	
Andrew	Hill		PhD	U.S. Centers for Disease Control and Prevention	Atlanta, Georgia, USA	Consulting Statistician	
Sarah	Kiskadden-Bechtel		MS	Columbia University	New York City, New York, USA	Clinical Research Coordinator	
Meena	Lakshman		MPH	U.S. Centers for Disease Control and Prevention	Atlanta, Georgia, USA	Research Associate	

Supplemental Online Content: Nonauthor Collaborators

*Indicates required information. Only first name, last name, and suffix will appear in PubMed.

***First Name and Middle Initial(s)**	***Last Name**	***Suffix (eg, Jr, III)**	Academic Degrees	Institution	Location (city, state/province, country)	Role or Contribution, eg, chair, principal investigator	Group (if more than 1 Group listed in the byline) and/or Subgroup (eg, Steering Committee)
Nikolaos	Mitropoulos		MA	New York City Department of Health and Mental Hygiene, Bureau of TB Control	New York City, New York, USA	Research Associate	
Diana M	Nilsen		MD RN	New York City Department of Health and Mental Hygiene, Bureau of TB Control	New York City, New York, USA	Investigator	
Margaret	Oxtoby		MD	U.S. Centers for Disease Control and Prevention	Atlanta, Georgia, USA	Consultant	
Patrick	Philips		PhD	University of California - San Francisco	San Francisco, California, USA	Consulting Statistician	
Michael	Reaves		MS	Columbia University	New York City, New York, USA	Clinical Research Coordinator	
Errol	Robinson		MPA BSEE	New York City Department of Health and Mental Hygiene, Bureau of TB Control	New York City, New York, USA	Investigator	
Charlene	Sathi		MS	Columbia University	New York City, New York, USA	Clinical Research Coordinator	
Brock	Stewart		PhD	U.S. Centers for Disease Control and Prevention	Atlanta, Georgia, USA	Statistician	
Anila	Thomas		MPH	U.S. Centers for Disease Control and Prevention	Atlanta, Georgia, USA	Research Associate	
Zhanna	Tolochko			Columbia University	New York City, New York, USA	Administrative Coordinator	
Lisa	Trieu		MPH	New York City Department of Health and Mental Hygiene, Bureau of TB Control	New York City, New York, USA	Research Associate	
Carla	Winston		PhD	U.S. Centers for Disease Control and Prevention	Atlanta, Georgia, USA	Consultant	
