# Supplementary material for: In-Person vs Electronic Directly Observed Therapy for Tuberculosis Treatment Adherence: A Randomized Noninferiority Trial
Source: JAMA Netw Open. 2022 Jan 20;5(1):e2144210. doi: 10.1001/jamanetworkopen.2021.44210 (PMC8777548; doi:10.1001/jamanetworkopen.2021.44210)
Supplement: Supplement 4. — Data Sharing Statement [file jamanetwopen-e2144210-s004.pdf]

Burzynski J, Mangan JM, Lam CK, et al; eDOT Study Team. In-person vs electronic directly observed therapy for tuberculosis treatment adherence: a randomized noninferiority trial. *JAMA Netw Open.* 2022;5(1):e2144210. doi:10.1001/jamanetworkopen.2021.44210

Data Sharing Statement

Data

Data available: Yes
Data types: Deidentified participant data, Data dictionary
How to access data: De-identified participant data collected during this study, a data dictionary, and the study protocol will be made available with the publication of this article to researchers whose proposed use of the data is approved. Proposals should be directed to jburzyns@health.nyc.gov. To gain access, requesters will need to sign a data access agreement, and the de-identified database will be transferred through a secured drop box.
When available: With publication

Supporting Documents

Document types: None

Additional Information

Who can access the data: Researchers whose proposed use of the data has been approved
Types of analyses: Specified purpose
Mechanisms of data availability: With limited investigator support, after approval of a proposal, and a signed data access agreement
